# Supplementary material for: Associations of diarised sleep onset time, period and duration with total and central adiposity in a biethnic sample of young children: the Born in Bradford observational cohort study
Source: BMJ Open. 2021 May 25;11(5):e044769. doi: 10.1136/bmjopen-2020-044769 (PMC8154936; doi:10.1136/bmjopen-2020-044769)
Supplement: Author's manuscript [file bmjopen-2020-044769.draft_revisions.pdf]

BMJ Open

BMJ Open is committed to open peer review. As part of this commitment we make the peer review history of every article we publish publicly available.

When an article is published we post the peer reviewers' comments and the authors' responses online. We also post the versions of the paper that were used during peer review. These are the versions that the peer review comments apply to.

The versions of the paper that follow are the versions that were submitted during the peer review process. They are not the versions of record or the final published versions. They should not be cited or distributed as the published version of this manuscript.

BMJ Open is an open access journal and the full, final, typeset and author-corrected version of record of the manuscript is available on our site with no access controls, subscription charges or pay-per-view fees (http://bmjopen.bmj.com).

If you have any questions on BMJ Open's open peer review process please email info.bmjopen@bmj.com

BMJ Open

Associations of diarised sleep onset time, period and duration with total and central adiposity in a biethnic sample of young children: the Born in Bradford study

Journal:	*BMJ Open*
Manuscript ID	bmjopen-2020-044769
Article Type:	Original research
Date Submitted by the Author:	11-Sep-2020
Complete List of Authors:	Collings, Paul; University of York, Department of Health Sciences; Bradford Institute for Health Research,<br>Blackwell, Jane; University of York, Department of Health Sciences; Leeds and York Partnership NHS Foundation Trust<br>Pal, Elizabeth; Bradford Trident, Better Start Bradford<br>Wright, John; Bradford Institute for Health Research,<br>Ball, Helen; Durham University, Infancy & Sleep Centre & Departmenet of Anthropology
Keywords:	SLEEP MEDICINE, EPIDEMIOLOGY, Community child health < PAEDIATRICS, PREVENTIVE MEDICINE, PUBLIC HEALTH

SCHOLARONE™
Manuscripts

I, the Submitting Author has the right to grant and does grant on behalf of all authors of the Work (as defined in the below author licence), an exclusive licence and/or a non-exclusive licence for contributions from authors who are: i) UK Crown employees; ii) where BMJ has agreed a CC-BY licence shall apply, and/or iii) in accordance with the terms applicable for US Federal Government officers or employees acting as part of their official duties; on a worldwide, perpetual, irrevocable, royalty-free basis to BMJ Publishing Group Ltd ("BMJ") its licensees and where the relevant Journal is co-owned by BMJ to the co-owners of the Journal, to publish the Work in this journal and any other BMJ products and to exploit all rights, as set out in our licence.

The Submitting Author accepts and understands that any supply made under these terms is made by BMJ to the Submitting Author unless you are acting as an employee on behalf of your employer or a postgraduate student of an affiliated institution which is paying any applicable article publishing charge ("APC") for Open Access articles. Where the Submitting Author wishes to make the Work available on an Open Access basis (and intends to pay the relevant APC), the terms of reuse of such Open Access shall be governed by a Creative Commons licence – details of these licences and which Creative Commons licence will apply to this Work are set out in our licence referred to above.

Other than as permitted in any relevant BMJ Author's Self Archiving Policies, I confirm this Work has not been accepted for publication elsewhere, is not being considered for publication elsewhere and does not duplicate material already published. I confirm all authors consent to publication of this Work and authorise the granting of this licence.

For peer review only

Title: Associations of diarised sleep onset time, period and duration with total and central adiposity in a biethnic sample of young children: the Born in Bradford study

Authors: Paul J Collings[1,2], Jane E Blackwell[2,3], Elizabeth Pal[4], Helen L Ball[5], John Wright[1]

Affiliations: [1]Bradford Institute for Health Research, Bradford Teaching Hospitals NHS Foundation Trust, Bradford, England; [2]Department of Health Sciences, University of York, England; [3]Leeds and York Partnership NHS Foundation Trust, England; [4]Better Start Bradford, Bradford Trident, Bradford, England; [5]Infancy & Sleep Centre & Department of Anthropology, Department of Anthropology, Durham University, England

Corresponding author email: paul.collings@bthft.nhs.uk

ORCID: Paul J Collings: https://orcid.org/0000-0003-2022-5453; Jane E Blackwell: https://orcid.org/0000-0002-5878-8959; Elizabeth Pal: https://orcid.org/0000-0002-8682-3440; Helen L Ball: https://orcid.org/0000-0003-2255-121X; John Wright: https://orcid.org/0000-0001-9572-7293

Funding: The Born in Bradford study receives core infrastructure funding from the Wellcome Trust (grant number: WT101597MA), a joint grant from the UK Medical Research Council (MRC) and UK Economic and Social Science Research Council (ESRC) (MR/N024397/1), the British Heart Foundation (BHF) (CS/16/4/32482), and the National Institute for Health Research (NIHR) under its Collaboration for Applied Health Research and Care (CLAHRC) for Yorkshire and Humber. This study received delivery support from the NIHR Clinical Research Network. John Wright leads the Healthy Children, Healthy Families Theme of the NIHR CLAHRC in Yorkshire and Humber. Paul J Collings is funded by a BHF Immediate Postdoctoral Basic Science Research Fellowship (FS/17/37/32937). Jane E Blackwell coordinates and Paul J Collings is a member of The White Rose Child & Adolescent Sleep Research Network which is funded by a White Rose Collaboration Grant. The views expressed in this paper are those of the authors and not necessarily those of the MRC, ESRC, BHF, NIHR, and UK Department of Health or National Health Services or of any other funder acknowledged here.

Competing interests: None declared

Contributions: John Wright is Chief Investigator of the Born in Bradford study and setup the cohort; Helen L Ball created the parent-reported sleep diary; Paul J Collings initiated the study, planned and performed the statistical analyses, and wrote the manuscript; all authors critically revised the manuscript for intellectual content, helped interpret study findings, read and approved the final version and are accountable for the work.

Data availability: Data generated and analysed for the current study are available from the corresponding author on reasonable request. **Word count**: 4533

Abstract

Objectives: To investigate associations of sleep timing, period and duration with total and abdominal adiposity in a biethnic sample of children aged 18 and 36 months (m)

Design: Cross-sectional observational study

Setting: The Born in Bradford 1000 study, UK

Participants: Children aged approximately 18m (*n*=209; 40.2% South Asian; 59.8% White) and 36m (*n*=162; 40.7% South Asian; 59.3% White)

Primary and secondary outcome measures: Parents completed a 3-day sleep diary from which children's average daily sleep onset time, period and duration were calculated. Weekday to weekend differences in sleep parameters were also derived. As outcomes, indices of total (BMI z-score and sum of 2-skinfolds) and abdominal adiposity (waist circumference) were measured. Adjusted regression was used to quantify associations of sleep parameters with adiposity by age group and ethnicity.

Results: The average daily sleep onset time was markedly later in South Asian (9:26pm ± 68 mins) than White children (7:41pm ± 48 mins). Later sleep onset was associated with lower BMI z-score and sum of 2-skinfolds in White children aged 18m. In contrast, later sleep onset was associated with higher BMI z-score in South Asian children aged 36m. For weekday to weekend differences, longer sleep duration and later sleep onset on weekends than weekdays were both associated with higher total and abdominal adiposity in South Asian children aged 18m. On the contrary, compared to consistent sleep onset times, going to sleep ≥20 minutes later on weekends than weekdays was associated with lower waist circumference in White children aged 18m.

Conclusions: Sleep timing is associated with total and central adiposity in young children but associations differ by age group and ethnicity. Sleep onset times and regular sleep schedules may be important for obesity prevention.

For peer review only

Strengths and limitations of this study

- This is the first study to investigate myriad sleep dimensions and how they relate to direct markers of adiposity in young children
- It is also the first study to investigate differences in these associations between UK born South Asian and White children living in a deprived urban setting
- Sleep parameters were mutually adjusted for each other, and the analyses were adjusted for a broad range of covariates including potential mediating factors
- Residual confounding and mediating effects may have persisted because of measurement imprecision in parent-reported covariates, and directions of association between exposures and outcomes cannot be inferred from this cross-sectional study
- The proportion of parents who completed sleep diaries was low, meaning the study sample was fairly small and is susceptible to selection biases

For peer review only

Introduction

Many studies have investigated associations of sleep duration or the sleep period (here defined as elapsed time between sleep onset and termination regardless of waking intervals) with adiposity in school-aged children and youth [1,2]. Few such studies have been conducted in infants and toddlers despite sleep problems and sleep loss being prevalent in children aged <36 months (m) [3]. Beyond sleep duration, it is beginning to be recognised that sleep is a multidimensional construct of partly overlapping dimensions, and that parameters such as sleep timing may also be influential with regard to adiposity level and obesity risk [4].

Aspects of sleep timing include bedtimes, time of sleep onset, and variability in sleeping pattern such as differences between weekday and weekend sleep schedules. Preliminary evidence indicates that later bedtimes are associated with higher weight status in pre-schoolers [5–8] and primary school-aged children [9], and that later sleep timing on weekends than weekdays is correlated with higher risk of overweight and obesity [10]. Hypothesised mechanisms include circadian dysrhythmia causing hormone-mediated preferences for unhealthy calorific foodstuffs [11,12], the effect of which is compounded by later sleep onset enabling more time to eat, more opportunity for screen-based sedentary time such as TV viewing [13], and lower daily physical activity and higher sedentariness due to delayed waking and fatigue [14]. More studies need to investigate the independent associations of myriad sleep parameters with childhood adiposity and to ascertain the underlying mechanisms of action. A research focus on young children is important, to determine when associations between sleep parameters with adiposity first emerge, and to provide information about how to intervene by identifying modifiable target behaviours [4].

Early childhood is a critical window for obesity prevention because it is a period when rapid weight gain occurs and behavioural patterns are first established [15,16]. We are aware of only one study that has investigated associations of sleep duration and bedtimes with adiposity in early childhood. That study was conducted in children from America, Australia and New Zealand and the vast majority of mothers were of White ethnicity [17]. Investigation of ethnic minority and socioeconomically impoverished populations is needed. Children of non-White ethnicity and children from deprived backgrounds have unfavourable sleeping patterns and are high-risk for early onset obesity [18,19]. We have previously shown that UK South Asian children who live in a deprived urban setting go to bed much later than White children from the same area [20]. It is important to determine if shifted sleep schedules may in part explain why obesity rates are higher in South Asian than White children in the UK [21], and why South Asian school-aged children have more centrally-stored adiposity which is metabolically harmful [22]. This information could help reduce ethnic disparities in disease

For peer review only

risk by facilitating behaviour change interventions and policy guidelines that are tailored to the needs of specific ethnic groups.

This study examined independent associations of a broad range of sleep parameters including diarised sleep onset time, period and duration, and weekday to weekend variations in sleep parameters, with total and central adiposity in a young biethnic sample of UK children from a deprived urban setting.

Methods

This study was conducted in Bradford which is the fifth largest local authority in England and one of the most deprived and ethnically diverse cities in the UK [23]. Bradford has an overweight and obesity prevalence of 21.8% in 4 to 5 year old children and 38.4% in 10 to 11 year olds [24]. The Born in Bradford (BiB) 1000 study [25], nested within the larger BiB pregnancy cohort [26], aims to investigate modifiable risk factors for childhood obesity. Pregnant women (*n*=1916) were invited to the BiB 1000 study when they attended routine hospital appointments, 90.6% accepted the invitation (*n*=1735). Consent to medical records access was provided and periodic postnatal assessments were carried out when the women's offspring were approximately 6, 12, 18, 24, and 36m old. Parents who participated when children were aged about 18m (*n*=1228; 70.7% of all BiB 1000 participants) or 36m (*n*=1232; 71%) were asked to complete a sleep diary for their child. At each timepoint diaries were completed for approximately 15% of all BiB 1000 participants (18m: *n*=276; 36m: *n*=262). The diary data were collected between October 2010 and September 2012. This complete-case analysis included only children with sleep diary data, concurrent adiposity measurements and information about potentially important covariates. Maternal ethnicity was used as a proxy for child ethnicity, and children with a mother belonging to an ethnic group other than South Asian (Pakistani / Indian / Bangladeshi) or White (British / other) were excluded due to small numbers. All children were born in the UK, most children who were of South Asian heritage had Pakistani origin mothers (85%), and most children who were White had White British mothers (94%). For brevity we refer to children as being of South Asian or White ethnicity. The final sample included 209 children aged 18m (12% of all BiB 1000 participants) and 162 children aged 36m (9.3%). Ethical approval for all aspects of the study was granted by Bradford Research Ethics Committee (Reference 07/H1302/112) and all mothers provided informed written consent for participation.

Sleep parameters

Parents completed sleep diaries, providing free-text responses about the time their child fell asleep on an evening and woke-up the next morning. The sleep period was calculated as elapsed time between sleep onset and waking the next day. Because the sleep period does

For peer review only

not account for periods of overnight waking which can be common in young children [27] parents also reported their child's overnight sleep duration. Parents completed diaries on two weekdays and a weekend day. Mean weekday values were calculated by averaging data for the two weekdays; thereafter daily averages over the course of a week were calculated using weekday-weekend weighting (in the ratio of 5:2). Weekday to weekend differences in sleep parameters were calculated by subtraction (weekend – weekday values).

Adiposity markers

Child weight and height were assessed by trained researchers and BMI (kg/m^2) and *z*-scores were calculated [28]. Waist circumference was measured at the level of the navel. Skinfolds of the left triceps and subscapular were measured to the nearest 0.1mm and summed. Reliability metrics indicated good intra- and inter-observer technical error for measurements [29].

Covariates

Potential confounders and mediators of associations were selected based on previous evidence linking them with sleep and obesity [15,18]. A multidimensional marker of socioeconomic status was synthesised from information collated during interviews with parents (usually mothers) about their education, employment, housing tenure, financial situation, and ownership of goods. For the purposes of this study, from an initial five categories [30], children were classified as belonging to one of three parental socioeconomic status groups: least deprived (least socioeconomically deprived and most educated), moderately deprived (employed and not materially deprived / employed but no access to money) and most deprived (benefits but not materially deprived / most economically deprived). Maternal age was self-reported in pregnancy. The number of previous births (used to group mothers as primaparous (pregnant for the first time) or not), child gender, gestational age, and birth weight were extracted from medical records. Maternal height and weight were measured at the 18 and 36m timepoints and were used to calculate maternal BMI. Parents reported whether or not their child napped on sleep diary reporting days, these data were used to create three categories of napping frequency (never: napped zero days; occasionally: napped 1 or 2 days; every day: napped on all three days). On each sleep diary reporting day, parents also provided free-text responses about the time their child ate their last meal of the day. Mean weekday values were calculated by averaging data across the two weekdays, and weekday-weekend weighting was used to calculate the average daily time that the child ate their last meal; weekday to weekend differences were calculated by subtraction. Infant dietary data were collected using a validated food frequency questionnaire that was modified to include ethnic-specific foodstuffs; dietary constructs indicative of unhealthy snacking (frequency of biscuit, crisps, cakes, sweets, chocolate,

For peer review only

sugar-sweetened beverage consumption) and fruit and vegetable consumption in the previous 4-12 weeks were derived [20]. Parents reported the number of hours their child watched TV on a typical weekday and weekend day; weekday-weekend weighting was used to calculate average daily TV viewing and weekday to weekend differences were calculated [31]. When children were aged 36m a parent-reported Early Years Physical Activity Questionnaire (EY-PAQ) was used to estimate children's physical activity level [32]. Calendar dates of the 18 and 36m assessments were recorded and categorised by season (summer / autumn or spring / winter).

Patient and public involvement (PPI)

The BiB research team regularly convenes a Parent Governors PPI group, BiB participants now aged 10 to 12 years can become Young Ambassadors, and BiB runs regular community events and science festivals (https://borninbradford.nhs.uk/news-events/events/). These committees and events help to shape our research by allowing us to learn about the opinions, concerns and ideas of the community. A study author (JEB) also recently chaired a PPI event held in conjunction with The Sleep Charity [33]. Attendees were parents of children with sleep difficulties, staff who work in residential settings with children, sleep practitioners and representatives from parent and carer forums. Emerging themes from the session included: 1) there is a lack of information about assisting development of healthy sleep routines in infants, 2) more information and knowledge about the consequences of poor sleep in infants is needed, 3) early intervention is vital and parents of young children should be offered evidence-based sleep interventions for their child.

Statistics

Correlations between individual sleep parameters and adiposity markers were calculated using Pearson or Spearman methods as appropriate [34]. Adjusted linear regression was used to investigate cross-sectional associations between sleep parameters with adiposity. Model 1 adjusted associations for sex, age, socioeconomic status, maternal pregnancy age, parity, gestational age, birthweight, maternal BMI, season of measurement, and napping frequency. Model 2 further adjusted for potential confounding or mediating factors, including unhealthy snacking, fruit and vegetable intake, and TV viewing. Model 3 mutually adjusted sleep period and duration variables for sleep onset time, and vice versa. All analyses were performed stratified by ethnic group and age group. Interaction effects between sleep period and duration with sleep onset time were examined by introducing multiplicative terms to models (eg. sleep onset*duration). Nonlinearity of associations was examined by adding quadratic terms for sleep parameters. There was some of evidence for nonlinear associations between average daily sleep onset and weekday to weekend differences in sleep onset with waist circumference and sum of 2-skinfolds, hence sleep onset variables

For peer review only

were trichotomised and adjusted linear regression was used to estimate group marginal means (and 95% confidence intervals (CI)) for these outcomes. Data for the sum of 2-skinfolds were slightly skewed, but because results were consistent regardless of whether the data were log-transformed or not, results based on the untransformed data are presented. Sensitivity analyses further adjusted associations for the time of last meal and physical activity (not applicable to 18m models when physical activity was not assessed), potentially important covariates but missing data; models with waist circumference or sum of 2-skinfolds as dependent variables were further adjusted for height. Analyses were performed with Stata/SE 16.1 software (StataCorp, College Station, TX); *a priori* $p<0.05$ was deemed statistically significant but the results are interpreted with emphasis on the range of plausible values of associations as shown by confidence intervals [35].

Results

Sample characteristics

Participant details are shown in **Table 1**. Child ages ranged from 16.4 to 22.8m and 35.5 to 39.5m at each respective timepoint. The average daily sleep onset time was consistently after 9pm in South Asian children and before 8pm in White children. Sleep onset time was inversely correlated with the sleep period (South Asian children aged 18m: -0.52; South Asian children aged 36m: -0.39; White children aged 18m: -0.52; White children aged 36m: -0.37) and to a lesser extent sleep duration (South Asian children aged 18m: -0.41; South Asian children aged 36m: -0.10; White children aged 18m: -0.38; White children aged 36m: -0.27). Correlations between the sleep period and sleep duration were weaker in South Asian than White children (South Asian children aged 18m: 0.72; South Asian children aged 36m: 0.66; White children aged 18m: 0.81; White children aged 36m: 0.83). Correlations between adiposity markers differed by ethnic group and age, but consistently BMI z-score was more strongly correlated with waist circumference (South Asian children aged 18m: 0.69; South Asian children aged 36m: 0.71; White children aged 18m: 0.61; White children aged 36m: 0.79) than the sum of 2-skinfolds (South Asian children aged 18m: 0.48; South Asian children aged 36m: 0.28; White children aged 18m: 0.44; White children aged 36m: 0.53); correlations between waist circumference and the sum of 2-skinfolds were weakest (South Asian children aged 18m: 0.42; South Asian children aged 36m: 0.19; White children aged 18m: 0.26; White children aged 36m: 0.47).

South Asian children

Table 2 shows that in South Asian children aged 18m there were no significant associations between average daily sleep parameters with adiposity. In children aged 36m, there was some evidence in model 3 that later daily sleep onset was associated with higher BMI *z*-score; the association attenuated when further adjusted for final mealtime (β=0.2 (95% CI:-

For peer review only

0.2 to 0.6), *p*=0.34). There were no other significant associations for average daily sleep parameters. **Figure 1** presents the group marginal means for waist circumference and the sum of 2-skinfolds stratified by sleep onset categories; there were no group differences. With regard to weekday to weekend differences, Table 2 shows that longer sleep duration on weekends than weekdays was consistently associated with higher adiposity in South Asian children aged 18m, and in the same group of children, later sleep onset on weekends than weekdays was associated with larger waist circumference and sum of 2-skinfolds. In children aged 36m, later sleep onset on weekends than weekdays was associated with larger sum of 2-skinfolds in model 2, but the association attenuated when further adjusted for the sleep period. **Figure 2** shows the adjusted marginal means for waist circumference and sum of 2-skinfolds stratified by three categories of weekday to weekend differences in sleep onset. There was some evidence that compared to children with consistent sleep onset times (±20 minutes of each other), children aged 18m who went to sleep ≥20 minutes later on weekends than weekdays had larger waist circumferences (1.5 (-0.1 to 3.0) cm, *p*=0.060) and sum of 2-skinfolds (1.9 (-0.2 to 4.1) mm, *p*=0.078). Similarly, there was some indication that aged 36m, children who went to sleep ≥20 minutes later on weekends than weekdays had larger sum of 2-skinfolds (2.0 (-0.2 to 4.3) mm, *p*=0.074).

White children

Table 3 shows that for White children aged 36m there were no significant associations, although there was some indication that later sleep onset on weekends than weekdays was associated with smaller sum of 2-skinfolds. In children aged 18m, independent of all covariables including sleep period and duration, later daily sleep onset was associated with smaller sum of 2-skinfolds and lower BMI *z*-score (though further adjustment for final mealtime attenuated the latter association (-0.2 (-0.5 to 0.2), *p*=0.28)). Figure 1 illustrates that at age 18m, compared to children with a sleep onset time of 7:30pm or earlier, children who began to sleep between 7:30 and 8:30pm had smaller waist circumferences (-1.5 (-2.9 to -0.1) cm, *p*=0.035). Figure 2 highlights that children aged 18m who went to sleep ≥20 minutes later on weekends than weekdays, had smaller waist circumferences than children with consistent sleep onset times (-1.7 (-3.2 to -0.1) cm, *p*=0.038).

Sensitivity analyses

Further adjustment for physical activity in models that included children aged 36m, and height when waist circumference and the sum of 2-skinfolds were modelled as dependent variables, did not influence any of the reported associations. There was no evidence for interaction effects between sleep onset with the sleep period or sleep duration.

Discussion

For peer review only

This study investigated associations of diarised sleep onset time, period and duration with total and central adiposity in young children aged 18 and 36m. We discovered ethnicity- and age-specific associations for sleep onset times, which we primarily attribute to markedly different sleep schedules between South Asian and White children. Later daily sleep onset, and later sleep onset on weekends than weekdays, were both associated with higher adiposity in South Asian children and conversely lower adiposity in White children. Longer sleep duration on weekends than weekdays was associated with higher adiposity in South Asian children aged 18m.

Numerous systematic reviews have summarised the evidence for associations of sleep duration or the sleep period with adiposity in children and adolescents [1,2]. Of note, one review meta-analysed the results of six prospective studies totalling 14,264 children aged <36m. There was moderate heterogeneity in results, but each additional hour of sleep was associated with a negative change in BMI z-score over follow-up [36]. Limitations of the existing evidence include a preponderance of studies that have relied solely on BMI or BMI z-score as proxies for total adiposity. Studies have also considered few potential confounding or mediating factors and focussed solely on sleep duration. Sleep is a multidimensional construct and it is important to unravel the independent relations of specific dimensions with adiposity. In a previous BiB 1000 study we analysed repeated questionnaire data collected at four timepoints (12, 18, 24 and 36m) to quantify associations between parent reported sleep duration with measured adiposity in 1338 UK South Asian and White children. Longer sleep duration predicted lower total and central adiposity, but only in South Asian children [20]. Here, in a sub-sample of the same cohort, we were able to scrutinise and mutually adjust for myriad sleep parameters collected over the course of three days via parent-completed sleep diaries. Independent of sleep length, the average sleep onset time and variability in sleep timing between weekdays and weekends emerged as the key modifiable dimensions that predicted adiposity in South Asian and White children.

A recent review concluded that sleep timing, in particular later bedtimes, is associated with higher weight status in primary school-aged children [9]. Similarly, the few studies conducted thus far in 4 to 5 year olds indicate that later bedtimes, and most markedly bedtimes after 9pm, are associated with higher BMI z-score and obesity risk [5–7]; one study found that an association of short sleep with higher BMI z-score was only evident in children who went to bed after 9pm [8]. Just one study to date has been performed in younger children. Roy et al. found that later bedtimes were associated with higher odds of obesity in children aged 36m, but the association attenuated when adjusted for sleep duration [17]. To our knowledge, the current study is the first to show that independent of the sleep period, later sleep onset was associated with higher BMI z-score in South Asian children aged 36m. The association

For peer review only

attenuated when adjusted for final mealtime, which provides some indication that later sleep onset may elevate obesity risk via later evening meals and higher caloric intake later in the day [37]. In line with this hypothesis, animal models and clinical trials have shown that early and time-restricted feeding has numerous physiological benefits, including lower adiposity [38,39].

With regard to weekday and weekend differences, a recent review and pooled analysis of five studies in school-aged children and youth indicated that later sleep timing on weekends than weekdays was correlated with higher risk of overweight or obesity, albeit with a small effect size [10]. Our results extend this observation to younger children of South Asian heritage. We uniquely observed that later sleep timing on weekends than weekdays was associated with higher adiposity in South Asian children aged 18m and larger sum of 2-skinfolds in South Asian children aged 36m, although the association in older children only approached statistical significance. The associations in children aged 18m were independent of potential confounding or mediating factors including TV viewing, unhealthy snacking, fruit and vegetable intake, and the final mealtime. It may be that these are not important mediators in very young children, who are less autonomous, and for whom a large part of their diet and screen time is governed by parents and carers. That said, we cannot exclude residual confounding or mediation effects, as the data were all parent-reported and are subject to random error and bias. It is also unfortunate that physical activity was not measured at 18m. Longer sleep duration on weekends than weekdays was associated with higher adiposity at South Asian children aged 18m. This suggests that consistent sleep durations are important.

We observed that later daily sleep onset was associated with lower BMI z-score in White children aged 18m. This observation may appear counterintuitive, but Roy et al. similarly reported that independent of sleep duration, each one hour later to bed was associated with -0.08 lower BMI z-score in 878 children, although their confidence interval did narrowly cross the null of no association (95% CI: -0.17 to 0.01) [17]. Following adjustment for final mealtime our result for BMI z-score also attenuated to the null, but inverse associations with more direct adiposity indicators remained, including smaller sum of 2-skinfolds and waist circumference as a function of later sleep onset. These unexpected results may partly be explained by early sleep onset obstructing time during early-to-mid evenings that could otherwise be spent physically active. Time-stamped accelerometer studies show that daily physical activity patterns are bimodal in 18m olds. Physical activity levels first peak in the morning, slump after midday due to feeding and napping (81% of our White children aged 18m napped every day), after which there is a second larger peak that begins around 4pm and tails-off by 8pm [40,41]. In our sample of White children aged 18m, the earliest sleep

For peer review only

onset times were around 6pm and more than one-third (36.8%) were asleep by 7:30pm. It is conceivable that these children may have missed a substantial part of the evening peak in physical activity, thus contributing to higher adiposity [29,42]. Our related observation in the same group of children, that going to sleep ≥20 minutes later on weekends than weekdays was associated with lower waist circumference, may be explained by later sleep onset at the weekend enabling more physical activity [43].

Although White children aged 36m exhibited a similar distribution of sleep onset times compared to their younger White peers, there were few comparable associations, except some indication in children aged 36m that later sleep onset on weekends than weekdays was associated with lower adiposity (an association with the sum of 2-skinfolds approached statistical significance). These age-related differences may be explained by daily physical activity patterns following a different trajectory in slightly older children who tend not to nap (just 5% of White children aged 36m napped every day; 46% napped occasionally; 49% never napped). Instead of a naptime-related slump after midday which is followed by an evening peak, physical activity levels tend to be relatively constant throughout the afternoon, before progressively declining in the evening and most notably from 7pm onwards [44]. Hence, in many White children aged 36m, there may have been no peak in evening physical activity for early sleep to obstruct. In contrast to our results, Roy et al. did find a significant association between later bedtimes with lower BMI z-score in children aged approximately 36m [17]. That study was performed in children living in America, Australia and New Zealand, which likely explains the discrepancy. In those countries frequent daytime naps persist in older children partly because of designated naptimes in childcare [45–47], hence many children aged 3 to 4 years continue to exhibit a bimodal physical activity pattern, including an early-to-mid evening activity peak that early sleep could obstruct [48].

Sleep recommendations for children have historically focussed on duration rather than other sleep dimensions [49]. Our results highlight that contemporary sleep guidelines should further acknowledge the importance of sleep timing and regular sleep schedules [50]. Any such recommendations should also be considerate of ethnic and cultural differences in sleeping patterns, including what may and may not be feasible and acceptable to different population subgroups. Our data highlight that parents of White children should be made aware that going to sleep too early may be detrimental, certainly from an energy-balance and obesity risk perspective, and that going to sleep before 7:30pm might be inadvisable. Parents of South Asian children could be encouraged to set an earlier evening meal, an earlier sleep onset time, and to uphold consistent sleep schedules across week and weekend days. Studies of 4 to 5 year olds indicate that bedtimes before 9pm are important [5–8]. Our data also suggest that a reasonable and achievable target for young South Asian

For peer review only

children might be a sleep onset time before 9pm. It could be argued that any such changes in sleeping patterns may have only a trivial impact on adiposity, because the associations we have reported are modest in scale. However, our associations might be underestimated due to measurement imprecision and daily fluctuations in sleeping patterns. Furthermore, any influence on adiposity levels in early childhood may be important, it is a critical period in which rapid weight gain occurs and sustained obesity develops [16].

This study uniquely investigated associations of diarised sleep parameters with myriad adiposity markers in a biethnic sample of young children from a deprived urban setting, a high-risk group for sleep problems and obesity [18,19]. Sleep diaries are superior to the common practice of posing a single question to parents about their child's 'usual' sleep [51]. They do however incur participant burden and it is unfortunate that a minority of BiB 1000 parents completed sleep diaries for their child, the consequences of which are at least twofold. First, it is probable that our study sample is subject to selection biases which hinders generalisability. Second, stratifying by ethnicity and age group meant that some of our analyses were based on relatively few observations and low statistical power. This latter issue was compounded by missing data for specific items, such as sleep duration (which was only reported if parents considered they knew how long their child had slept overnight) and the sum of 2-skinfolds. It is nevertheless advantageous that we investigated more direct adiposity markers than suboptimal weight-for-height proxies and that in addition to skinfolds we investigated waist circumferences; young South Asian children eventually develop more centrally-stored adiposity which is metabolically deleterious [22]. Another strength is that each of our analyses were adjusted for a broad range of covariates, including mutual adjustment for sleep parameters to tease apart independent associations of distinct sleep dimensions with adiposity. Unfortunately, directions of association cannot be inferred from this cross-sectional study, it is possible that adiposity could influence sleeping habits rather than vice versa [20]. Longitudinal studies or trials are needed to determine the direction of associations. Future studies may achieve a better trade-off between participant retention and data resolution if they were to use sensor-based technologies rather than diaries [52], alternatively a combination of methods may prove best to capture habitual sleep parameters [53].

Conclusions

Sleep onset time appears to be independently associated with levels of total and central adiposity in young South Asian and White children. Suitable sleep times and consistent sleep schedules across week and weekend days could be important modifiable determinants of early onset obesity.

For peer review only

References

1 Matricciani L, Paquet C, Galland B, *et al.* Children's sleep and health: A meta-review. *Sleep Med Rev* 2019;**46**:136–50. doi:10.1016/j.smrv.2019.04.011

2 Sun J, Wang M, Yang L, *et al.* Sleep duration and cardiovascular risk factors in children and adolescents: A systematic review. *Sleep Med Rev* 2020;**53**:101338. doi:10.1016/j.smrv.2020.101338

3 Ophoff D, Slaats MA, Boudewyns A, *et al.* Sleep disorders during childhood: a practical review. *Eur J Pediatr* 2018;**177**:641–8. doi:10.1007/s00431-018-3116-z

4 Miller AL, Lumeng JC, LeBourgeois MK. Sleep patterns and obesity in childhood. *Curr Opin Endocrinol diabetes Obes* 2015;**22 SRC-**:41–7.

5 Scharf RJ, Deboer MD. Sleep timing and longitudinal weight gain in 4- and 5-year-old children. *Pediatr Obes* 2015;**10**:141–8. doi:10.1111/ijpo.229

6 Anderson SE, Andridge R, Whitaker RC. Bedtime in Preschool-Aged Children and Risk for Adolescent Obesity. *J Pediatr* 2016;**176**:17–22. doi:10.1016/j.jpeds.2016.06.005

7 Simon SL, Goetz AR, Meier M, *et al.* Sleep duration and bedtime in preschool-age children with obesity: Relation to BMI and diet following a weight management intervention. *Pediatr Obes* 2019;**14**:1–9. doi:10.1111/ijpo.12555

8 Miller AL, Kaciroti N, Lebourgeois MK, *et al.* Sleep timing moderates the concurrent sleep duration-body mass index association in low-income preschool-age children. *Acad Pediatr* 2014;**14**:207–13. doi:10.1016/j.acap.2013.12.003

9 Morrissey B, Taveras E, Allender S, *et al.* Sleep and obesity among children: A systematic review of multiple sleep dimensions. *Pediatr Obes* 2020;**15**:1–21. doi:10.1111/ijpo.12619

10 Sun W, Ling J, Zhu X, *et al.* Associations of weekday-to-weekend sleep differences with academic performance and health-related outcomes in school-age children and youths. *Sleep Med Rev* 2019;**46**:27–53. doi:10.1016/j.smrv.2019.04.003

11 Petrov ME, Vander Wyst KB, Whisner CM, *et al.* Relationship of Sleep Duration and Regularity with Dietary Intake Among Preschool-Aged Children with Obesity from Low-Income Families. *J Dev Behav Pediatr* 2017;**38**:120–8. doi:10.1097/DBP.0000000000000369

12 Jansen EC, Peterson KE, Lumeng JC, *et al.* Associations between Sleep and Dietary Patterns among Low-Income Children Attending Preschool. *J Acad Nutr Diet*

For peer review only

2019;**119**:1176–87. doi:10.1016/j.jand.2019.01.008

13 Moorman JD, Morgan P, Adams TL. The Implications of Screen Media Use for the Sleep Behavior of Children Ages 0–5: a Systematic Review of the Literature. *Curr Sleep Med Reports* 2019;**5**:164–72. doi:10.1007/s40675-019-00151-0

14 Olds TS, Maher CA, Matricciani L. Sleep Duration or Bedtime? Exploring the Relationship between Sleep Habits and Weight Status and Activity Patterns. *Sleep* 2011;**34**:1299–307. doi:10.5665/sleep.1266

15 Woo Baidal JA, Locks LM, Cheng ER, *et al.* Risk Factors for Childhood Obesity in the First 1,000 Days. *Am J Prev Med* 2016;**50**:761–79. doi:10.1016/j.amepre.2015.11.012

16 Geserick M, Vogel M, Gausche R, *et al.* Acceleration of BMI in Early Childhood and Risk of Sustained Obesity. *N Engl J Med* 2018;**379**:1303–12. doi:10.1056/NEJMoa1803527

17 Roy M, Haszard JJ, Savage JS, *et al.* Bedtime, body mass index and obesity risk in preschool-aged children. *Pediatr Obes* 2020;:ijpo.12650. doi:10.1111/ijpo.12650

18 Blair PS, Humphreys JS, Gringras P, *et al.* Childhood Sleep Duration and Associated Demographic Characteristics in an English Cohort. *Sleep* 2012;**35**:353–60. doi:10.5665/sleep.1694

19 Lu Y, Pearce A, Li L. Distinct patterns of socio-economic disparities in child-to-adolescent BMI trajectories across UK ethnic groups: A prospective longitudinal study. *Pediatr Obes* 2020;**15**:1–9. doi:10.1111/ijpo.12598

20 Collings PJ, Ball HL, Santorelli G, *et al.* Sleep Duration and Adiposity in Early Childhood: Evidence for Bidirectional Associations from the Born in Bradford Study. *Sleep* 2017;**40**. doi:10.1093/sleep/zsw054

21 Hudda MT, Nightingale CM, Donin AS, *et al.* Patterns of childhood body mass index (BMI), overweight and obesity in South Asian and black participants in the English National child measurement programme: effect of applying BMI adjustments standardising for ethnic differences in BMI-body fatness asso. *Int J Obes* 2018;**42**:662–70. doi:10.1038/ijo.2017.272

22 Wolf RM, Nagpal M, Magge SN. Diabetes and cardiometabolic risk in south Asian youth: A review. *Pediatr Diabetes* 2020;**Online**:1–15. doi:10.1111/pedi.13078

23 City of Bradford Metropolitan District Council. Understanding Bradford District. 2020.https://ubd.bradford.gov.uk/ (accessed 22 Apr 2020).

24 Local Government Association (LGA) Research. National Child Measurement

For peer review only

Programme data for Bradford. https://lginform.local.gov.uk/reports/view/lga-research/latest-data-from-the-national-child-measurement-programme-ncmp?mod-area=E08000032&mod-group=AllRegions_England&mod-type=namedComparisonGroup (accessed 23 Apr 2020).

25 Bryant M, Santorelli G, Fairley L, *et al.* Design and characteristics of a new birth cohort, to study the early origins and ethnic variation of childhood obesity: the BiB1000 study. *Longit Life Course Stud* 2013;**4**:119–35. doi:10.14301/llcs.v4i2.221

26 Wright J, Small N, Raynor P, *et al.* Cohort profile: The born in bradford multi-ethnic family cohort study. *Int J Epidemiol* 2013;**42**:978–91. doi:10.1093/ije/dys112

27 Sadeh A, Mindell JA, Luedtke K, *et al.* Sleep and sleep ecology in the first 3 years: A web-based study. *J Sleep Res* Published Online First: 2009. doi:10.1111/j.1365-2869.2008.00699.x

28 Cole TJ, Freeman JV, Preece MA. Body mass index reference curves for the UK, 1990. *Arch Dis Child* 1995;**73**:25–9. doi:10.1136/adc.73.1.25

29 Collings PJ, Brage S, Bingham DD, *et al.* Physical Activity, Sedentary Time, and Fatness in a Biethnic Sample of Young Children. *Med Sci Sport Exerc* 2017;**49**:930–8. doi:10.1249/MSS.0000000000001180

30 Fairley L, Cabieses B, Small N, *et al.* Using latent class analysis to develop a model of the relationship between socioeconomic position and ethnicity: cross-sectional analyses from a multi-ethnic birth cohort study. *BMC Public Health* 2014;**14**:835. doi:10.1186/1471-2458-14-835

31 Collings PJ, Kelly B, West J, *et al.* Associations of TV Viewing Duration, Meals and Snacks Eaten When Watching TV, and a TV in the Bedroom with Child Adiposity. *Obesity* 2018;**26**:1619–28. doi:10.1002/oby.22288

32 Bingham D, Collings P, Clemes S, *et al.* Reliability and Validity of the Early Years Physical Activity Questionnaire (EY-PAQ). *Sports* 2016;**4**:30. doi:10.3390/sports4020030

33 The Sleep Charity. https://thesleepcharity.org.uk/ (accessed 28 Aug 2020).

34 de Winter JCF, Gosling SD, Potter J. Comparing the Pearson and Spearman correlation coefficients across distributions and sample sizes: A tutorial using simulations and empirical data. *Psychol Methods* 2016;**21**:273–90. doi:10.1037/met0000079

35 Amrhein V, Greenland S, McShane B. Scientists rise up against statistical significance. *Nature* 2019;**567**:305–7. doi:10.1038/d41586-019-00857-9

36 Miller MA, Kruisbrink M, Wallace J, *et al.* Sleep duration and incidence of obesity in

For peer review only

infants, children, and adolescents: a systematic review and meta-analysis of prospective studies. *Sleep* 2018;**41**:1–19. doi:10.1093/sleep/zsy018

37 Almoosawi S, Vingeliene S, Karagounis LG, *et al.* Chrono-nutrition: A review of current evidence from observational studies on global trends in time-of-day of energy intake and its association with obesity. *Proc Nutr Soc* 2016;**75**:487–500. doi:10.1017/S0029665116000306

38 Sutton EF, Beyl R, Early KS, *et al.* Early Time-Restricted Feeding Improves Insulin Sensitivity, Blood Pressure, and Oxidative Stress Even without Weight Loss in Men with Prediabetes. *Cell Metab* 2018;**27**:1212-1221.e3. doi:10.1016/j.cmet.2018.04.010

39 de Cabo R, Mattson MP. Effects of Intermittent Fasting on Health, Aging, and Disease. *N Engl J Med* 2019;**381**:2541–51. doi:10.1056/NEJMra1905136

40 Hnatiuk J, Ridgers ND, Salmon J, *et al.* Physical activity levels and patterns of 19-month-old children. *Med Sci Sports Exerc* Published Online First: 2012. doi:10.1249/MSS.0b013e31825825c4

41 Prioreschi A, Brage S, Hesketh KD, *et al.* Describing objectively measured physical activity levels, patterns, and correlates in a cross sectional sample of infants and toddlers from South Africa. *Int J Behav Nutr Phys Act* 2017;**14**:176. doi:10.1186/s12966-017-0633-5

42 Collings PJ, Brage S, Ridgway CL, *et al.* Physical activity intensity, sedentary time, and body composition in preschoolers. *Am J Clin Nutr* Published Online First: 2013. doi:10.3945/ajcn.112.045088

43 Collings PJ, Dogra SA, Costa S, *et al.* Objectively-measured sedentary time and physical activity in a bi-ethnic sample of young children: variation by socio-demographic, temporal and perinatal factors. *BMC Public Health* 2020;**20**:109. doi:10.1186/s12889-019-8132-z

44 Hesketh KR, McMinn AM, Ekelund U, *et al.* Objectively measured physical activity in four-year-old British children: a cross-sectional analysis of activity patterns segmented across the day. *Int J Behav Nutr Phys Act* 2014;**11**:1. doi:10.1186/1479-5868-11-1

45 Ward TM, Gay C, Anders TF, *et al.* Sleep and Napping Patterns in 3-to-5-year old Children Attending Full-Day Childcare Centers. *J Pediatr Psychol* 2008;**33**:666–72. doi:10.1093/jpepsy/jsm102

46 Staton SL, Smith SS, Hurst C, *et al.* Mandatory Nap Times and Group Napping Patterns in Child Care: An Observational Study. *Behav Sleep Med* 2017;**15**:129–43.

For peer review only

doi:10.1080/15402002.2015.1120199

47 Staton S, Rankin PS, Harding M, *et al.* Many naps, one nap, none: A systematic review and meta-analysis of napping patterns in children 0–12 years. *Sleep Med Rev* 2020;**50**:101247. doi:10.1016/j.smrv.2019.101247

48 Dias K, White J, Jago R, *et al.* International Comparison of the Levels and Potential Correlates of Objectively Measured Sedentary Time and Physical Activity among Three-to-Four-Year-Old Children. *Int J Environ Res Public Health* 2019;**16**:1929. doi:10.3390/ijerph16111929

49 Matricciani LA, Olds TS, Blunden S, *et al.* Never Enough Sleep: A Brief History of Sleep Recommendations for Children. *Pediatrics* 2012;**129**:548–56. doi:10.1542/peds.2011-2039

50 World Health Organization. Guidelines on physical activity, sedentary behaviour and sleep for children under 5 years of age. 2019. http://www.who.int/iris/handle/10665/311664

51 Matricciani L. Subjective reports of children's sleep duration: Does the question matter? A literature review. *Sleep Med* 2013;**14**:303–11. doi:10.1016/j.sleep.2013.01.002

52 Schoch SF, Kurth S, Werner H. Actigraphy in sleep research with infants and young children: Current practices and future benefits of standardized reporting. *J Sleep Res* 2020;:1–15. doi:10.1111/jsr.13134

53 Collings PJ, Wijndaele K, Corder K, *et al.* Prospective associations between sedentary time, sleep duration and adiposity in adolescents. *Sleep Med* 2015;**16**:717–22. doi:10.1016/j.sleep.2015.02.532

For peer review only

Table 1. Description of study participants

	South Asian		White	
	18 months (*n*=84)	36 months (*n*=66)	18 months (*n*=125)	36 months (*n*=96)
Sex (*n* (%) boys)	43 (51.2)	32 (48.5)	58 (46.4)	38 (39.6)
Age (months)	18.1 (0.7)	36.7 (1.1)	18.3 (1.1)	36.5 (0.5)
Parental socioeconomic status (*n* (%))				
Least deprived	22 (26.2)	15 (22.7)	32 (25.6)	30 (31.3)
Moderately deprived	29 (34.5)	20 (30.3)	70 (56.0)	53 (55.2)
Most deprived	33 (39.3)	31 (47.0)	23 (18.4)	13 (13.5)
Maternal ethnicity (*n* (%) Pakistani origin)	73 (86.9)	55 (83.3)	-	-
Maternal ethnicity (*n* (%) White British)	-	-	119 (95.2)	89 (92.7)
Maternal pregnancy age (y)	27.6 (7.3)	30.6 (7.6)	29.1 (10.3)	28.8 (8.2)
Parity (*n* (%) primaparous)	30 (35.7)	21 (31.8)	65 (52.0)	60 (62.5)
Birthweight (g)	3156 ± 465	3066 ± 552	3400 ± 543	3358 ± 563
Gestational age (weeks)	39.6 ± 1.6	39.4 ± 1.6	39.9 ± 1.5	39.6 ± 1.9
Season (*n* (%) summer / autumn)	71 (84.5)	22 (33.3)	108 (86.4)	18 (18.8)
Unhealthy snacks (*n*/week)	10 (10)	17 (23)	9 (8)	11 (12)
Fruit & vegetables (portions/day)	6 ± 2	6 ± 3	5 ± 2	5 ± 3
Physical activity (h/day)	-	2.8 ± 1.5	-	2.7 (2.0)
Maternal BMI (kg/m^2)	26.4 (6.3)	27.2 (6.6)	25.1 (8.0)	25.8 (7.0)
Napping frequency (*n* (%))				
Never	0 (0)	24 (36.4)	4 (3.2)	47 (49.0)
Occasionally	7 (8.3)	26 (39.4)	20 (16.0)	44 (45.8)
Everyday	77 (91.7)	16 (24.2)	101 (80.8)	5 (5.2)
Weighted daily average				
TV viewing (h/day)	1.0 (1.6)	1.5 (1.7)	0.6 (1.0)	1.5 (1.1)
Time of last meal (pm)	7:20 ± 69	7:20 ± 59	5:28 ± 51	5:32 ± 43
Sleep onset (pm)	9:36 ± 69	9:12 ± 63	7:50 ± 50	7:54 ± 44
Sleep offset (am)	8:21 ± 63	8:23 ± 63	7:11 ± 47	7:12 ± 50
Sleep period (h/night)	10.8 ± 1.0	11.2 ± 0.8	11.4 ± 0.8	11.3 ± 0.7
Sleep duration (h/night)	10.5 ± 1.0	11.0 ± 1.0	11.1 ± 1.1	11.3 ± 0.8
Weekday to weekend difference				
TV viewing (h/day)	-0.1 ± 0.6	0.0 ± 0.6	0.0 ± 0.6	0.0 ± 0.9
Time of last meal (h)	0.2 ± 0.7	0.2 ± 0.7	0.1 ± 0.8	0.2 ± 0.7
Sleep onset (h)	0.1 ± 0.6	0.3 ± 0.8	0.1 ± 0.9	0.1 ± 0.8
Sleep offset (h)	0.2 ± 0.9	0.3 ± 0.8	0.3 ± 0.8	0.2 ± 0.9
Sleep period (h/night)	0.1 ± 1.0	0.0 ± 0.8	0.2 ± 1.2	0.1 ± 1.0
Sleep duration (h/night)	0.0 ± 0.9	-0.1 ± 1.0	0.0 ± 1.0	0.0 ± 1.2
BMI (kg/m^2)	16.0 ± 1.3	15.8 ± 1.2	16.7 ± 1.2	16.4 ± 1.2
BMI *z*-score	-0.8 ± 1.1	-0.2 ± 1.0	-0.2 ± 0.9	0.3 ± 0.8
Waist circumference (cm)	44.9 ± 2.8	49.2 ± 3.1	46.3 ± 3.0	50.1 ± 3.1
Sum of 2-skinfolds (mm)	17.1 (3.8)	15.8 (3.4)	17.4 (4.3)	17.2 (4.8)

Normally distributed continuous variables are described as mean ± sd. Time of last meal, sleep onset and offset are described as clock times ± sd in minutes. Non-normally distributed continuous variables are described as median (IQR). Positive weekday to weekend differences indicate more TV viewing or sleep at the weekend than on weekdays, or later clock times at the weekend. There were missing data: Physical activity available for *n*=60 South Asian children at 36 months and *n*=89 White children at 36 months; Time of last meal available for *n*=71 South Asian children at 18 months, *n*=59 South Asian children at 36 months, *n*=119 White children at 18 months, *n*=91 White children at 36 months; Sleep duration available for *n*=63 South Asian children at 18 months, *n*=45 South Asian children at 36 months, *n*=108 White children at 18 months, *n*=90 White children at 36 months; Waist circumference available for *n*=77 South Asian children at 18 months, *n*=60 South Asian children at 36 months, *n*=109 White children at 18 months, *n*=96 White children at 36 months; Sum of 2-skinfolds available for *n*=64 South Asian children at 18 months, *n*=38 South Asian children at 36 months, *n*=84 White children at 18 months, *n*=67 White children at 36 months. BMI, Body mass index

For peer review only

Table 2. Associations of sleep onset, period and duration with adiposity in South Asian children, stratified by age group

Age group		Body mass index *z*-score					Waist circumference (cm)					Sum of 2-skinfolds (mm)				
			Weighted daily average		Weekday to weekend difference			Weighted daily average		Weekday to weekend difference			Weighted daily average		Weekday to weekend difference	
		n	β (95% CI)	*p*	β (95% CI)	*p*	*n*	β (95% CI)	*p*	β (95% CI)	*p*	*n*	β (95% CI)	*p*	β (95% CI)	*p*
18 months	Model 1															
	Sleep onset	84	-0.1 (-0.3 to 0.2)	0.66	0.1 (-0.4 to 0.5)	0.76	77	-0.4 (-1.0 to 0.2)	0.24	0.8 (-0.3 to 1.8)	0.17	64	-0.2 (-1.1 to 0.7)	0.68	1.3 (-0.1 to 2.8)	0.070
	Sleep period	84	0.2 (-0.1 to 0.4)	0.16	0.0 (-0.3 to 0.3)	0.84	77	0.3 (-0.3 to 1.0)	0.30	0.0 (-0.7 to 0.8)	0.91	64	0.2 (-0.7 to 1.0)	0.70	-0.3 (-1.4 to 0.8)	0.56
	Sleep duration	63	0.2 (-0.2 to 0.5)	0.29	0.3 (-0.1 to 0.6)	0.17	58	0.3 (-0.6 to 1.2)	0.54	0.6 (-0.4 to 1.7)	0.21	49	0.5 (-0.7 to 1.6)	0.41	0.8 (-0.6 to 2.2)	0.26
	Model 2															
	Sleep onset	84	0.0 (-0.2 to 0.3)	0.93	0.0 (-0.4 to 0.4)	0.93	77	-0.3 (-0.9 to 0.4)	0.41	0.7 (-0.4 to 1.8)	0.21	64	-0.1 (-0.9 to 0.8)	0.90	**1.6 (0.1 to 3.1)**	**0.036**
	Sleep period	84	0.2 (-0.1 to 0.4)	0.22	0.1 (-0.2 to 0.4)	0.59	77	0.3 (-0.4 to 1.0)	0.39	0.3 (-0.5 to 1.1)	0.47	64	0.1 (-0.8 to 1.0)	0.89	-0.2 (-1.4 to 0.9)	0.72
	Sleep duration	63	0.2 (-0.1 to 0.5)	0.27	0.3 (0.0 to 0.7)	0.066	58	0.3 (-0.6 to 1.2)	0.55	0.9 (-0.1 to 1.9)	0.075	49	0.3 (-0.9 to 1.5)	0.61	0.9 (-0.6 to 2.3)	0.23
	Model 3															
	Sleep onset[1]	84	0.1 (-0.2 to 0.4)	0.40	0.0 (-0.5 to 0.5)	0.99	77	-0.2 (-0.9 to 0.6)	0.65	1.2 (-0.1 to 2.4)	0.076	64	0.0 (-1.1 to 1.1)	0.96	**1.8 (0.1 to 3.5)**	**0.034**
	Sleep onset[2]	63	0.2 (-0.1 to 0.5)	0.23	0.4 (-0.1 to 0.8)	0.12	58	0.2 (-0.6 to 1.1)	0.58	**1.8 (0.6 to 2.9)**	**0.004**	49	0.0 (-0.9 to 1.1)	0.92	**1.7 (0.3 to 3.1)**	**0.022**
	Sleep period	84	0.2 (-0.1 to 0.5)	0.14	0.1 (-0.2 to 0.4)	0.63	77	0.2 (-0.6 to 1.0)	0.60	0.7 (-0.2 to 1.5)	0.14	64	0.0 (-1.1 to 1.1)	0.93	0.3 (-0.9 to 1.5)	0.61
	Sleep duration	63	0.3 (-0.1 to 0.6)	0.14	**0.4 (0.1 to 0.8)**	**0.024**	58	0.3 (-0.6 to 1.3)	0.46	**1.2 (0.3 to 2.2)**	**0.012**	49	0.3 (-0.9 to 1.5)	0.61	1.4 (0.0 to 2.9)	0.056
36 months	Model 1															
	Sleep onset	66	0.1 (-0.1 to 0.4)	0.17	-0.3 (-0.6 to 0.0)	0.085	60	-0.1 (-1.0 to 0.7)	0.74	**-1.2 (-2.4 to 0.0)**	**0.048**	38	-0.3 (-1.3 to 0.7)	0.52	0.9 (-0.3 to 2.1)	0.14
	Sleep period	66	0.1 (-0.2 to 0.4)	0.50	0.2 (-0.1 to 0.5)	0.14	60	-0.4 (-1.7 to 0.8)	0.48	**1.1 (0.0 to 2.3)**	**0.047**	38	0.0 (-1.2 to 1.3)	0.96	-0.3 (-1.8 to 1.1)	0.64
	Sleep duration	45	0.2 (-0.1 to 0.5)	0.15	0.0 (-0.4 to 0.3)	0.82	42	0.5 (-0.6 to 1.5)	0.35	0.3 (-1.0 to 1.6)	0.63	26	0.2 (-0.9 to 1.2)	0.71	-0.1 (-1.8 to 1.6)	0.92
	Model 2															
	Sleep onset	66	0.2 (-0.1 to 0.4)	0.13	-0.2 (-0.6 to 0.1)	0.15	60	0.1 (-0.8 to 1.0)	0.86	-1.0 (-2.2 to 0.2)	0.11	38	-0.2 (-1.4 to 1.0)	0.75	**1.3 (0.1 to 2.5)**	**0.033**
	Sleep period	66	0.1 (-0.2 to 0.5)	0.42	0.2 (-0.1 to 0.5)	0.14	60	-0.4 (-1.7 to 0.9)	0.55	**1.2 (0.1 to 2.3)**	**0.032**	38	0.1 (-1.2 to 1.4)	0.84	-1.1 (-2.7 to 0.4)	0.15
	Sleep duration	45	0.2 (-0.1 to 0.5)	0.27	0.1 (-0.3 to 0.5)	0.67	42	0.3 (-0.8 to 1.4)	0.55	0.5 (-0.8 to 1.9)	0.44	26	0.0 (-1.1 to 1.0)	0.97	-0.8 (-3.1 to 1.5)	0.43
	Model 3															
	Sleep onset[1]	**66**	**0.3 (0.0 to 0.5)**	**0.042**	-0.2 (-0.6 to 0.2)	0.27	60	0.0 (-1.0 to 0.9)	0.97	-0.2 (-1.7 to 1.3)	0.80	38	-0.2 (-1.5 to 1.1)	0.79	1.2 (-0.3 to 2.8)	0.12
	Sleep onset[2]	45	0.1 (-0.1 to 0.4)	0.34	-0.2 (-0.8 to 0.3)	0.43	42	-0.3 (-1.2 to 0.6)	0.53	0.0 (-1.9 to 2.0)	0.95	26	-0.8 (-2.2 to 0.7)	0.25	0.4 (-2.3 to 3.1)	0.72
	Sleep period	66	0.3 (-0.1 to 0.6)	0.12	0.1 (-0.2 to 0.5)	0.42	60	-0.4 (-1.8 to 1.0)	0.57	1.1 (-0.3 to 2.5)	0.11	38	0.1 (-1.3 to 1.5)	0.89	-0.4 (-2.2 to 1.4)	0.68
	Sleep duration	45	0.2 (-0.1 to 0.5)	0.23	0.0 (-0.4 to 0.4)	0.85	42	0.3 (-0.8 to 1.4)	0.55	0.5 (-0.9 to 2.0)	0.45	26	-0.1 (-1.2 to 0.9)	0.80	-0.2 (-3.4 to 2.9)	0.85

Results are beta-coefficients (95% confidence intervals) and represent the difference in outcomes for every one hour later to sleep, one hour longer sleep period or duration, one hour later to sleep on weekends than weekdays, or one hour longer sleep period or duration on weekends than weekdays, as appropriate. Model 1 was adjusted for sex, age, socioeconomic status, maternal pregnancy age, parity, gestational age, birthweight, maternal body mass index, season of measurement, and napping frequency (models for weekday to weekend differences were further adjusted for the average weekday value of the exposure variable). Model 2 was further adjusted for unhealthy snacking, fruit and vegetable intake, and TV viewing (models for weekday to weekend sleep differences were adjusted for average weekday TV viewing and weekday to weekend differences in TV viewing). Model 3 further adjusted the sleep period and duration for sleep onset, and vice versa (models for weekday to weekend differences were mutually adjusted for average weekday and weekday to weekend differences in the sleep period, duration or onset as appropriate). [1]Adjusted for the sleep period; [2]Adjusted for sleep duration. Statistically significant results ($p<0.05$) are highlighted bold. Sensitivity analyses confirmed that adjustment for physical activity (collected at 36 months only) did not confound Model 3 results, but the association between weighted daily average sleep onset with body mass index *z*-score at 36 months was attenuated when adjusted for time of last meal (body mass index *z*-score (n=59): 0.2 (-0.2 to 0.6), p=0.34).

For peer review only

For peer review only - http://bmjopen.bmj.com/site/about/guidelines.xhtml

Table 3. Associations of sleep onset, period and duration with adiposity in White children, stratified by age group

		Body mass index *z*-score					Waist circumference (cm)					Sum of 2-skinfolds (mm)				
			Weighted daily average		Weekday to weekend difference			Weighted daily average		Weekday to weekend difference			Weighted daily average		Weekday to weekend difference	
		n	β (95% CI)	*p*	β (95% CI)	*p*	*n*	β (95% CI)	*p*	β (95% CI)	*p*	*n*	β (95% CI)	*p*	β (95% CI)	*p*
18 months	Model 1															
	Sleep onset	125	-0.2 (-0.4 to 0.0)	0.086	0.0 (-0.2 to 0.2)	0.70	109	-0.5 (-1.2 to 0.2)	0.18	-0.6 (-1.4 to 0.2)	0.17	84	-1.0 (-2.1 to 0.0)	0.061	-0.1 (-1.2 to 1.0)	0.88
	Sleep period	125	0.0 (-0.2 to 0.2)	0.83	0.1 (-0.1 to 0.2)	0.44	109	0.2 (-0.6 to 0.9)	0.64	0.1 (-0.5 to 0.7)	0.70	84	0.1 (-1.0 to 1.2)	0.82	-0.1 (-0.9 to 0.8)	0.86
	Sleep duration	108	0.0 (-0.2 to 0.2)	0.91	0.1 (-0.1 to 0.3)	0.37	93	0.1 (-0.7 to 1.0)	0.74	0.3 (-0.4 to 1.1)	0.39	76	-0.1 (-1.0 to 0.8)	0.82	0.1 (-0.9 to 1.1)	0.85
	Model 2															
	Sleep onset	125	-0.2 (-0.4 to 0.0)	0.11	0.1 (-0.1 to 0.3)	0.39	109	-0.5 (-1.2 to 0.2)	0.18	-0.5 (-1.3 to 0.3)	0.23	84	-1.0 (-2.1 to 0.0)	0.060	0.1 (-1.0 to 1.2)	0.85
	Sleep period	125	0.0 (-0.2 to 0.2)	0.92	0.0 (-0.1 to 0.2)	0.81	109	0.1 (-0.6 to 0.9)	0.71	0.0 (-0.6 to 0.6)	0.99	84	0.1 (-1.0 to 1.1)	0.91	-0.3 (-1.2 to 0.5)	0.45
	Sleep duration	108	0.0 (-0.2 to 0.2)	0.99	0.0 (-0.1 to 0.2)	0.62	93	0.2 (-0.6 to 1.1)	0.57	0.2 (-0.6 to 1.1)	0.55	76	-0.1 (-1.0 to 0.8)	0.86	-0.2 (-1.3 to 0.9)	0.74
	Model 3															
	Sleep onset[1]	125	-0.2 (-0.5 to 0.0)	0.072	0.2 (-0.1 to 0.5)	0.15	109	-0.6 (-1.5 to 0.3)	0.18	-0.7 (-1.7 to 0.3)	0.15	**84**	**-1.5 (-2.8 to -0.2)**	**0.025**	-0.2 (-1.6 to 1.2)	0.72
	Sleep onset[2]	**108**	**-0.3 (-0.5 to -0.0)**	**0.044**	0.0 (-0.2 to 0.3)	0.79	93	-0.6 (-1.6 to 0.3)	0.20	-0.5 (-1.4 to 0.4)	0.28	**76**	**-1.5 (-2.9 to 0.0)**	**0.046**	0.0 (-1.3 to 1.2)	0.97
	Sleep period	125	-0.1 (-0.3 to 0.1)	0.41	0.1 (-0.1 to 0.3)	0.31	109	-0.2 (-1.5 to 0.3)	0.69	-0.3 (-1.0 to 0.4)	0.40	84	-0.8 (-2.1 to 0.5)	0.22	-0.6 (-1.6 to 0.4)	0.25
	Sleep duration	108	-0.1 (-0.2 to 0.1)	0.49	0.1 (-0.1 to 0.3)	0.48	93	0.0 (-0.9 to 0.9)	0.99	0.1 (-0.8 to 0.9)	0.88	76	-0.6 (-1.6 to 0.4)	0.23	-0.3 (-1.5 to 0.8)	0.58
36 months	Model 1															
	Sleep onset	96	0.0 (-0.2 to 0.3)	0.71	-0.1 (-0.3 to 0.1)	0.32	96	0.2 (-0.7 to 1.1)	0.63	-0.1 (-0.9 to 0.8)	0.89	67	-0.3 (-1.6 to 0.9)	0.57	-0.6 (-1.6 to 0.5)	0.29
	Sleep period	96	0.0 (-0.3 to 0.3)	0.98	0.0 (-0.2 to 0.2)	0.91	96	-0.5 (-1.6 to 0.5)	0.31	-0.3 (-1.1 to 0.5)	0.40	67	-0.2 (-1.6 to 1.2)	0.82	-0.1 (-1.1 to 0.9)	0.83
	Sleep duration	90	-0.1 (-0.3 to 0.1)	0.36	0.0 (-0.2 to 0.2)	0.98	90	-0.5 (-1.4 to 0.4)	0.26	-0.1 (-0.9 to 0.7)	0.81	62	-0.1 (-1.6 to 1.3)	0.88	0.3 (-0.8 to 1.5)	0.54
	Model 2															
	Sleep onset	96	0.0 (-0.2 to 0.3)	0.87	-0.1 (-0.3 to 0.1)	0.38	96	0.1 (-0.9 to 1.0)	0.90	0.0 (-1.0 to 0.9)	0.92	67	-0.3 (-1.6 to 1.0)	0.68	-0.9 (-2.1 to 0.2)	0.12
	Sleep period	96	0.1 (-0.2 to 0.4)	0.66	0.0 (-0.2 to 0.2)	0.81	96	-0.4 (-1.5 to 0.8)	0.51	-0.2 (-1.1 to 0.6)	0.54	67	-0.1 (-1.6 to 1.3)	0.86	0.1 (-1.0 to 1.1)	0.89
	Sleep duration	90	-0.1 (-0.3 to 0.2)	0.55	0.1 (-0.2 to 0.3)	0.55	90	-0.4 (-1.3 to 0.5)	0.37	0.1 (-0.8 to 1.0)	0.81	62	-0.2 (-1.7 to 1.3)	0.79	0.5 (-0.8 to 1.8)	0.44
	Model 3															
	Sleep onset[1]	96	0.0 (-0.2 to 0.3)	0.79	-0.1 (-0.4 to 0.1)	0.33	96	0.0 (-1.0 to 1.0)	0.98	-0.2 (-1.3 to 0.9)	0.71	67	-0.3 (-1.6 to 1.0)	0.67	-1.3 (-2.8 to 0.1)	0.074
	Sleep onset[2]	90	0.0 (-0.3 to 0.3)	0.98	0.0 (-0.3 to 0.2)	0.72	90	0.0 (-1.0 to 1.0)	0.98	0.1 (-0.9 to 1.1)	0.83	62	-0.4 (-1.9 to 1.2)	0.65	-1.4 (-3.1 to 0.2)	0.085
	Sleep period	96	0.1 (-0.2 to 0.4)	0.63	0.0 (-0.3 to 0.2)	0.76	96	-0.4 (-1.6 to 0.8)	0.52	-0.3 (-1.3 to 0.6)	0.48	67	-0.2 (-1.6 to 1.3)	0.83	-0.6 (-2.0 to 0.7)	0.33
	Sleep duration	90	-0.1 (-0.3 to 0.2)	0.55	0.0 (-0.2 to 0.3)	0.68	90	-0.4 (-1.4 to 0.5)	0.37	0.1 (-0.8 to 1.1)	0.76	62	-0.3 (-1.8 to 1.3)	0.74	-0.4 (-2.0 to 1.3)	0.67

Results are beta-coefficients (95% confidence intervals) and represent the difference in outcomes for every one hour later to sleep, one hour longer sleep period or duration, one hour later to sleep on weekends than weekdays, or one hour longer sleep period or duration on weekends than weekdays, as appropriate. Model 1 was adjusted for sex, age, socioeconomic status, maternal pregnancy age, parity, gestational age, birthweight, maternal body mass index, season of measurement, and napping frequency (models for weekday to weekend differences were further adjusted for the average weekday value of the exposure variable). Model 2 was further adjusted for unhealthy snacking, fruit and vegetable intake, and TV viewing (models for weekday to weekend sleep differences were adjusted for average weekday TV viewing and weekday to weekend differences in TV viewing). Model 3 further adjusted the sleep period and duration for sleep onset, and vice versa (models for weekday to weekend differences were mutually adjusted for average weekday and weekday to weekend differences in the sleep period, duration or onset as appropriate). [1]Adjusted for the sleep period; [2]Adjusted for sleep duration. Statistically significant results ($p<0.05$) are highlighted bold. Sensitivity analyses confirmed that adjustment for physical activity (collected at 36 months only) did not confound Model 3 results, but the association between weighted daily average sleep onset with body mass index *z*-score at 18 months was attenuated when adjusted for time of last meal (BMI *z*-score (n=101): -0.2 (-0.5 to 0.2), p=0.28).

For peer review only

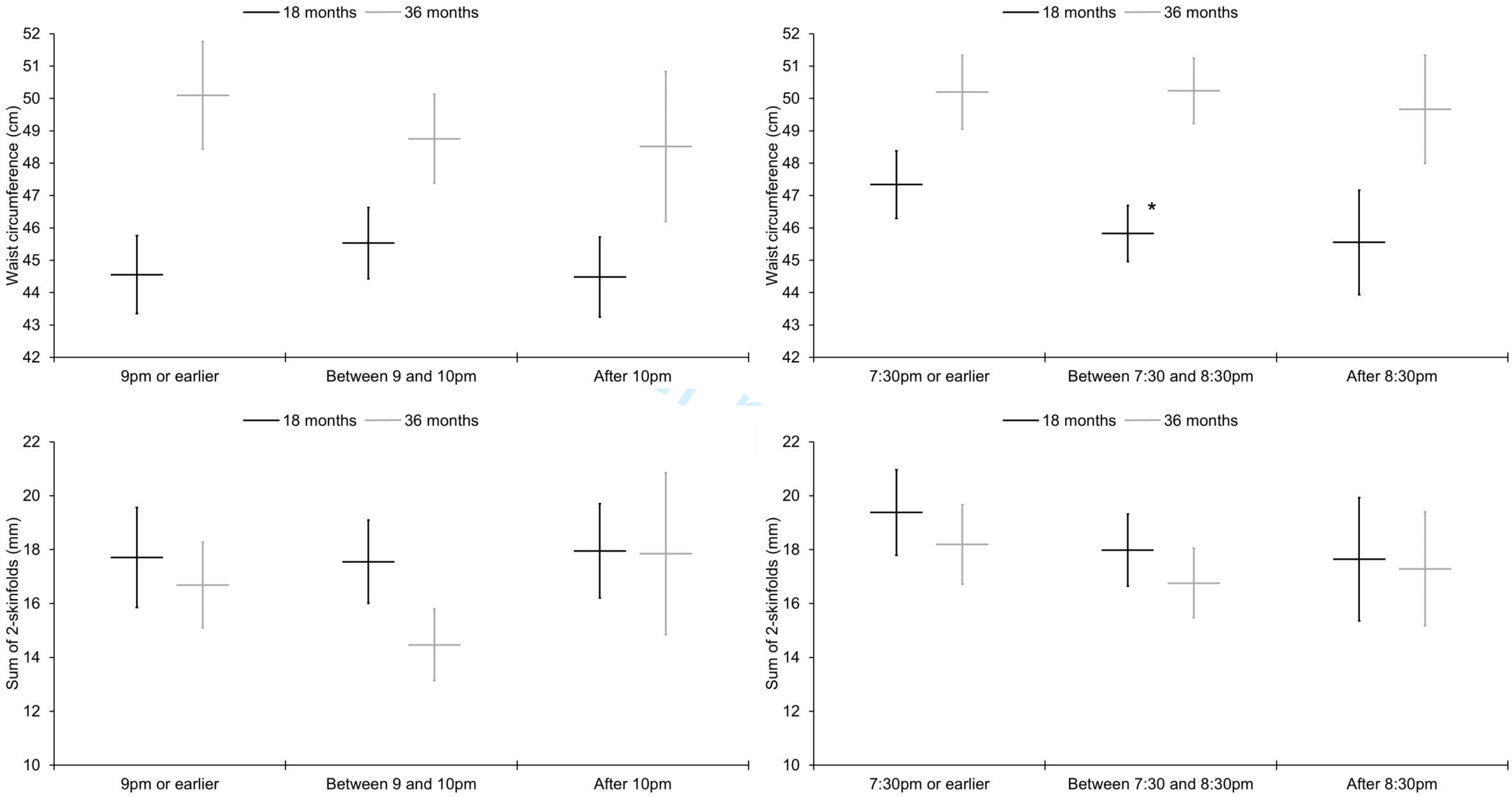
18 months
36 months
Waist circumference (cm)
52
51
50
49
48
47
46
45
44
43
42
9pm or earlier
Between 9 and 10pm
After 10pm
7:30pm or earlier
Between 7:30 and 8:30pm
After 8:30pm
*
Sum of 2-skinfolds (mm)
22
20
18
16
14
12
10

Figure 1

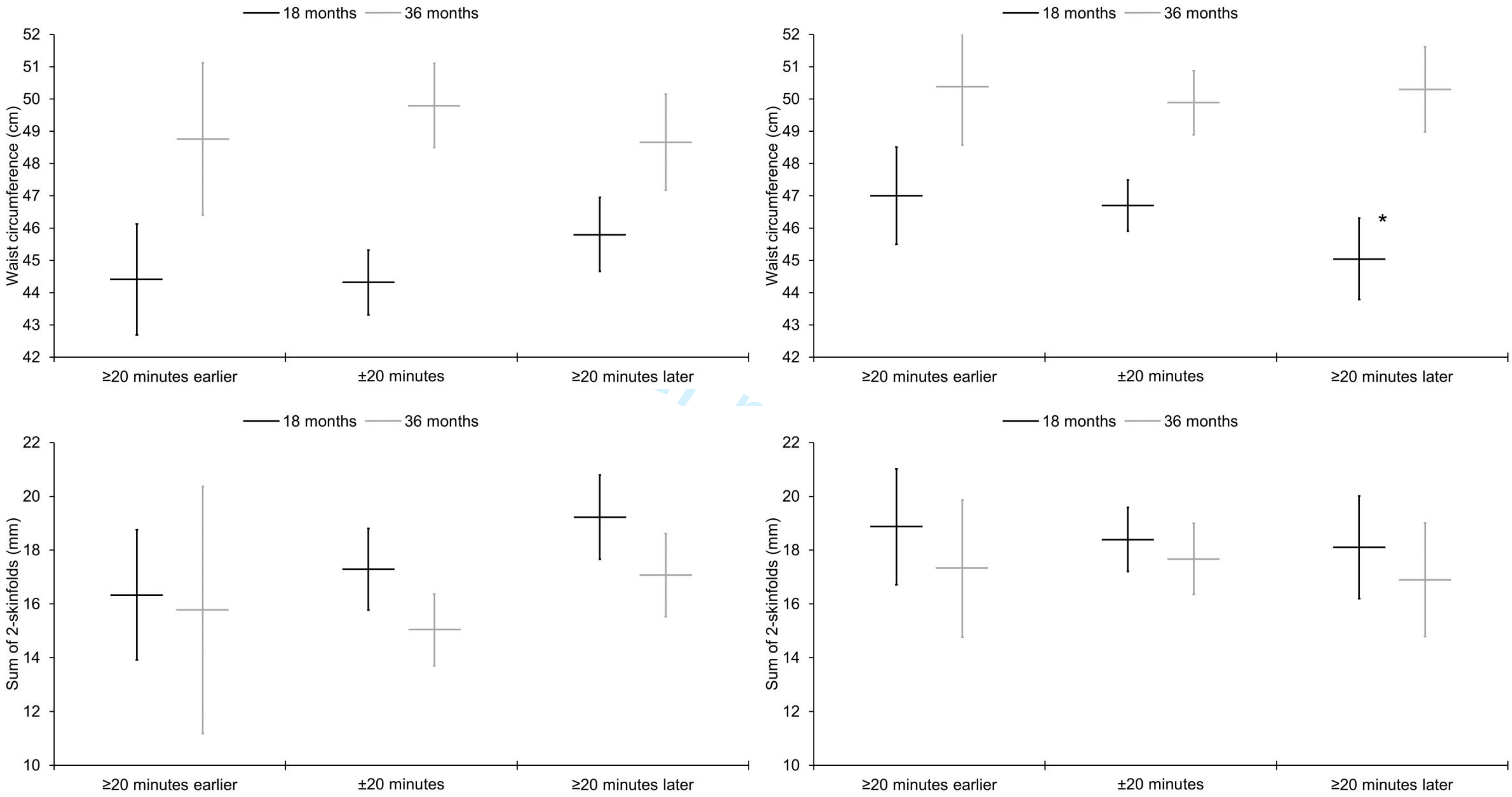
18 months
36 months
Waist circumference (cm)
52
51
50
49
48
47
46
45
44
43
42
≥20 minutes earlier
±20 minutes
≥20 minutes later
*
Sum of 2-skinfolds (mm)
22
20
18
16
14
12
10

Figure 2

Figure 1. Associations of weighted daily average sleep onset categories with waist circumference and sum of 2-skinfolds, stratified by ethnicity and age group

Graphs on the left relate to South Asian children and graphs on the right to White children. Results are estimated marginal means (95% confidence intervals) adjusted for sex, age, socioeconomic status, maternal pregnancy age, parity, gestational age, birthweight, maternal BMI, season of measurement, napping frequency, unhealthy snacking, fruit and vegetable intake, TV viewing, and the sleep period. *Significantly different from the reference category of 7:30pm or earlier (β=-1.5 (-2.9 to -0.1) cm, p=0.035); significant difference persisted when further adjusted for final mealtime (β=-1.7 (-3.2 to -0.1) cm, p=0.036). Results are presented for South Asian children aged 18m (9pm or earlier: waist circumference, n=25; skinfolds, n=19; Between 9 and 10pm: waist circumference, n=26; skinfolds, n=23; After 10pm: waist circumference, n=26; skinfolds, n=22) and 36m (9pm or earlier: waist circumference, n=22; skinfolds, n=16; Between 9 and 10pm: waist circumference, n=28; skinfolds, n=17; After 10pm: waist circumference, n=10; skinfolds, n=5), and White children aged 18m (7:30pm or earlier: waist circumference, n=39; skinfolds, n=30; Between 7:30 and 8:30pm: waist circumference, n=51; skinfolds, n=37; After 8:30pm: waist circumference, n=19; skinfolds, n=17) and 36m (7:30pm or earlier: waist circumference, n=36; skinfolds, n=25; Between 7:30 and 8:30pm: waist circumference, n=42; skinfolds, n=29; After 8:30pm: waist circumference, n=18; skinfolds, n=13).

Figure 2. Associations of weekday to weekend differences in sleep onset with waist circumference and sum of 2-skinfolds, stratified by ethnicity and age group

Graphs on the left relate to South Asian children and graphs on the right to White children. Results are estimated marginal means (95% confidence intervals) adjusted for sex, age, socioeconomic status, maternal pregnancy age, parity, gestational age, birthweight, maternal BMI, season of measurement, napping frequency, average weekday sleep onset, unhealthy snacking, fruit and vegetable intake, average weekday TV viewing, week to weekend differences in TV viewing, average weekday sleep period, and week to weekend differences in the sleep period. *Significantly different from the reference category of ±20 minutes (β=-1.7 (-3.2 to -0.1) cm, p=0.038). Results are presented for South Asian children aged 18m (≥20 minutes earlier: waist circumference, n=16; skinfolds, n=15; ±20 minutes: waist circumference, n=34; skinfolds, n=27; ≥20 minutes later: waist circumference, n=27; skinfolds, n=22) and 36m (≥20 minutes earlier: waist circumference, n=10; skinfolds, n=3; ±20 minutes: waist circumference, n=28; skinfolds, n=21; ≥20 minutes later: waist circumference, n=22; skinfolds, n=14), and White children aged 18m (≥20 minutes earlier: waist circumference, n=20; skinfolds, n=16; ±20 minutes: waist circumference, n=60; skinfolds, n=46; ≥20 minutes later: waist circumference, n=29; skinfolds, n=22) and 36m (≥20 minutes earlier: waist circumference, n=19; skinfolds, n=14; ±20 minutes: waist circumference, n=47; skinfolds, n=35; ≥20 minutes later: waist circumference, n=30; skinfolds, n=18).

For peer review only

STROBE Statement—Checklist of items that should be included in reports of ***cohort studies***

	Item No	Recommendation	Page No
**Title and abstract**	1	(*a*) Indicate the study's design with a commonly used term in the title or the abstract	3
		(*b*) Provide in the abstract an informative and balanced summary of what was done and what was found	3
**Introduction**			
Background/rationale	2	Explain the scientific background and rationale for the investigation being reported	5
Objectives	3	State specific objectives, including any prespecified hypotheses	6
**Methods**			
Study design	4	Present key elements of study design early in the paper	6
Setting	5	Describe the setting, locations, and relevant dates, including periods of recruitment, exposure, follow-up, and data collection	6
Participants	6	(*a*) Give the eligibility criteria, and the sources and methods of selection of participants. Describe methods of follow-up	6
		(*b*) For matched studies, give matching criteria and number of exposed and unexposed	N/A
Variables	7	Clearly define all outcomes, exposures, predictors, potential confounders, and effect modifiers. Give diagnostic criteria, if applicable	6-8
Data sources/ measurement	8*	For each variable of interest, give sources of data and details of methods of assessment (measurement). Describe comparability of assessment methods if there is more than one group	6-8
Bias	9	Describe any efforts to address potential sources of bias	6-8
Study size	10	Explain how the study size was arrived at	6
Quantitative variables	11	Explain how quantitative variables were handled in the analyses. If applicable, describe which groupings were chosen and why	8
Statistical methods	12	(*a*) Describe all statistical methods, including those used to control for confounding	8
		(*b*) Describe any methods used to examine subgroups and interactions	8
		(*c*) Explain how missing data were addressed	6
		(*d*) If applicable, explain how loss to follow-up was addressed	N/A
		(*e*) Describe any sensitivity analyses	8
**Results**			
Participants	13*	(a) Report numbers of individuals at each stage of study—eg numbers potentially eligible, examined for eligibility, confirmed eligible, included in the study, completing follow-up, and analysed	6
		(b) Give reasons for non-participation at each stage	6
		(c) Consider use of a flow diagram	N/A
Descriptive data	14*	(a) Give characteristics of study participants (eg demographic, clinical, social) and information on exposures and potential confounders	9 Tables
		(b) Indicate number of participants with missing data for each variable of interest	Table 1
		(c) Summarise follow-up time (eg, average and total amount)	N/A
Outcome data	15*	Report numbers of outcome events or summary measures over time	N/A

For peer review only - http://bmjopen.bmj.com/site/about/guidelines.xhtml

Main results	16	(*a*) Give unadjusted estimates and, if applicable, confounder-adjusted estimates and their precision (eg, 95% confidence interval). Make clear which confounders were adjusted for and why they were included	9-10 Tables Figures
		(*b*) Report category boundaries when continuous variables were categorized	N/A
		(*c*) If relevant, consider translating estimates of relative risk into absolute risk for a meaningful time period	N/A
Other analyses	17	Report other analyses done—eg analyses of subgroups and interactions, and sensitivity analyses	11
**Discussion**			
Key results	18	Summarise key results with reference to study objectives	10
Limitations	19	Discuss limitations of the study, taking into account sources of potential bias or imprecision. Discuss both direction and magnitude of any potential bias	12-14
Interpretation	20	Give a cautious overall interpretation of results considering objectives, limitations, multiplicity of analyses, results from similar studies, and other relevant evidence	13-14
Generalisability	21	Discuss the generalisability (external validity) of the study results	14
**Other information**			
Funding	22	Give the source of funding and the role of the funders for the present study and, if applicable, for the original study on which the present article is based	2

*Give information separately for exposed and unexposed groups.

Note: An Explanation and Elaboration article discusses each checklist item and gives methodological background and published examples of transparent reporting. The STROBE checklist is best used in conjunction with this article (freely available on the Web sites of PLoS Medicine at http://www.plosmedicine.org/, Annals of Internal Medicine at http://www.annals.org/, and Epidemiology at http://www.epidem.com/). Information on the STROBE Initiative is available at http://www.strobe-statement.org.

For peer review only

BMJ Open

Associations of diarised sleep onset time, period, and duration with total and central adiposity in a biethnic sample of young children: the Born in Bradford observational cohort study

Journal:	*BMJ Open*
Manuscript ID	bmjopen-2020-044769.R1
Article Type:	Original research
Date Submitted by the Author:	27-Jan-2021
Complete List of Authors:	Collings, Paul; University of York, Department of Health Sciences; Bradford Institute for Health Research,<br>Blackwell, Jane; University of York, Department of Health Sciences; Leeds and York Partnership NHS Foundation Trust<br>Pal, Elizabeth; Bradford Trident, Better Start Bradford<br>Ball, Helen; Durham University, Infancy & Sleep Centre & Departmenet of Anthropology<br>Wright, John; Bradford Institute for Health Research,
**Primary Subject Heading**:	Paediatrics
Secondary Subject Heading:	Epidemiology, Public health
Keywords:	SLEEP MEDICINE, EPIDEMIOLOGY, Community child health < PAEDIATRICS, PREVENTIVE MEDICINE, PUBLIC HEALTH

SCHOLARONE™
Manuscripts

I, the Submitting Author has the right to grant and does grant on behalf of all authors of the Work (as defined in the below author licence), an exclusive licence and/or a non-exclusive licence for contributions from authors who are: i) UK Crown employees; ii) where BMJ has agreed a CC-BY licence shall apply, and/or iii) in accordance with the terms applicable for US Federal Government officers or employees acting as part of their official duties; on a worldwide, perpetual, irrevocable, royalty-free basis to BMJ Publishing Group Ltd ("BMJ") its licensees and where the relevant Journal is co-owned by BMJ to the co-owners of the Journal, to publish the Work in this journal and any other BMJ products and to exploit all rights, as set out in our licence.

The Submitting Author accepts and understands that any supply made under these terms is made by BMJ to the Submitting Author unless you are acting as an employee on behalf of your employer or a postgraduate student of an affiliated institution which is paying any applicable article publishing charge ("APC") for Open Access articles. Where the Submitting Author wishes to make the Work available on an Open Access basis (and intends to pay the relevant APC), the terms of reuse of such Open Access shall be governed by a Creative Commons licence – details of these licences and which Creative Commons licence will apply to this Work are set out in our licence referred to above.

Other than as permitted in any relevant BMJ Author's Self Archiving Policies, I confirm this Work has not been accepted for publication elsewhere, is not being considered for publication elsewhere and does not duplicate material already published. I confirm all authors consent to publication of this Work and authorise the granting of this licence.

For peer review only

Title: Associations of diarised sleep onset time, period, and duration with total and central adiposity in a biethnic sample of young children: the Born in Bradford observational cohort study

Authors: Paul J Collings[1,2], Jane E Blackwell[2,3], Elizabeth Pal[4], Helen L Ball[5], John Wright[1]

Affiliations: [1]Bradford Institute for Health Research, Bradford Teaching Hospitals NHS Foundation Trust, Bradford, England; [2]Department of Health Sciences, University of York, England; [3]Leeds and York Partnership NHS Foundation Trust, England; [4]Better Start Bradford, Bradford Trident, Bradford, England; [5]Infancy & Sleep Centre & Department of Anthropology, Department of Anthropology, Durham University, England

Corresponding author email: paul.collings@bthft.nhs.uk

ORCID: Paul J Collings: https://orcid.org/0000-0003-2022-5453; Jane E Blackwell: https://orcid.org/0000-0002-5878-8959; Elizabeth Pal: https://orcid.org/0000-0002-8682-3440; Helen L Ball: https://orcid.org/0000-0003-2255-121X; John Wright: https://orcid.org/0000-0001-9572-7293

Funding: The Born in Bradford study receives core infrastructure funding from the Wellcome Trust (grant number: WT101597MA), a joint grant from the UK Medical Research Council (MRC) and UK Economic and Social Science Research Council (ESRC) (MR/N024397/1), the British Heart Foundation (BHF) (CS/16/4/32482), and the National Institute for Health Research (NIHR) under its Collaboration for Applied Health Research and Care (CLAHRC) for Yorkshire and Humber. This study received delivery support from the NIHR Clinical Research Network. John Wright leads the Healthy Children, Healthy Families Theme of the NIHR CLAHRC in Yorkshire and Humber. Paul J Collings is funded by a BHF Immediate Postdoctoral Basic Science Research Fellowship (FS/17/37/32937). Jane E Blackwell coordinates and Paul J Collings is a member of The White Rose Child & Adolescent Sleep Research Network which is funded by a White Rose Collaboration Grant. The views expressed in this paper are those of the authors and not necessarily those of the MRC, ESRC, BHF, NIHR, and UK Department of Health or National Health Services or of any other funder acknowledged here.

Competing interests: None declared

Contributions: John Wright is Chief Investigator of the Born in Bradford study and setup the cohort; Helen L Ball created the parent-reported sleep diary; Paul J Collings initiated the study, planned and performed the statistical analyses, and wrote the manuscript; all authors including Jane Blackwell and Elizabeth Pal critically revised the manuscript for intellectual content, helped interpret study findings, read and approved the final version and are accountable for the work.

For peer review only - http://bmjopen.bmj.com/site/about/guidelines.xhtml

Data availability: Data generated and analysed for the current study are available from the corresponding author on reasonable request. **Word count**: 4682

For peer review only

Abstract

Objectives: To investigate associations of parent-reported sleep characteristics with adiposity levels in a biethnic sample of young children

Design: Cross-sectional observational study

Setting: The Born in Bradford 1000 study, UK

Participants: Children aged approximately 18 months (m) (*n*=209; 40.2% South Asian; 59.8% White) and 36m (*n*=162; 40.7% South Asian; 59.3% White)

Primary and secondary outcome measures: Children's body mass index (BMI) z-score, sum of 2-skinfolds (triceps and subscapular), and waist circumference. Adjusted regression was used to quantify associations of sleep parameters with adiposity by ethnicity and age group. Results are beta-coefficients (95% confidence intervals) and represent the difference in outcomes for every one hour difference in sleep parameters.

Results: The average sleep onset time was markedly later in South Asian (9:26pm ± 68 minutes) than White children (7:41pm ± 48 minutes). Later sleep onset was associated with lower BMI z-score (-0.3 (-0.5 to -0.0)) and sum of 2-skinfolds (-1.5 (-2.8 to -0.2) mm) in White children aged 18m and higher BMI z-score in South Asian children aged 36m (0.3 (0.0 to 0.5)). Longer sleep duration on weekends than weekdays was associated with higher BMI z-score (0.4 (0.1 to 0.8)) and waist circumference (1.2 (0.3 to 2.2) cm) in South Asian children aged 18m, and in the same group of children later sleep onset on weekends than weekdays was associated with larger sum of 2-skinfolds (1.7 (0.3 to 3.1) mm) and waist circumference (1.8 (0.6 to 2.9) cm). Going to sleep ≥20 minutes later on weekends than weekdays was associated with lower waist circumference in White children aged 18m (-1.7 (-3.2 to -0.1) cm).

Conclusions: Sleep timing is associated with total and central adiposity in young children but associations differ by age group and ethnicity. Sleep onset times and regular sleep schedules may be important for obesity prevention.

For peer review only

Strengths and limitations of this study

- This is the first study to investigate myriad sleep dimensions and how they relate to direct markers of adiposity in young children

- It is also the first study to investigate differences in these associations between UK born South Asian and White children living in a deprived urban setting

- Sleep parameters were mutually adjusted for each other, and the analyses were adjusted for a broad range of covariates including potential mediating factors

- Residual confounding and mediating effects may have persisted because of measurement imprecision in parent-reported covariates, and directions of association between exposures and outcomes cannot be inferred from this cross-sectional study

- The proportion of parents who completed sleep diaries was low, meaning the study sample is susceptible to selection biases, and when stratified by ethnicity and age group some groups were small

For peer review only

Introduction

Many studies have investigated associations of sleep duration or the sleep period (here defined as elapsed time between sleep onset and termination regardless of waking intervals) with adiposity in school-aged children and youth [1,2]. Comparatively few studies have been conducted in infants and toddlers [3,4] despite sleep problems and sleep loss being prevalent in children aged <36 months (m) [5]. Beyond sleep duration, it is beginning to be recognised that sleep is a multidimensional construct of partly overlapping dimensions, and that parameters such as sleep timing may also be influential with regard to adiposity level and obesity risk [6].

Aspects of sleep timing include bedtimes, time of sleep onset, and variability in sleeping pattern such as differences between weekday and weekend sleep schedules. Preliminary evidence indicates that later bedtimes are associated with higher weight status in pre-schoolers [7–10] and primary school-aged children [11], and that later sleep timing on weekends than weekdays is correlated with higher risk of overweight and obesity [12]. Hypothesised mechanisms include circadian dysrhythmia causing hormone-mediated preferences for unhealthy calorific foodstuffs [13,14], the effect of which may be compounded by later sleep onset enabling more time to eat and more opportunity for screen time [15], and lower daily physical activity and higher sedentariness due to delayed waking and fatigue [16]. More studies need to investigate the independent associations of myriad sleep parameters with childhood adiposity and to ascertain the underlying mechanisms of action. A research focus on young children is important, to determine when associations between sleep parameters with adiposity first emerge, and to provide information about how to intervene by identifying modifiable target behaviours [6].

Early childhood is a critical window for obesity prevention because it is a period when rapid weight gain occurs and behavioural patterns are first established [17,18]. Although an increasing number of studies have investigated sleep duration [3,4], few have investigated associations of sleep timing or variability with adiposity in early childhood [19–21], and additional investigation of ethnic minority and socioeconomically impoverished populations is needed. Children of non-White ethnicity and children from deprived backgrounds have unfavourable sleeping patterns and are high-risk for early onset obesity [22,23]. We have previously shown that UK South Asian children who live in a deprived urban setting go to bed much later than White children from the same area [24]. It is important to determine if shifted sleep schedules may in part explain why obesity rates are higher in South Asian than White children in the UK [25], and why South Asian school-aged children have more centrally-stored adiposity which is metabolically harmful [26]. This information could help to

For peer review only

reduce ethnic disparities in disease risk by facilitating behaviour change interventions and policy guidelines that are tailored to the needs of specific ethnic groups.

This study examined independent associations of a broad range of sleep parameters including diarised sleep onset time, period and duration, and weekday to weekend variations in sleep parameters, with total and central adiposity in a young biethnic sample of UK children from a deprived urban setting.

Methods

This study was conducted in Bradford which is the fifth largest local authority in England and one of the most deprived and ethnically diverse cities in the UK [27]. Bradford has an overweight and obesity prevalence of 21.8% in 4 to 5 year old children and 38.4% in 10 to 11 year olds [28]. The Born in Bradford (BiB) 1000 study [29], nested within the larger BiB pregnancy cohort [30], aims to investigate modifiable risk factors for childhood obesity. Pregnant women (*n*=1916) were invited to the BiB 1000 study when they attended routine hospital appointments, 90.6% accepted the invitation (*n*=1735). Consent to medical records access was provided and periodic postnatal assessments were carried out when the women's offspring were approximately 6, 12, 18, 24, and 36m old. Parents who participated when children were aged about 18m (*n*=1228; 70.7% of all BiB 1000 participants) or 36m (*n*=1232; 71%) were asked to complete a sleep diary for their child. At each timepoint diaries were completed for approximately 15% of all BiB 1000 participants (18m: *n*=276; 36m: *n*=262). The diary data were collected between October 2010 and September 2012. This complete-case analysis included only children with sleep diary data, concurrent adiposity measurements and information about potentially important covariates. Maternal ethnicity was used as a proxy for child ethnicity. Children with a mother belonging to an ethnic group other than South Asian (Pakistani / Indian / Bangladeshi) or White (British / other) were excluded from the study due to small numbers. All children were born in the UK (most children of South Asian heritage had Pakistani origin mothers (85%) and most White children had White British mothers (94%)). For brevity we refer to children as being of South Asian or White ethnicity. The final sample included 209 children aged 18m (12% of all BiB 1000 participants) and 162 children aged 36m (9.3%). Ethical approval for all aspects of the study was granted by Bradford Research Ethics Committee (Reference 07/H1302/112) and all mothers provided informed written consent for participation.

Sleep parameters

Parents completed sleep diaries, providing free-text responses about the time their child fell asleep on an evening and woke-up the next morning. The sleep period was calculated as elapsed time between sleep onset and waking the next day. Because the sleep period does

For peer review only

not account for periods of overnight waking which can be common in young children [31] parents also reported their child's overnight sleep duration. Parents completed diaries on two weekdays and a weekend day. Mean weekday values were calculated by averaging data for the two weekdays; thereafter daily averages over the course of a week were calculated using weekday to weekend weighting (in the ratio of 5:2). Differences in sleep parameters between weekdays and weekends were calculated by subtraction (weekend – weekday values).

Adiposity markers

Child weight and height were assessed by trained researchers and BMI (kg/m^2) and *z*-scores were calculated [32]. Waist circumference was measured at the level of the navel. Skinfolds of the left triceps and subscapular were measured to the nearest 0.1mm and summed. Reliability metrics indicated good intra- and inter-observer technical error for measurements [33].

Covariates

Potential confounders and mediators of associations were selected based on previous evidence linking them with sleep and obesity [17,22]. A multidimensional marker of socioeconomic status was synthesised from information collated during interviews with parents (usually mothers) about their education, employment, housing tenure, financial situation, and ownership of goods. For the purposes of this study, from an initial five categories [34], children were classified as belonging to one of three parental socioeconomic status groups: least deprived (least socioeconomically deprived and most educated), moderately deprived (employed and not materially deprived / employed but no access to money) and most deprived (benefits but not materially deprived / most economically deprived). Maternal age was self-reported in pregnancy whereas the number of previous births (used to group mothers as primaparous (pregnant for the first time) or not), child gender, gestational age, and birth weight were extracted from medical records. Maternal height and weight were measured at the 18 and 36m timepoints and were used to calculate maternal BMI. Parents reported whether or not their child napped on sleep diary days, these data were used to create three categories of napping frequency (never: napped zero days; occasionally: napped 1 or 2 days; every day: napped all three days). On each sleep diary reporting day, parents also provided free-text responses about the time their child ate their last meal of the day. Mean weekday values were calculated by averaging data across the two weekdays, and weekday to weekend weighting was used to calculate the average daily time that the child ate their last meal; differences between weekdays and weekends were calculated by subtraction. Infant dietary data were collected using a validated food frequency questionnaire that was modified to include ethnic-specific foodstuffs. Dietary constructs

For peer review only

indicative of unhealthy snacking (frequency of biscuit, crisps, cakes, sweets, chocolate, sugar-sweetened beverage consumption) and fruit and vegetable consumption in the previous 4-12 weeks were derived [24]. Parents reported the number of hours their child watched TV on a typical weekday and weekend day; weighting was used to calculate average daily TV viewing and weekday to weekend differences were calculated [35]. When children were aged 36m a parent-reported Early Years Physical Activity Questionnaire (EY-PAQ) was used to estimate children's physical activity level [36]. Calendar dates of the 18 and 36m assessments were recorded and categorised by season (summer / autumn or spring / winter).

Patient and public involvement (PPI)

The BiB research team regularly convenes a Parent Governors PPI group, BiB participants who are now aged 10 to 12 years can become Young Ambassadors of the study, and BiB runs regular community events and science festivals (https://borninbradford.nhs.uk/news-events/events/). These committees and events help to shape our research by allowing us to learn about the opinions, concerns and ideas of the community. A study author (JEB) recently chaired a PPI event held in conjunction with The Sleep Charity [37]. Attendees were parents of children with sleep difficulties, staff who work in residential settings with children, sleep practitioners and representatives from parent and carer forums. Emerging themes from the session included: 1) there is a lack of information about assisting development of healthy sleep routines in infants, 2) more information and knowledge about the consequences of poor sleep in infants is needed, and 3) early intervention is vital and parents of young children should be offered evidence-based sleep interventions for their child.

Statistics

Correlations between individual sleep parameters and adiposity markers were calculated using Pearson or Spearman methods as appropriate [38]. Adjusted linear regression was used to investigate cross-sectional associations between sleep parameters with adiposity. Model 1 adjusted associations for sex, age, socioeconomic status, maternal pregnancy age, parity, gestational age, birthweight, maternal BMI, season of measurement, and napping frequency. Model 2 further adjusted for potential confounding or mediating factors, including unhealthy snacking, fruit and vegetable intake, and TV viewing. Model 3 mutually adjusted sleep period and duration variables for sleep onset time, and vice versa. All analyses were performed stratified by ethnic group and age group. Interaction effects between sleep period and duration with sleep onset time were examined by introducing multiplicative terms to models (eg. sleep onset*duration). Nonlinearity of associations was also examined by adding quadratic terms for sleep parameters. There was some of evidence for nonlinear

For peer review only - http://bmjopen.bmj.com/site/about/guidelines.xhtml

associations between average daily sleep onset and weekday to weekend differences in sleep onset with waist circumference and sum of 2-skinfolds, hence sleep onset variables were additionally trichotomised and adjusted linear regression was used to estimate group marginal means (and 95% confidence intervals (CI)) for these outcomes. Data for the sum of 2-skinfolds were slightly skewed, but because results were consistent regardless of whether the data were log-transformed or not, results based on the untransformed data are presented. Sensitivity analyses further adjusted associations for the time of last meal and physical activity (not applicable to 18m models when physical activity was not assessed), potentially important covariates but missing data; models with waist circumference or sum of 2-skinfolds as dependent variables were further adjusted for height. Analyses were performed with Stata/SE 16.1 software (StataCorp, College Station, TX). Variance inflation factors were calculated to highlight potential multicollinearity and $p<0.05$ was deemed to indicate statistically significant associations, but all results are interpreted with emphasis on the range of plausible values of associations as shown by confidence intervals [39].

Results

Sample characteristics

Participant details are shown in **Table 1**. Child ages ranged from 16.4 to 22.8m and 35.5 to 39.5m at each respective timepoint. The average daily sleep onset time was consistently after 9pm in South Asian children and before 8pm in White children. Sleep onset time was inversely correlated with the sleep period (South Asian children aged 18m: -0.52; South Asian children aged 36m: -0.38; White children aged 18m: -0.52; White children aged 36m: -0.37) and to a lesser extent sleep duration (South Asian children aged 18m: -0.42; South Asian children aged 36m: -0.10; White children aged 18m: -0.38; White children aged 36m: -0.27). Correlations between the sleep period and sleep duration were weaker in South Asian than White children (South Asian children aged 18m: 0.72; South Asian children aged 36m: 0.66; White children aged 18m: 0.81; White children aged 36m: 0.83). Correlations between adiposity markers differed by ethnicity and age group, but consistently BMI z-score was more strongly correlated with waist circumference (South Asian children aged 18m: 0.69; South Asian children aged 36m: 0.71; White children aged 18m: 0.61; White children aged 36m: 0.79) than the sum of 2-skinfolds (South Asian children aged 18m: 0.54; South Asian children aged 36m: 0.28; White children aged 18m: 0.44; White children aged 36m: 0.53); correlations between waist circumference and the sum of 2-skinfolds were weakest (South Asian children aged 18m: 0.47; South Asian children aged 36m: 0.19; White children aged 18m: 0.26; White children aged 36m: 0.47).

South Asian children

For peer review only

Table 2 shows that in South Asian children aged 18m there were no significant associations between average daily sleep parameters with adiposity. In children aged 36m, there was some evidence in model 3 that later daily sleep onset was associated with higher BMI *z*-score; the association attenuated when further adjusted for final mealtime (β=0.2 (95% CI: -0.2 to 0.6), *p*=0.34). There were no other significant associations for average daily sleep parameters. **Figure 1** presents the group marginal means for waist circumference and the sum of 2-skinfolds stratified by sleep onset categories; there were no group differences. With regard to weekday to weekend differences, Table 2 shows that longer sleep duration on weekends than weekdays was consistently associated with higher adiposity in South Asian children aged 18m, and in the same group of children, later sleep onset on weekends than weekdays was associated with larger waist circumference and sum of 2-skinfolds. In South Asian children aged 36m, later sleep onset on weekends than weekdays was associated with larger sum of 2-skinfolds in model 2, but the association attenuated when further adjusted for the sleep period. **Figure 2** shows the adjusted marginal means for waist circumference and sum of 2-skinfolds stratified by three categories of weekday to weekend differences in sleep onset. There was some evidence that compared to children with consistent sleep onset times (±20 minutes of each other), children aged 18m who went to sleep ≥20 minutes later on weekends than weekdays had larger waist circumferences (1.5 (-0.1 to 3.0) cm, *p*=0.060) and sum of 2-skinfolds (1.9 (-0.2 to 4.1) mm, *p*=0.078). Similarly, there was some indication that aged 36m, children who went to sleep ≥20 minutes later on weekends than weekdays had larger sum of 2-skinfolds (2.0 (-0.2 to 4.3) mm, *p*=0.074).

White children

Table 3 shows that for White children aged 36m there were no significant associations, although there was some indication that later sleep onset on weekends than weekdays was associated with smaller sum of 2-skinfolds. In children aged 18m, independent of all covariables including sleep period and duration, later daily sleep onset was associated with smaller sum of 2-skinfolds and lower BMI *z*-score (though further adjustment for final mealtime attenuated the latter association (-0.2 (-0.5 to 0.2), *p*=0.28). Figure 1 illustrates that at age 18m, compared to children with a sleep onset time of 7:30pm or earlier, children who began to sleep between 7:30 and 8:30pm had smaller waist circumferences (-1.5 (-2.9 to -0.1) cm, *p*=0.035). Figure 2 highlights that children aged 18m who went to sleep ≥20 minutes later on weekends than weekdays had smaller waist circumferences than children with consistent sleep onset times (-1.7 (-3.2 to -0.1) cm, *p*=0.038).

Sensitivity analyses

Further adjustment for physical activity in models that included children aged 36m, and adjustment for height when waist circumference and the sum of 2-skinfolds were modelled

For peer review only - http://bmjopen.bmj.com/site/about/guidelines.xhtml

as dependent variables, did not influence any of the reported associations. There was no evidence for interaction effects between sleep onset with the sleep period or sleep duration. Variance inflation factors were within acceptable limits.

Discussion

This study investigated associations of diarised sleep onset time, period and duration, and weekday to weekend differences in sleep parameters, with total and central adiposity in young children aged 18 and 36m. We discovered age- and ethnicity-specific associations for sleep onset times, which we in part attribute to markedly different sleep schedules between ethnic groups; South Asian children went to sleep on average nearly two hours later than White children. Later average sleep onset and later sleep timing on weekends than weekdays were associated with higher adiposity in South Asian children, and conversely with lower adiposity in White children, particularly those aged 18m. Longer sleep durations on weekends than weekdays were associated with higher adiposity in South Asian children aged 18m.

Numerous systematic reviews have summarised the evidence for associations of sleep duration or the sleep period with adiposity in children and adolescents [1,2]. Of note, one review meta-analysed the results of six prospective studies totalling 14,264 children aged <36m. There was moderate heterogeneity in results, but each additional hour of sleep was associated with a negative change in BMI z-score over follow-up [3]. Limitations of the existing evidence include a preponderance of studies that have relied solely on BMI or BMI z-score as proxies for total adiposity. Studies have also considered few potential confounding or mediating factors and focussed solely on sleep duration. Sleep is a multidimensional construct and it is important to unravel the independent relations of specific dimensions with adiposity. In a previous BiB 1000 study we analysed repeated questionnaire data collected at four timepoints (12, 18, 24 and 36m) to quantify associations between parent reported sleep duration with measured adiposity in 1338 UK South Asian and White children. Longer sleep duration predicted lower total and central adiposity but only in South Asian children [24]. Here, in a sub-sample of the same cohort, we were able to scrutinise and mutually adjust for myriad sleep parameters collected over the course of three days via parent-completed sleep diaries. Independent of sleep length, the average sleep onset time and variability in sleep behaviours between weekdays and weekends emerged as the key modifiable dimensions that predicted adiposity in South Asian and White children.

A recent review concluded that sleep timing, in particular later bedtimes, are associated with higher weight status in primary school-aged children [11]. Similarly, the few studies conducted thus far in 4 to 5 year olds indicate that later bedtimes, and most markedly

For peer review only - http://bmjopen.bmj.com/site/about/guidelines.xhtml

bedtimes after 9pm, are associated with higher BMI z-score and obesity risk [7–9]. A particular study found that an association of short sleep with higher BMI z-score was only evident in children who went to bed after 9pm [10]. Just two studies have been performed in younger children. Zhang et al. found no differences in internally derived BMI z-scores between early and late bedtime groups (differentiated by using a crude median split at about 8pm) in a sample of children with ages ranging from 12 to 26m [19]. Roy et al. found that later bedtimes in children aged 36m were associated with higher odds of obesity, but the association attenuated when adjusted for sleep duration [20]. To our knowledge, this is the first study to show that independent of the sleep period, later sleep onset is associated with higher BMI z-score in South Asian children aged 36m. The association attenuated when adjusted for final mealtime, which provides some indication that later sleep onset may elevate obesity risk via later evening meals and higher caloric intake later in the day [40]. In line with this hypothesis, animal models and clinical trials have shown that early and time-restricted feeding has numerous physiological benefits, including lower adiposity [41,42].

With regard to sleep variability, a pooled analysis of five studies conducted in school-aged children and youth indicated that later sleep timing on weekends than weekdays was correlated with higher risk of overweight or obesity, albeit with a small effect size [12]. In toddlers, a recent study found that inconsistent sleep onset times over the course of a week predicted higher BMI *z*-scores, independent of device-measured physical activity and diet quality, and that inconsistent sleep indirectly explained an association between household poverty with higher BMI z-score [21]. We uniquely observed that later sleep timing on weekends than weekdays was associated with higher adiposity in South Asian children aged 18m and larger sum of 2-skinfolds in South Asian children aged 36m, although the association in older children only approached statistical significance. The associations in children aged 18m were independent of potential confounding or mediating factors including TV viewing, unhealthy snacking, fruit and vegetable intake, and the final mealtime. It may be that these are not important mediators in very young children who are less autonomous and for whom a large part of their diet and screen time is governed by parents and carers. That said, we cannot exclude residual confounding or mediating effects as all covariate data were parent-reported and are subject to random error and bias. Longer sleep duration on weekends than weekdays was associated with higher adiposity in South Asian children aged 18m. This suggests that consistent sleep durations are important.

We observed that later daily sleep onset was associated with lower BMI z-score in White children aged 18m. This observation may appear counterintuitive, but Roy et al. similarly reported that independent of overnight sleep duration, each one hour later to bed was associated with -0.08 lower BMI z-score in 878 children aged 24 months, although their

For peer review only

confidence interval narrowly crossed the null of no association (95% CI: -0.17 to 0.01) [20]. Following adjustment for final mealtime our result for BMI z-score also attenuated to the null, but inverse associations with more direct adiposity indicators remained, including smaller sum of 2-skinfolds and waist circumference as a function of later sleep onset. These unexpected results may partly be explained by early sleep onset obstructing time during early-to-mid evenings that could otherwise be spent physically active. Time-stamped accelerometer studies show that daily physical activity patterns are bimodal in 18m olds. Physical activity levels first peak in the morning, slump after midday due to feeding and napping (81% of our White children aged 18m napped every day), after which there is a second larger peak that begins around 4pm and tails-off by 8pm [43,44]. In our sample of White children aged 18m, the earliest sleep onset times were around 6pm and more than one-third (36.8%) were asleep by 7:30pm. It is conceivable that these children may have missed a substantial part of the evening peak in physical activity, thus contributing to higher adiposity [33,45]. Our related observation in the same group of children, that going to sleep ≥20 minutes later on weekends than weekdays was associated with lower waist circumference, may be explained by later sleep onset at the weekend enabling more opportunity to be physically active [46].

Although White children aged 36m exhibited a similar distribution of sleep onset times compared to their younger White peers, there were few comparable associations, except some indication in children aged 36m that later sleep onset on weekends than weekdays was associated with lower adiposity (an association with the sum of 2-skinfolds approached statistical significance). The apparent age-related differences may be explained by daily physical activity patterns following a different trajectory in slightly older children who tend not to nap (just 5% of White children aged 36m napped every day; 46% napped occasionally; 49% did not nap). Instead of a naptime-related slump after midday which is followed by an evening peak, physical activity levels tend to be relatively constant throughout the afternoon, before progressively declining in the evening and most notably from 7pm onwards [47]. Hence, in many White children aged 36m, there may have been no peak in evening physical activity for early sleep to obstruct. In contrast to our results, Roy et al. did find a significant association between later bedtimes with lower BMI z-score in children aged approximately 36m [20]. That study was performed in children living in America, Australia and New Zealand which may account for the discrepancy. In those countries frequent daytime naps persist in older children partly because of designated naptimes in childcare [48–50]. Hence many children aged 3 to 4 years continue to exhibit a bimodal physical activity pattern, including an early-to-mid evening activity peak, which early sleep might obstruct [51].

For peer review only

Sleep recommendations for children have historically focussed on duration rather than other sleep dimensions [52]. Our results highlight that contemporary sleep guidelines should further acknowledge the importance of sleep timing and regular sleep schedules. Any such recommendations should also be considerate of ethnic and cultural differences in sleeping patterns, including what may and may not be feasible and acceptable to different population subgroups. Our results for White children aged 18m, some of whom were already asleep just after 6pm, indicate that going to sleep too early could be detrimental from an energy-balance and obesity risk perspective. Sleep onset times were much later in South Asian children who may benefit from an earlier evening meal, earlier sleep onset time, and consistent sleep schedules across week and weekend days. It is difficult to decipher if any such changes would translate to clinically meaningful differences in adiposity levels because young children tend to be healthy. However, more than half (54%) of South Asian children aged 18m went to sleep later on weekends than weekdays, on average by 32 (± 23) minutes. According to our point estimates this could be associated with up to 1cm larger waist circumference. This is unlikely trivial, particularly considering that any beneficial influence on adiposity levels this early in childhood may be important, as it is a critical period when rapid weight gain occurs and sustained obesity can develop [18].

This study uniquely investigated associations of diarised sleep parameters with myriad adiposity markers in a biethnic sample of young children from a deprived urban setting, a high-risk group for sleep problems and obesity [22,23]. Sleep diaries enabled information about numerous dimensions of sleep to be collated but unfortunately they incur participant burden. This likely contributed to only a minority of BiB 1000 parents choosing to complete sleep diaries for their child, the consequences of which are at least twofold. First, our study sample is subject to selection biases. Compared to the full BiB pregnancy cohort, which was broadly representative of the obstetric population in Bradford at the time of recruitment, the proportion of families who were most deprived was lower in this study (28% versus 45%) [34]. This hinders generalisability, although it is still reassuring that there was variability in parental socioeconomic status. Second, stratifying by ethnicity and age group meant that some of our analyses were based on relatively few observations and low statistical power. This latter issue was compounded by missing data for specific items, such as sleep duration (which was only reported if parents considered they knew how long their child had slept overnight) and the sum of 2-skinfolds. The results should therefore be considered exploratory and require replication in larger samples. It is advantageous that we investigated more direct adiposity markers than weight-for-height proxies which tend to underestimate fatness in South Asians [53], and that in addition to skinfolds we investigated waist circumferences, as South Asian children eventually develop more centrally-stored adiposity

For peer review only - http://bmjopen.bmj.com/site/about/guidelines.xhtml

which is metabolically deleterious [26]. Another strength is that each of our analyses were adjusted for a broad range of covariates, including mutual adjustment for sleep parameters to tease apart independent associations of distinct sleep dimensions with adiposity. Unfortunately, directions of association cannot be inferred from this cross-sectional study, it is possible that adiposity could influence sleeping habits and vice versa [24]. Longitudinal studies or trials are needed to determine directions of association. Future studies may achieve a better trade-off between participant retention and data resolution if they use sensor-based technologies rather than diaries [54] although a combination of methods may prove best to capture habitual sleep parameters [55].

Conclusions

Sleep onset times appear to be independently associated with levels of total and central adiposity in young South Asian and White children. Suitable sleep times and consistent sleep schedules across week and weekend days may be important modifiable determinants of early childhood obesity.

For peer review only

References

1 Matricciani L, Paquet C, Galland B, *et al.* Children's sleep and health: A meta-review. *Sleep Med Rev* 2019;**46**:136–50. doi:10.1016/j.smrv.2019.04.011

2 Sun J, Wang M, Yang L, *et al.* Sleep duration and cardiovascular risk factors in children and adolescents: A systematic review. *Sleep Med Rev* 2020;**53**:101338. doi:10.1016/j.smrv.2020.101338

3 Miller MA, Kruisbrink M, Wallace J, *et al.* Sleep duration and incidence of obesity in infants, children, and adolescents: a systematic review and meta-analysis of prospective studies. *Sleep* 2018;**41**:1–19. doi:10.1093/sleep/zsy018

4 Harskamp-van Ginkel MW, Chinapaw MJM, Harmsen IA, *et al.* Sleep during Infancy and Associations with Childhood Body Composition: A Systematic Review and Narrative Synthesis. *Child Obes* 2020;**16**:94–116. doi:10.1089/chi.2019.0123

5 Ophoff D, Slaats MA, Boudewyns A, *et al.* Sleep disorders during childhood: a practical review. *Eur J Pediatr* 2018;**177**:641–8. doi:10.1007/s00431-018-3116-z

6 Miller AL, Lumeng JC, LeBourgeois MK. Sleep patterns and obesity in childhood. *Curr Opin Endocrinol diabetes Obes* 2015;**22 SRC**-:41–7.

7 Scharf RJ, Deboer MD. Sleep timing and longitudinal weight gain in 4- and 5-year-old children. *Pediatr Obes* 2015;**10**:141–8. doi:10.1111/ijpo.229

8 Anderson SE, Andridge R, Whitaker RC. Bedtime in Preschool-Aged Children and Risk for Adolescent Obesity. *J Pediatr* 2016;**176**:17–22. doi:10.1016/j.jpeds.2016.06.005

9 Simon SL, Goetz AR, Meier M, *et al.* Sleep duration and bedtime in preschool-age children with obesity: Relation to BMI and diet following a weight management intervention. *Pediatr Obes* 2019;**14**:1–9. doi:10.1111/ijpo.12555

10 Miller AL, Kaciroti N, Lebourgeois MK, *et al.* Sleep timing moderates the concurrent sleep duration-body mass index association in low-income preschool-age children. *Acad Pediatr* 2014;**14**:207–13. doi:10.1016/j.acap.2013.12.003

11 Morrissey B, Taveras E, Allender S, *et al.* Sleep and obesity among children: A systematic review of multiple sleep dimensions. *Pediatr Obes* 2020;**15**:1–21. doi:10.1111/ijpo.12619

12 Sun W, Ling J, Zhu X, *et al.* Associations of weekday-to-weekend sleep differences with academic performance and health-related outcomes in school-age children and youths. *Sleep Med Rev* 2019;**46**:27–53. doi:10.1016/j.smrv.2019.04.003

13 Petrov ME, Vander Wyst KB, Whisner CM, *et al.* Relationship of Sleep Duration and Regularity with Dietary Intake Among Preschool-Aged Children with Obesity from Low-Income Families. *J Dev Behav Pediatr* 2017;**38**:120–8. doi:10.1097/DBP.0000000000000369

For peer review only

14 Jansen EC, Peterson KE, Lumeng JC, *et al.* Associations between Sleep and Dietary Patterns among Low-Income Children Attending Preschool. *J Acad Nutr Diet* 2019;**119**:1176–87. doi:10.1016/j.jand.2019.01.008
15 Moorman JD, Morgan P, Adams TL. The Implications of Screen Media Use for the Sleep Behavior of Children Ages 0–5: a Systematic Review of the Literature. *Curr Sleep Med Reports* 2019;**5**:164–72. doi:10.1007/s40675-019-00151-0
16 Olds TS, Maher CA, Matricciani L. Sleep Duration or Bedtime? Exploring the Relationship between Sleep Habits and Weight Status and Activity Patterns. *Sleep* 2011;**34**:1299–307. doi:10.5665/sleep.1266
17 Woo Baidal JA, Locks LM, Cheng ER, *et al.* Risk Factors for Childhood Obesity in the First 1,000 Days. *Am J Prev Med* 2016;**50**:761–79. doi:10.1016/j.amepre.2015.11.012
18 Geserick M, Vogel M, Gausche R, *et al.* Acceleration of BMI in Early Childhood and Risk of Sustained Obesity. *N Engl J Med* 2018;**379**:1303–12. doi:10.1056/NEJMoa1803527
19 Zhang Z, Pereira JR, Sousa-Sá E, *et al.* The cross-sectional and prospective associations between sleep characteristics and adiposity in toddlers: Results from the GET UP! Study. *Pediatr Obes* 2019;**14**. doi:10.1111/ijpo.12557
20 Roy M, Haszard JJ, Savage JS, *et al.* Bedtime, body mass index and obesity risk in preschool-aged children. *Pediatr Obes* 2020;:ijpo.12650. doi:10.1111/ijpo.12650
21 Covington L, Armstrong B, Trude ACB, *et al.* Longitudinal Associations Among Diet Quality, Physical Activity and Sleep Onset Consistency With Body Mass Index z -Score Among Toddlers in Low-income Families. *Ann Behav Med* Published Online First: 16 November 2020. doi:10.1093/abm/kaaa100
22 Blair PS, Humphreys JS, Gringras P, *et al.* Childhood Sleep Duration and Associated Demographic Characteristics in an English Cohort. *Sleep* 2012;**35**:353–60. doi:10.5665/sleep.1694
23 Lu Y, Pearce A, Li L. Distinct patterns of socio-economic disparities in child-to-adolescent BMI trajectories across UK ethnic groups: A prospective longitudinal study. *Pediatr Obes* 2020;**15**:1–9. doi:10.1111/ijpo.12598
24 Collings PJ, Ball HL, Santorelli G, *et al.* Sleep Duration and Adiposity in Early Childhood: Evidence for Bidirectional Associations from the Born in Bradford Study. *Sleep* 2017;**40**. doi:10.1093/sleep/zsw054
25 Hudda MT, Nightingale CM, Donin AS, *et al.* Patterns of childhood body mass index (BMI), overweight and obesity in South Asian and black participants in the English National child measurement programme: effect of applying BMI adjustments standardising for ethnic differences in BMI-body fatness asso. *Int J Obes* 2018;**42**:662–70. doi:10.1038/ijo.2017.272

For peer review only

26 Wolf RM, Nagpal M, Magge SN. Diabetes and cardiometabolic risk in south Asian youth: A review. *Pediatr Diabetes* 2020;**Online**:1–15. doi:10.1111/pedi.13078

27 City of Bradford Metropolitan District Council. Understanding Bradford District. 2020.https://ubd.bradford.gov.uk/ (accessed 22 Apr 2020).

28 Local Government Association (LGA) Research. National Child Measurement Programme data for Bradford. https://lginform.local.gov.uk/reports/view/lga-research/latest-data-from-the-national-child-measurement-programme-ncmp?mod-area=E08000032&mod-group=AllRegions_England&mod-type=namedComparisonGroup (accessed 23 Apr 2020).

29 Bryant M, Santorelli G, Fairley L, *et al.* Design and characteristics of a new birth cohort, to study the early origins and ethnic variation of childhood obesity: the BiB1000 study. *Longit Life Course Stud* 2013;**4**:119–35. doi:10.14301/llcs.v4i2.221

30 Wright J, Small N, Raynor P, *et al.* Cohort profile: The born in bradford multi-ethnic family cohort study. *Int J Epidemiol* 2013;**42**:978–91. doi:10.1093/ije/dys112

31 Sadeh A, Mindell JA, Luedtke K, *et al.* Sleep and sleep ecology in the first 3 years: A web-based study. *J Sleep Res* Published Online First: 2009. doi:10.1111/j.1365-2869.2008.00699.x

32 Cole TJ, Freeman JV, Preece MA. Body mass index reference curves for the UK, 1990. *Arch Dis Child* 1995;**73**:25–9. doi:10.1136/adc.73.1.25

33 Collings PJ, Brage S, Bingham DD, *et al.* Physical Activity, Sedentary Time, and Fatness in a Biethnic Sample of Young Children. *Med Sci Sport Exerc* 2017;**49**:930–8. doi:10.1249/MSS.0000000000001180

34 Fairley L, Cabieses B, Small N, *et al.* Using latent class analysis to develop a model of the relationship between socioeconomic position and ethnicity: cross-sectional analyses from a multi-ethnic birth cohort study. *BMC Public Health* 2014;**14**:835. doi:10.1186/1471-2458-14-835

35 Collings PJ, Kelly B, West J, *et al.* Associations of TV Viewing Duration, Meals and Snacks Eaten When Watching TV, and a TV in the Bedroom with Child Adiposity. *Obesity* 2018;**26**:1619–28. doi:10.1002/oby.22288

36 Bingham D, Collings P, Clemes S, *et al.* Reliability and Validity of the Early Years Physical Activity Questionnaire (EY-PAQ). *Sports* 2016;**4**:30. doi:10.3390/sports4020030

37 The Sleep Charity. https://thesleepcharity.org.uk/ (accessed 28 Aug 2020).

38 de Winter JCF, Gosling SD, Potter J. Comparing the Pearson and Spearman correlation coefficients across distributions and sample sizes: A tutorial using simulations and empirical data. *Psychol Methods* 2016;**21**:273–90. doi:10.1037/met0000079

For peer review only

39 Amrhein V, Greenland S, McShane B. Scientists rise up against statistical significance. *Nature* 2019;**567**:305–7. doi:10.1038/d41586-019-00857-9

40 Almoosawi S, Vingeliene S, Karagounis LG, *et al.* Chrono-nutrition: A review of current evidence from observational studies on global trends in time-of-day of energy intake and its association with obesity. *Proc Nutr Soc* 2016;**75**:487–500. doi:10.1017/S0029665116000306

41 Sutton EF, Beyl R, Early KS, *et al.* Early Time-Restricted Feeding Improves Insulin Sensitivity, Blood Pressure, and Oxidative Stress Even without Weight Loss in Men with Prediabetes. *Cell Metab* 2018;**27**:1212-1221.e3. doi:10.1016/j.cmet.2018.04.010

42 de Cabo R, Mattson MP. Effects of Intermittent Fasting on Health, Aging, and Disease. *N Engl J Med* 2019;**381**:2541–51. doi:10.1056/NEJMra1905136

43 Hnatiuk J, Ridgers ND, Salmon J, *et al.* Physical activity levels and patterns of 19-month-old children. *Med Sci Sports Exerc* Published Online First: 2012. doi:10.1249/MSS.0b013e31825825c4

44 Prioreschi A, Brage S, Hesketh KD, *et al.* Describing objectively measured physical activity levels, patterns, and correlates in a cross sectional sample of infants and toddlers from South Africa. *Int J Behav Nutr Phys Act* 2017;**14**:176. doi:10.1186/s12966-017-0633-5

45 Collings PJ, Brage S, Ridgway CL, *et al.* Physical activity intensity, sedentary time, and body composition in preschoolers. *Am J Clin Nutr* Published Online First: 2013. doi:10.3945/ajcn.112.045088

46 Collings PJ, Dogra SA, Costa S, *et al.* Objectively-measured sedentary time and physical activity in a bi-ethnic sample of young children: variation by socio-demographic, temporal and perinatal factors. *BMC Public Health* 2020;**20**:109. doi:10.1186/s12889-019-8132-z

47 Hesketh KR, McMinn AM, Ekelund U, *et al.* Objectively measured physical activity in four-year-old British children: a cross-sectional analysis of activity patterns segmented across the day. *Int J Behav Nutr Phys Act* 2014;**11**:1. doi:10.1186/1479-5868-11-1

48 Ward TM, Gay C, Anders TF, *et al.* Sleep and Napping Patterns in 3-to-5-year old Children Attending Full-Day Childcare Centers. *J Pediatr Psychol* 2008;**33**:666–72. doi:10.1093/jpepsy/jsm102

49 Staton SL, Smith SS, Hurst C, *et al.* Mandatory Nap Times and Group Napping Patterns in Child Care: An Observational Study. *Behav Sleep Med* 2017;**15**:129–43. doi:10.1080/15402002.2015.1120199

50 Staton S, Rankin PS, Harding M, *et al.* Many naps, one nap, none: A systematic review and meta-analysis of napping patterns in children 0–12 years. *Sleep Med Rev* 2020;**50**:101247. doi:10.1016/j.smrv.2019.101247

For peer review only

51 Dias K, White J, Jago R, *et al.* International Comparison of the Levels and Potential Correlates of Objectively Measured Sedentary Time and Physical Activity among Three-to-Four-Year-Old Children. *Int J Environ Res Public Health* 2019;**16**:1929. doi:10.3390/ijerph16111929

52 Matricciani LA, Olds TS, Blunden S, *et al.* Never Enough Sleep: A Brief History of Sleep Recommendations for Children. *Pediatrics* 2012;**129**:548–56. doi:10.1542/peds.2011-2039

53 Hudda MT, Nightingale CM, Donin AS, *et al.* Body mass index adjustments to increase the validity of body fatness assessment in UK Black African and South Asian children. *Int J Obes* 2017;**41**:1048–55. doi:10.1038/ijo.2017.75

54 Schoch SF, Kurth S, Werner H. Actigraphy in sleep research with infants and young children: Current practices and future benefits of standardized reporting. *J Sleep Res* 2020;:1–15. doi:10.1111/jsr.13134

55 Collings PJ, Wijndaele K, Corder K, *et al.* Prospective associations between sedentary time, sleep duration and adiposity in adolescents. *Sleep Med* 2015;**16**:717–22. doi:10.1016/j.sleep.2015.02.532

For peer review only

Figure 1. Associations of sleep onset categories with waist circumference and sum of 2-skinfolds, stratified by ethnicity and age group

Graphs on the left relate to South Asian children and graphs on the right to White children. Results are estimated marginal means (95% confidence intervals) adjusted for sex, age, socioeconomic status, maternal pregnancy age, parity, gestational age, birthweight, maternal BMI, season of measurement, napping frequency, unhealthy snacking, fruit and vegetable intake, TV viewing, and the sleep period. *Significantly different from the reference category of 7:30pm or earlier (β=-1.5 (-2.9 to -0.1) cm, *p*=0.035); significant difference persisted when further adjusted for final mealtime (*n*=103: β=-1.7 (-3.2 to -0.1) cm, *p*=0.036). [†]Some evidence for a difference compared to the reference category: (β=-1.8 (-3.8 to 0.3) cm, p=0.088). Results are presented for South Asian children aged 18m (9pm or earlier: waist circumference, *n*=25; skinfolds, *n*=19; Between 9 and 10pm: waist circumference, *n*=26; skinfolds, *n*=23; After 10pm: waist circumference, *n*=26; skinfolds, *n*=22) and 36m (9pm or earlier: waist circumference, *n*=22; skinfolds, *n*=16; Between 9 and 10pm: waist circumference, *n*=28; skinfolds, *n*=17; After 10pm: waist circumference, *n*=10; skinfolds, *n*=5), and White children aged 18m (7:30pm or earlier: waist circumference, *n*=39; skinfolds, *n*=30; Between 7:30 and 8:30pm: waist circumference, *n*=51; skinfolds, *n*=37; After 8:30pm: waist circumference, *n*=19; skinfolds, *n*=17) and 36m (7:30pm or earlier: waist circumference, *n*=36; skinfolds, *n*=25; Between 7:30 and 8:30pm: waist circumference, *n*=42; skinfolds, *n*=29; After 8:30pm: waist circumference, *n*=18; skinfolds, *n*=13).

Figure 2. Associations of weekday to weekend differences in sleep onset with waist circumference and sum of 2-skinfolds, stratified by ethnicity and age group

Graphs on the left relate to South Asian children and graphs on the right to White children. Results are estimated marginal means (95% confidence intervals) adjusted for sex, age, socioeconomic status, maternal pregnancy age, parity, gestational age, birthweight, maternal BMI, season of measurement, napping frequency, average weekday sleep onset, unhealthy snacking, fruit and vegetable intake, average weekday TV viewing, week to weekend differences in TV viewing, average weekday sleep period, and week to weekend differences in the sleep period. *Significantly different from the reference category of ±20 minutes (β=-1.7 (-3.2 to -0.1) cm, *p*=0.038). [†]Some evidence for a difference compared to the reference category: (β=1.5 (-0.1 to 3.0) cm, *p*=0.060). [‡]Some evidence for a difference compared to the reference category: (β=1.9 (-0.2 to 4.1) mm, *p*=0.078). [¶]Some evidence for a difference compared to the reference category: (β=2.0 (-0.2 to 4.3) mm, *p*=0.074). Results are presented for South Asian children aged 18m (≥20 minutes earlier: waist circumference, *n*=16; skinfolds, *n*=15; ±20 minutes: waist circumference, *n*=34; skinfolds, *n*=27; ≥20 minutes later: waist circumference, *n*=27; skinfolds, *n*=22) and 36m (≥20 minutes earlier: waist

For peer review only

circumference, *n*=10; skinfolds, *n*=3; ±20 minutes: waist circumference, *n*=28; skinfolds, *n*=21; ≥20 minutes later: waist circumference, *n*=22; skinfolds, *n*=14), and White children aged 18m (≥20 minutes earlier: waist circumference, *n*=20; skinfolds, *n*=16; ±20 minutes: waist circumference, *n*=60; skinfolds, *n*=46; ≥20 minutes later: waist circumference, *n*=29; skinfolds, *n*=22) and 36m (≥20 minutes earlier: waist circumference, *n*=19; skinfolds, *n*=14; ±20 minutes: waist circumference, *n*=47; skinfolds, *n*=35; ≥20 minutes later: waist circumference, *n*=30; skinfolds, *n*=18).

For peer review only

Table 1. Description of study participants

	South Asian		White	
	18 months (*n*=84)	36 months (*n*=66)	18 months (*n*=125)	36 months (*n*=96)
Sex (*n* (%) boys)	43 (51.2)	32 (48.5)	58 (46.4)	38 (39.6)
Age (months)	18.1 (0.7)	36.7 (1.1)	18.3 (1.1)	36.5 (0.5)
Parental socioeconomic status (*n* (%))				
Least deprived	22 (26.2)	15 (22.7)	32 (25.6)	30 (31.3)
Moderately deprived	29 (34.5)	20 (30.3)	70 (56.0)	53 (55.2)
Most deprived	33 (39.3)	31 (47.0)	23 (18.4)	13 (13.5)
Maternal ethnicity (*n* (%) Pakistani origin)	73 (86.9)	55 (83.3)	-	-
Maternal ethnicity (*n* (%) White British)	-	-	119 (95.2)	89 (92.7)
Maternal pregnancy age (y)	27.6 (7.3)	30.6 (7.6)	29.1 (10.3)	28.8 (8.2)
Parity (*n* (%) primaparous)	30 (35.7)	21 (31.8)	65 (52.0)	60 (62.5)
Birthweight (g)	3156 ± 465	3066 ± 552	3400 ± 543	3358 ± 563
Gestational age (weeks)	39.6 ± 1.6	39.4 ± 1.6	39.8 ± 1.5	39.6 ± 1.9
Season (*n* (%) summer / autumn)	71 (84.5)	22 (33.3)	108 (86.4)	18 (18.8)
Unhealthy snacks (*n*/week)	10 (10)	17 (23)	9 (8)	11 (12)
Fruit & vegetables (portions/day)	6 ± 2	6 ± 3	5 ± 2	5 ± 3
Physical activity (h/day)	-	2.8 ± 1.5	-	2.7 (2.0)
Maternal BMI (kg/m^2)	26.4 (6.3)	27.2 (6.6)	25.1 (8.0)	25.8 (7.0)
Napping frequency (*n* (%))				
Never	0 (0)	24 (36.4)	4 (3.2)	47 (49.0)
Occasionally	7 (8.3)	26 (39.4)	20 (16.0)	44 (45.8)
Everyday	77 (91.7)	16 (24.2)	101 (80.8)	5 (5.2)
Weighted daily average				
TV viewing (h/day)	1.0 (1.6)	1.5 (1.7)	0.6 (1.0)	1.5 (1.1)
Time of last meal (pm)	7:23 ± 66	7:20 ± 59	5:28 ± 51	5:32 ± 43
Sleep onset (pm)	9:36 ± 69	9:12 ± 63	7:50 ± 50	7:54 ± 44
Sleep offset (am)	8:21 ± 63	8:23 ± 63	7:11 ± 47	7:12 ± 50
Sleep period (h/night)	10.8 ± 1.0	11.2 ± 0.8	11.4 ± 0.8	11.3 ± 0.7
Sleep duration (h/night)	10.5 ± 1.0	11.0 ± 0.9	11.1 ± 1.1	11.3 ± 0.8
Weekday to weekend difference				
TV viewing (h/day)	-0.1 ± 0.6	0.0 ± 0.6	0.0 ± 0.6	0.0 ± 0.9
Time of last meal (h)	0.2 ± 0.7	0.2 ± 0.7	0.1 ± 0.8	0.2 ± 0.7
Sleep onset (h)	0.1 ± 0.6	0.3 ± 0.8	0.1 ± 0.9	0.1 ± 0.8
Sleep offset (h)	0.2 ± 0.9	0.3 ± 0.8	0.3 ± 0.8	0.2 ± 0.9
Sleep period (h/night)	0.1 ± 1.0	0.0 ± 0.8	0.2 ± 1.2	0.1 ± 1.0
Sleep duration (h/night)	0.0 ± 0.9	-0.1 ± 1.0	0.0 ± 1.0	0.0 ± 1.1
BMI (kg/m^2)	16.0 ± 1.3	15.8 ± 1.2	16.7 ± 1.2	16.4 ± 1.2
BMI *z*-score	-0.8 ± 1.1	-0.2 ± 1.0	-0.2 ± 0.9	0.3 ± 0.8
Waist circumference (cm)	44.9 ± 2.8	49.2 ± 3.1	46.3 ± 3.0	50.1 ± 3.1
Sum of 2-skinfolds (mm)	17.1 (3.8)	15.8 (3.4)	17.4 (4.3)	17.2 (4.8)

Normally distributed continuous variables are described as mean ± sd. Time of last meal, sleep onset and offset are described as clock times ± sd in minutes. Non-normally distributed continuous variables are described as median (IQR). Positive weekday to weekend differences indicate more TV viewing or sleep at the weekend than on weekdays, or later clock times at the weekend. There were missing data: Physical activity available for *n*=60 South Asian children at 36 months and *n*=89 White children at 36 months; Time of last meal available for *n*=71 South Asian children at 18 months, *n*=59 South Asian children at 36 months, *n*=119 White children at 18 months, *n*=91 White children at 36 months; Sleep duration available for *n*=63 South Asian children at 18 months, *n*=45 South Asian children at 36 months, *n*=108 White children at 18 months, *n*=90 White children at 36 months; Waist circumference available for *n*=77 South Asian children at 18 months, *n*=60 South Asian children at 36 months, *n*=109 White children at 18 months, *n*=96 White children at 36 months; Sum of 2-skinfolds available for *n*=64 South Asian children at 18 months, *n*=38 South Asian children at 36 months, *n*=84 White children at 18 months, *n*=67 White children at 36 months. BMI, Body mass index

For peer review only

Table 2. Associations of sleep onset, period and duration with adiposity in South Asian children, stratified by age group

		**Body mass index *z*-score**					**Waist circumference (cm)**					**Sum of 2-skinfolds (mm)**				
			Weighted daily average		Weekday to weekend difference			Weighted daily average		Weekday to weekend difference			Weighted daily average		Weekday to weekend difference	
		n	β (95% CI)	*p*	β (95% CI)	*p*	*n*	β (95% CI)	*p*	β (95% CI)	*p*	*n*	β (95% CI)	*p*	β (95% CI)	*p*
18 months	Model 1															
	Sleep onset	84	-0.1 (-0.3 to 0.2)	0.66	0.1 (-0.4 to 0.5)	0.76	77	-0.4 (-1.0 to 0.2)	0.24	0.8 (-0.3 to 1.8)	0.17	64	-0.2 (-1.1 to 0.7)	0.68	1.3 (-0.1 to 2.8)	0.070
	Sleep period	84	0.2 (-0.1 to 0.4)	0.16	0.0 (-0.3 to 0.3)	0.84	77	0.3 (-0.3 to 1.0)	0.30	0.0 (-0.7 to 0.8)	0.91	64	0.2 (-0.7 to 1.0)	0.70	-0.3 (-1.4 to 0.8)	0.56
	Sleep duration	63	0.2 (-0.2 to 0.5)	0.29	0.3 (-0.1 to 0.6)	0.17	58	0.3 (-0.6 to 1.2)	0.54	0.6 (-0.4 to 1.7)	0.21	49	0.5 (-0.7 to 1.6)	0.41	0.8 (-0.6 to 2.2)	0.26
	Model 2															
	Sleep onset	84	0.0 (-0.2 to 0.2)	0.93	0.0 (-0.4 to 0.4)	0.93	77	-0.3 (-0.9 to 0.4)	0.41	0.7 (-0.4 to 1.8)	0.21	64	-0.1 (-0.9 to 0.8)	0.90	**1.6 (0.1 to 3.1)**	**0.036**
	Sleep period	84	0.2 (-0.1 to 0.4)	0.22	0.1 (-0.2 to 0.4)	0.59	77	0.3 (-0.4 to 1.0)	0.39	0.3 (-0.5 to 1.1)	0.47	64	0.1 (-0.8 to 1.0)	0.89	-0.2 (-1.4 to 0.9)	0.72
	Sleep duration	63	0.2 (-0.1 to 0.5)	0.27	0.3 (-0.0 to 0.7)	0.066	58	0.3 (-0.6 to 1.2)	0.55	0.9 (-0.1 to 1.9)	0.075	49	0.3 (-0.9 to 1.5)	0.61	0.9 (-0.6 to 2.3)	0.23
	Model 3															
	Sleep onset[1]	84	0.1 (-0.2 to 0.4)	0.40	0.0 (-0.5 to 0.5)	0.99	77	-0.2 (-0.9 to 0.6)	0.65	1.2 (-0.1 to 2.4)	0.076	64	0.0 (-1.1 to 1.1)	0.96	**1.8 (0.1 to 3.5)**	**0.034**
	Sleep onset[2]	63	0.2 (-0.1 to 0.5)	0.23	0.4 (-0.1 to 0.8)	0.12	58	0.2 (-0.6 to 1.1)	0.58	**1.8 (0.6 to 2.9)**	**0.004**	49	0.0 (-1.0 to 1.1)	0.92	**1.7 (0.3 to 3.1)**	**0.022**
	Sleep period	84	0.2 (-0.1 to 0.5)	0.14	0.1 (-0.2 to 0.4)	0.63	77	0.2 (-0.6 to 1.0)	0.60	0.7 (-0.2 to 1.5)	0.14	64	0.0 (-1.1 to 1.1)	0.93	0.3 (-0.9 to 1.5)	0.61
	Sleep duration	63	0.3 (-0.1 to 0.6)	0.14	**0.4 (0.1 to 0.8)**	**0.024**	58	0.3 (-0.6 to 1.3)	0.46	**1.2 (0.3 to 2.2)**	**0.012**	49	0.3 (-0.9 to 1.5)	0.61	1.4 (-0.0 to 2.9)	0.056
36 months	Model 1															
	Sleep onset	66	0.1 (-0.1 to 0.4)	0.17	-0.3 (-0.6 to 0.0)	0.085	60	-0.1 (-1.0 to 0.7)	0.74	**-1.2 (-2.4 to -0.0)**	**0.048**	38	-0.3 (-1.3 to 0.7)	0.52	0.9 (-0.3 to 2.1)	0.14
	Sleep period	66	0.1 (-0.2 to 0.4)	0.50	0.2 (-0.1 to 0.5)	0.14	60	-0.4 (-1.7 to 0.8)	0.48	**1.1 (0.0 to 2.3)**	**0.047**	38	0.0 (-1.2 to 1.3)	0.96	-0.3 (-1.8 to 1.1)	0.64
	Sleep duration	45	0.2 (-0.1 to 0.5)	0.15	-0.0 (-0.4 to 0.3)	0.82	42	0.5 (-0.6 to 1.5)	0.35	0.3 (-1.0 to 1.6)	0.63	26	0.2 (-0.9 to 1.2)	0.71	-0.1 (-1.8 to 1.6)	0.92
	Model 2															
	Sleep onset	66	0.2 (-0.1 to 0.4)	0.13	-0.2 (-0.6 to 0.1)	0.15	60	0.1 (-0.8 to 1.0)	0.86	-1.0 (-2.2 to 0.2)	0.11	38	-0.2 (-1.4 to 1.0)	0.75	**1.3 (0.1 to 2.5)**	**0.033**
	Sleep period	66	0.1 (-0.2 to 0.5)	0.42	0.2 (-0.1 to 0.5)	0.14	60	-0.4 (-1.7 to 0.9)	0.55	**1.2 (0.1 to 2.3)**	**0.032**	38	0.1 (-1.2 to 1.4)	0.84	-1.1 (-2.7 to 0.4)	0.15
	Sleep duration	45	0.2 (-0.1 to 0.5)	0.27	0.1 (-0.3 to 0.5)	0.67	42	0.3 (-0.8 to 1.4)	0.55	0.5 (-0.8 to 1.9)	0.44	26	-0.0 (-1.1 to 1.0)	0.97	-0.8 (-3.1 to 1.5)	0.43
	Model 3															
	Sleep onset[1]	**66**	**0.3 (0.0 to 0.5)**	**0.042**	-0.2 (-0.6 to 0.2)	0.27	60	0.0 (-0.9 to 0.9)	0.97	-0.2 (-1.7 to 1.3)	0.80	38	-0.2 (-1.5 to 1.1)	0.79	1.2 (-0.3 to 2.8)	0.12
	Sleep onset[2]	45	0.1 (-0.1 to 0.4)	0.34	-0.2 (-0.8 to 0.3)	0.43	42	-0.3 (-1.2 to 0.6)	0.53	0.0 (-1.9 to 2.0)	0.95	26	-0.8 (-2.2 to 0.7)	0.25	0.4 (-2.3 to 3.1)	0.72
	Sleep period	66	0.3 (-0.1 to 0.6)	0.12	0.1 (-0.2 to 0.5)	0.42	60	-0.4 (-1.8 to 1.0)	0.57	1.1 (-0.3 to 2.5)	0.11	38	0.1 (-1.3 to 1.5)	0.89	-0.4 (-2.2 to 1.4)	0.68
	Sleep duration	45	0.2 (-0.1 to 0.5)	0.23	0.0 (-0.4 to 0.4)	0.85	42	0.3 (-0.8 to 1.4)	0.55	0.5 (-0.9 to 2.0)	0.45	26	-0.1 (-1.2 to 0.9)	0.80	-0.2 (-3.4 to 2.9)	0.85

Results are beta-coefficients (95% confidence intervals) and represent the difference in outcomes for every one hour later to sleep, one hour longer sleep period or duration, one hour later to sleep on weekends than weekdays, or one hour longer sleep period or duration on weekends than weekdays. Model 1 was adjusted for sex, age, socioeconomic status, maternal pregnancy age, parity, gestational age, birthweight, maternal body mass index, season of measurement, and napping frequency (models for weekday to weekend differences were further adjusted for the average weekday value of the exposure variable). Model 2 was further adjusted for unhealthy snacking, fruit and vegetable intake, and TV viewing (models for weekday to weekend sleep differences were adjusted for average weekday TV viewing and weekday to weekend differences in TV viewing). Model 3 further adjusted sleep period and duration variables for sleep onset, and vice versa (models for weekday to weekend differences were mutually adjusted for average weekday and weekday to weekend differences in the sleep period, duration or onset as appropriate). [1]Adjusted for the sleep period; [2]Adjusted for sleep duration. Statistically significant results ($p<0.05$) are highlighted bold. Sensitivity analyses confirmed that adjustment for physical activity (collected at 36 months only) did not confound Model 3 results, but the association between weighted daily average sleep onset with body mass index *z*-score at 36 months was attenuated when adjusted for time of last meal (body mass index *z*-score (n=59): 0.2 (-0.2 to 0.6), p=0.34).

Table 3. Associations of sleep onset, period and duration with adiposity in White children, stratified by age group

		Body mass index *z*-score					Waist circumference (cm)					Sum of 2-skinfolds (mm)				
			Weighted daily average		Weekday to weekend difference			Weighted daily average		Weekday to weekend difference			Weighted daily average		Weekday to weekend difference	
		n	β (95% CI)	*p*	β (95% CI)	*p*	*n*	β (95% CI)	*p*	β (95% CI)	*p*	*n*	β (95% CI)	*p*	β (95% CI)	*p*
18 months	Model 1															
	Sleep onset	125	-0.2 (-0.4 to 0.0)	0.086	0.0 (-0.2 to 0.2)	0.70	109	-0.5 (-1.2 to 0.2)	0.18	-0.6 (-1.4 to 0.2)	0.17	84	-1.0 (-2.1 to 0.0)	0.061	-0.1 (-1.2 to 1.0)	0.88
	Sleep period	125	0.0 (-0.2 to 0.2)	0.83	0.1 (-0.1 to 0.2)	0.44	109	0.2 (-0.6 to 0.9)	0.64	0.1 (-0.5 to 0.7)	0.70	84	0.1 (-1.0 to 1.2)	0.82	-0.1 (-0.9 to 0.8)	0.86
	Sleep duration	108	0.0 (-0.2 to 0.2)	0.91	0.1 (-0.1 to 0.3)	0.37	93	0.1 (-0.7 to 1.0)	0.74	0.3 (-0.4 to 1.1)	0.39	76	-0.1 (-1.0 to 0.8)	0.82	0.1 (-0.9 to 1.1)	0.85
	Model 2															
	Sleep onset	125	-0.2 (-0.4 to 0.0)	0.11	0.1 (-0.1 to 0.3)	0.39	109	-0.5 (-1.2 to 0.2)	0.18	-0.5 (-1.3 to 0.3)	0.23	84	-1.0 (-2.1 to 0.0)	0.060	0.1 (-1.0 to 1.2)	0.85
	Sleep period	125	0.0 (-0.2 to 0.2)	0.92	0.0 (-0.1 to 0.2)	0.81	109	0.1 (-0.6 to 0.9)	0.71	0.0 (-0.6 to 0.6)	0.99	84	0.1 (-1.0 to 1.1)	0.91	-0.3 (-1.2 to 0.5)	0.45
	Sleep duration	108	0.0 (-0.2 to 0.2)	0.99	0.0 (-0.1 to 0.2)	0.62	93	0.2 (-0.6 to 1.1)	0.57	0.2 (-0.6 to 1.1)	0.55	76	-0.1 (-1.0 to 0.8)	0.86	-0.2 (-1.3 to 0.9)	0.74
	Model 3															
	Sleep onset[1]	125	-0.2 (-0.5 to 0.0)	0.072	0.2 (-0.1 to 0.4)	0.15	109	-0.6 (-1.5 to 0.3)	0.18	-0.7 (-1.7 to 0.3)	0.15	**84**	**-1.5 (-2.8 to -0.2)**	**0.025**	-0.2 (-1.6 to 1.2)	0.72
	Sleep onset[2]	**108**	**-0.3 (-0.5 to -0.0)**	**0.044**	0.0 (-0.2 to 0.3)	0.79	93	-0.6 (-1.6 to 0.3)	0.20	-0.5 (-1.4 to 0.4)	0.28	**76**	**-1.5 (-2.9 to -0.0)**	**0.046**	-0.0 (-1.3 to 1.2)	0.97
	Sleep period	125	-0.1 (-0.3 to 0.1)	0.41	0.1 (-0.1 to 0.3)	0.31	109	-0.2 (-1.0 to 0.7)	0.69	-0.3 (-1.0 to 0.4)	0.40	84	-0.8 (-2.1 to 0.5)	0.22	-0.6 (-1.6 to 0.4)	0.25
	Sleep duration	108	-0.1 (-0.2 to 0.1)	0.49	0.1 (-0.1 to 0.3)	0.48	93	0.0 (-0.9 to 0.9)	0.99	0.1 (-0.8 to 0.9)	0.88	76	-0.6 (-1.6 to 0.4)	0.23	-0.3 (-1.5 to 0.8)	0.58
36 months	Model 1															
	Sleep onset	96	0.0 (-0.2 to 0.3)	0.71	-0.1 (-0.3 to 0.1)	0.32	96	0.2 (-0.7 to 1.1)	0.63	-0.1 (-0.9 to 0.8)	0.89	67	-0.3 (-1.6 to 0.9)	0.57	-0.6 (-1.6 to 0.5)	0.29
	Sleep period	96	0.0 (-0.3 to 0.3)	0.98	-0.0 (-0.2 to 0.2)	0.91	96	-0.5 (-1.6 to 0.5)	0.31	-0.3 (-1.1 to 0.5)	0.40	67	-0.2 (-1.6 to 1.2)	0.82	-0.1 (-1.1 to 0.9)	0.83
	Sleep duration	90	-0.1 (-0.3 to 0.1)	0.36	-0.0 (-0.2 to 0.2)	0.98	90	-0.5 (-1.4 to 0.4)	0.26	-0.1 (-0.9 to 0.7)	0.81	62	-0.1 (-1.6 to 1.3)	0.88	0.3 (-0.8 to 1.5)	0.54
	Model 2															
	Sleep onset	96	0.0 (-0.2 to 0.3)	0.87	-0.1 (-0.4 to 0.1)	0.38	96	0.1 (-0.9 to 1.0)	0.90	-0.0 (-1.0 to 0.9)	0.92	67	-0.3 (-1.6 to 1.0)	0.68	-0.9 (-2.1 to 0.2)	0.12
	Sleep period	96	0.1 (-0.2 to 0.4)	0.66	0.0 (-0.2 to 0.2)	0.81	96	-0.4 (-1.5 to 0.8)	0.51	-0.3 (-1.1 to 0.6)	0.54	67	-0.1 (-1.6 to 1.3)	0.86	0.1 (-1.0 to 1.1)	0.89
	Sleep duration	90	-0.1 (-0.3 to 0.2)	0.55	0.1 (-0.2 to 0.3)	0.55	90	-0.4 (-1.3 to 0.5)	0.37	0.1 (-0.8 to 1.0)	0.81	62	-0.2 (-1.7 to 1.3)	0.79	0.5 (-0.8 to 1.8)	0.44
	Model 3															
	Sleep onset[1]	96	0.0 (-0.2 to 0.3)	0.79	-0.1 (-0.4 to 0.1)	0.33	96	0.0 (-1.0 to 1.0)	0.98	-0.2 (-1.3 to 0.9)	0.71	67	-0.3 (-1.6 to 1.0)	0.67	-1.3 (-2.8 to 0.1)	0.074
	Sleep onset[2]	90	0.0 (-0.3 to 0.3)	0.98	-0.0 (-0.3 to 0.2)	0.72	90	0.0 (-1.0 to 1.0)	0.98	0.1 (-0.9 to 1.1)	0.83	62	-0.4 (-1.9 to 1.2)	0.65	-1.4 (-3.1 to 0.2)	0.085
	Sleep period	96	0.1 (-0.2 to 0.4)	0.63	-0.0 (-0.3 to 0.2)	0.76	96	-0.4 (-1.6 to 0.8)	0.52	-0.3 (-1.3 to 0.6)	0.48	67	-0.2 (-1.6 to 1.3)	0.83	-0.6 (-2.0 to 0.7)	0.33
	Sleep duration	90	-0.1 (-0.3 to 0.2)	0.55	0.1 (-0.2 to 0.3)	0.68	90	-0.4 (-1.4 to 0.5)	0.37	0.1 (-0.8 to 1.1)	0.76	62	-0.3 (-1.8 to 1.3)	0.74	-0.3 (-2.0 to 1.3)	0.67

Results are beta-coefficients (95% confidence intervals) and represent the difference in outcomes for every one hour later to sleep, one hour longer sleep period or duration, one hour later to sleep on weekends than weekdays, or one hour longer sleep period or duration on weekends than weekdays. Model 1 was adjusted for sex, age, socioeconomic status, maternal pregnancy age, parity, gestational age, birthweight, maternal body mass index, season of measurement, and napping frequency (models for weekday to weekend differences were further adjusted for the average weekday value of the exposure variable). Model 2 was further adjusted for unhealthy snacking, fruit and vegetable intake, and TV viewing (models for weekday to weekend sleep differences were adjusted for average weekday TV viewing and weekday to weekend differences in TV viewing). Model 3 further adjusted sleep period and duration variables for sleep onset, and vice versa (models for weekday to weekend differences were mutually adjusted for average weekday and weekday to weekend differences in the sleep period, duration or onset as appropriate). [1]Adjusted for the sleep period; [2]Adjusted for sleep duration. Statistically significant results ($p<0.05$) are highlighted bold. Sensitivity analyses confirmed that adjustment for physical activity (collected at 36 months only) did not confound Model 3 results, but the association between weighted daily average sleep onset with body mass index *z*-score at 18 months was attenuated when adjusted for time of last meal (BMI *z*-score (n=101): -0.2 (-0.5 to 0.2), p=0.28).

For peer review only

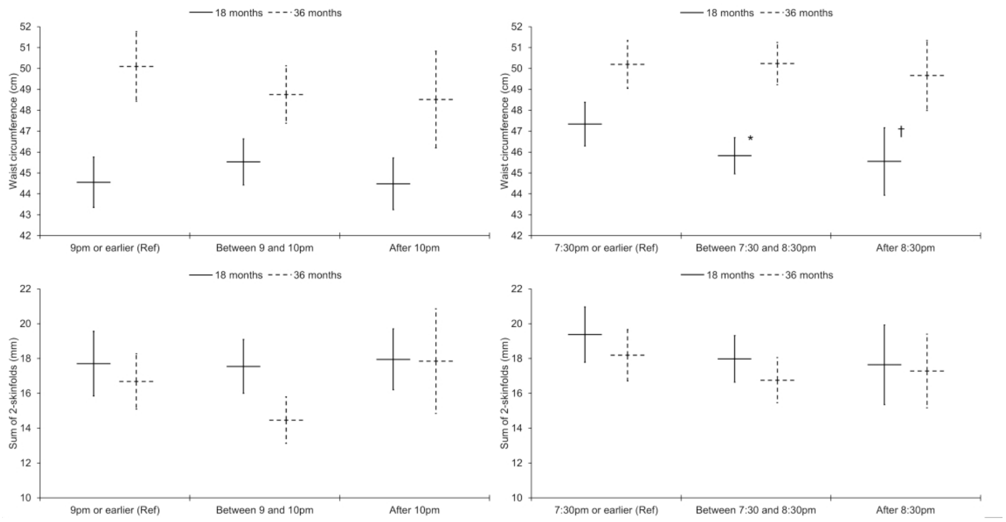

Figure 1. Associations of sleep onset categories with waist circumference and sum of 2-skinfolds, stratified by ethnicity and age group

332x171mm (300 x 300 DPI)

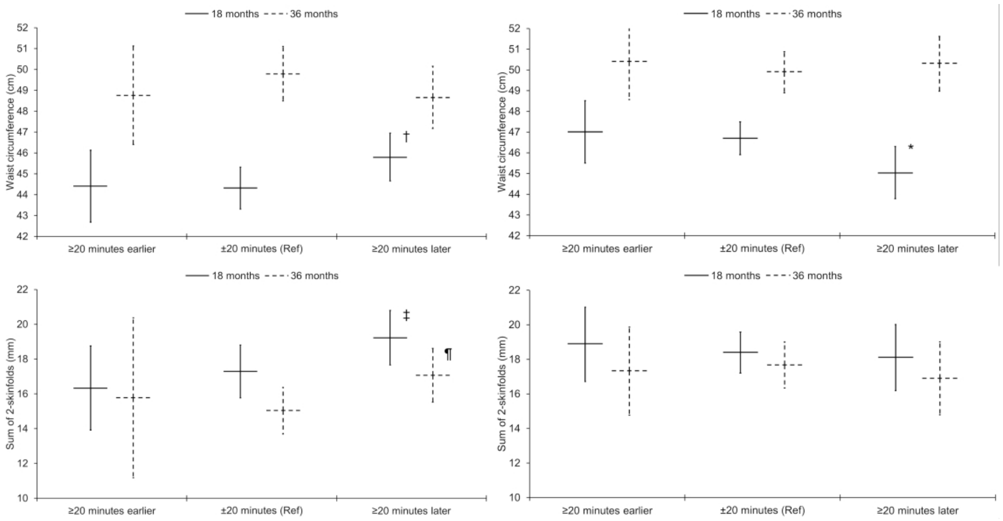

Figure 2. Associations of weekday to weekend differences in sleep onset with waist circumference and sum of 2-skinfolds, stratified by ethnicity and age group

332x171mm (300 x 300 DPI)

STROBE Statement—Checklist of items that should be included in reports of ***cohort studies***

	Item No	Recommendation	Page No
**Title and abstract**	1	(*a*) Indicate the study's design with a commonly used term in the title or the abstract	3
		(*b*) Provide in the abstract an informative and balanced summary of what was done and what was found	3
**Introduction**			
Background/rationale	2	Explain the scientific background and rationale for the investigation being reported	5
Objectives	3	State specific objectives, including any prespecified hypotheses	6
**Methods**			
Study design	4	Present key elements of study design early in the paper	6
Setting	5	Describe the setting, locations, and relevant dates, including periods of recruitment, exposure, follow-up, and data collection	6
Participants	6	(*a*) Give the eligibility criteria, and the sources and methods of selection of participants. Describe methods of follow-up	6
		(*b*) For matched studies, give matching criteria and number of exposed and unexposed	N/A
Variables	7	Clearly define all outcomes, exposures, predictors, potential confounders, and effect modifiers. Give diagnostic criteria, if applicable	6-8
Data sources/ measurement	8*	For each variable of interest, give sources of data and details of methods of assessment (measurement). Describe comparability of assessment methods if there is more than one group	6-8
Bias	9	Describe any efforts to address potential sources of bias	6-8
Study size	10	Explain how the study size was arrived at	6
Quantitative variables	11	Explain how quantitative variables were handled in the analyses. If applicable, describe which groupings were chosen and why	8
Statistical methods	12	(*a*) Describe all statistical methods, including those used to control for confounding	8
		(*b*) Describe any methods used to examine subgroups and interactions	8
		(*c*) Explain how missing data were addressed	6
		(*d*) If applicable, explain how loss to follow-up was addressed	N/A
		(*e*) Describe any sensitivity analyses	8
**Results**			
Participants	13*	(a) Report numbers of individuals at each stage of study—eg numbers potentially eligible, examined for eligibility, confirmed eligible, included in the study, completing follow-up, and analysed	6
		(b) Give reasons for non-participation at each stage	6
		(c) Consider use of a flow diagram	N/A
Descriptive data	14*	(a) Give characteristics of study participants (eg demographic, clinical, social) and information on exposures and potential confounders	9 Tables
		(b) Indicate number of participants with missing data for each variable of interest	Table 1
		(c) Summarise follow-up time (eg, average and total amount)	N/A
Outcome data	15*	Report numbers of outcome events or summary measures over time	N/A

For peer review only

Main results	16	(*a*) Give unadjusted estimates and, if applicable, confounder-adjusted estimates and their precision (eg, 95% confidence interval). Make clear which confounders were adjusted for and why they were included	9-10 Tables Figures
		(*b*) Report category boundaries when continuous variables were categorized	N/A
		(*c*) If relevant, consider translating estimates of relative risk into absolute risk for a meaningful time period	N/A
Other analyses	17	Report other analyses done—eg analyses of subgroups and interactions, and sensitivity analyses	11
**Discussion**			
Key results	18	Summarise key results with reference to study objectives	10
Limitations	19	Discuss limitations of the study, taking into account sources of potential bias or imprecision. Discuss both direction and magnitude of any potential bias	12-14
Interpretation	20	Give a cautious overall interpretation of results considering objectives, limitations, multiplicity of analyses, results from similar studies, and other relevant evidence	13-14
Generalisability	21	Discuss the generalisability (external validity) of the study results	14
**Other information**			
Funding	22	Give the source of funding and the role of the funders for the present study and, if applicable, for the original study on which the present article is based	2

*Give information separately for exposed and unexposed groups.

Note: An Explanation and Elaboration article discusses each checklist item and gives methodological background and published examples of transparent reporting. The STROBE checklist is best used in conjunction with this article (freely available on the Web sites of PLoS Medicine at http://www.plosmedicine.org/, Annals of Internal Medicine at http://www.annals.org/, and Epidemiology at http://www.epidem.com/). Information on the STROBE Initiative is available at http://www.strobe-statement.org.

For peer review only

BMJ Open

Associations of diarised sleep onset time, period, and duration with total and central adiposity in a biethnic sample of young children: the Born in Bradford observational cohort study

Journal:	*BMJ Open*
Manuscript ID	bmjopen-2020-044769.R2
Article Type:	Original research
Date Submitted by the Author:	25-Mar-2021
Complete List of Authors:	Collings, Paul; University of York, Department of Health Sciences; Bradford Institute for Health Research,<br>Blackwell, Jane; University of York, Department of Health Sciences; Leeds and York Partnership NHS Foundation Trust<br>Pal, Elizabeth; Bradford Trident, Better Start Bradford<br>Ball, Helen; Durham University, Infancy & Sleep Centre & Departmenet of Anthropology<br>Wright, John; Bradford Institute for Health Research,
**Primary Subject Heading**:	Paediatrics
Secondary Subject Heading:	Epidemiology, Public health
Keywords:	SLEEP MEDICINE, EPIDEMIOLOGY, Community child health < PAEDIATRICS, PREVENTIVE MEDICINE, PUBLIC HEALTH

SCHOLARONE™
Manuscripts

I, the Submitting Author has the right to grant and does grant on behalf of all authors of the Work (as defined in the below author licence), an exclusive licence and/or a non-exclusive licence for contributions from authors who are: i) UK Crown employees; ii) where BMJ has agreed a CC-BY licence shall apply, and/or iii) in accordance with the terms applicable for US Federal Government officers or employees acting as part of their official duties; on a worldwide, perpetual, irrevocable, royalty-free basis to BMJ Publishing Group Ltd ("BMJ") its licensees and where the relevant Journal is co-owned by BMJ to the co-owners of the Journal, to publish the Work in this journal and any other BMJ products and to exploit all rights, as set out in our licence.

The Submitting Author accepts and understands that any supply made under these terms is made by BMJ to the Submitting Author unless you are acting as an employee on behalf of your employer or a postgraduate student of an affiliated institution which is paying any applicable article publishing charge ("APC") for Open Access articles. Where the Submitting Author wishes to make the Work available on an Open Access basis (and intends to pay the relevant APC), the terms of reuse of such Open Access shall be governed by a Creative Commons licence – details of these licences and which Creative Commons licence will apply to this Work are set out in our licence referred to above.

Other than as permitted in any relevant BMJ Author's Self Archiving Policies, I confirm this Work has not been accepted for publication elsewhere, is not being considered for publication elsewhere and does not duplicate material already published. I confirm all authors consent to publication of this Work and authorise the granting of this licence.

For peer review only

Title: Associations of diarised sleep onset time, period, and duration with total and central adiposity in a biethnic sample of young children: the Born in Bradford observational cohort study

Authors: Paul J Collings[1,2], Jane E Blackwell[2,3], Elizabeth Pal[4], Helen L Ball[5], John Wright[1]

Affiliations: [1]Bradford Institute for Health Research, Bradford Teaching Hospitals NHS Foundation Trust, Bradford, England; [2]Department of Health Sciences, University of York, England; [3]Leeds and York Partnership NHS Foundation Trust, England; [4]Better Start Bradford, Bradford Trident, Bradford, England; [5]Infancy & Sleep Centre & Department of Anthropology, Department of Anthropology, Durham University, England

Corresponding author email: paul.collings@bthft.nhs.uk

ORCID: Paul J Collings: https://orcid.org/0000-0003-2022-5453; Jane E Blackwell: https://orcid.org/0000-0002-5878-8959; Elizabeth Pal: https://orcid.org/0000-0002-8682-3440; Helen L Ball: https://orcid.org/0000-0003-2255-121X; John Wright: https://orcid.org/0000-0001-9572-7293

Funding: The Born in Bradford study receives core infrastructure funding from the Wellcome Trust (grant number: WT101597MA), a joint grant from the UK Medical Research Council (MRC) and UK Economic and Social Science Research Council (ESRC) (MR/N024397/1), the British Heart Foundation (BHF) (CS/16/4/32482), and the National Institute for Health Research (NIHR) under its Collaboration for Applied Health Research and Care (CLAHRC) for Yorkshire and Humber. This study received delivery support from the NIHR Clinical Research Network. John Wright leads the Healthy Children, Healthy Families Theme of the NIHR CLAHRC in Yorkshire and Humber. Paul J Collings is funded by a BHF Immediate Postdoctoral Basic Science Research Fellowship (FS/17/37/32937). Jane E Blackwell coordinates and Paul J Collings is a member of The White Rose Child & Adolescent Sleep Research Network which is funded by a White Rose Collaboration Grant. The views expressed in this paper are those of the authors and not necessarily those of the MRC, ESRC, BHF, NIHR, and UK Department of Health or National Health Services or of any other funder acknowledged here.

Competing interests: None declared

Contributions: John Wright is Chief Investigator of the Born in Bradford study and setup the cohort; Helen L Ball created the parent-reported sleep diary; Paul J Collings initiated the study, planned and performed the statistical analyses, and wrote the manuscript; all authors including Jane Blackwell and Elizabeth Pal critically revised the manuscript for intellectual content, helped interpret study findings, read and approved the final version and are accountable for the work.

For peer review only

Data availability: Data generated and analysed for the current study are available from the corresponding author on reasonable request. **Word count**: 4714

For peer review only

Abstract

Objectives: To investigate associations of parent-reported sleep characteristics with adiposity levels in a biethnic sample of young children

Design: Cross-sectional observational study

Setting: The Born in Bradford 1000 study, UK

Participants: Children aged approximately 18 months (m) (*n*=209; 40.2% South Asian; 59.8% White) and 36m (*n*=162; 40.7% South Asian; 59.3% White)

Primary and secondary outcome measures: Children's body mass index (BMI) z-score, sum of 2-skinfolds (triceps and subscapular), and waist circumference. Adjusted regression was used to quantify associations of sleep parameters with adiposity stratified by ethnicity and age group. The results are beta-coefficients (95% confidence intervals) and represent the difference in outcomes for every one hour difference in sleep parameters.

Results: The average sleep onset time was markedly later in South Asian (9:26pm ± 68 minutes) than White children (7:41pm ± 48 minutes). Later sleep onset was associated with lower BMI z-score (-0.3 (-0.5 to -0.0)) and sum of 2-skinfolds (-1.5 (-2.8 to -0.2) mm) in White children aged 18m and higher BMI z-score in South Asian children aged 36m (0.3 (0.0 to 0.5)). Longer sleep duration on weekends than weekdays was associated with higher BMI z-score (0.4 (0.1 to 0.8)) and waist circumference (1.2 (0.3 to 2.2) cm) in South Asian children aged 18m, and in the same group of children later sleep onset on weekends than weekdays was associated with larger sum of 2-skinfolds (1.7 (0.3 to 3.1) mm) and waist circumference (1.8 (0.6 to 2.9) cm). Going to sleep ≥20 minutes later on weekends than weekdays was associated with lower waist circumference in White children aged 18m (-1.7 (-3.2 to -0.1) cm).

Conclusions: Sleep timing is associated with total and central adiposity in young children but associations differ by age group and ethnicity. Sleep onset times and regular sleep schedules may be important for obesity prevention.

For peer review only

Strengths and limitations of this study

- This is the first study to investigate myriad sleep dimensions and how they relate to direct markers of adiposity in young children
- It is also the first study to investigate differences in these associations between UK born South Asian and White children living in a deprived urban setting
- Sleep parameters were mutually adjusted for each other, and the analyses were adjusted for a broad range of covariates including potential mediating factors
- Residual confounding and mediating effects may have persisted because of measurement imprecision in parent-reported covariates, and directions of association between exposures and outcomes cannot be inferred from this cross-sectional study
- The proportion of parents who completed sleep diaries was low, meaning the study sample is susceptible to selection biases, and when stratified by ethnicity and age group some groups were small

For peer review only

Introduction

Many studies have investigated associations of sleep duration or the sleep period (elapsed time between sleep onset and termination regardless of waking intervals) with adiposity in school-aged children and youth [1,2]. Comparatively few studies have been conducted in infants and toddlers [3,4] despite sleep problems and sleep loss being prevalent in children aged <36 months (m) [5]. Beyond sleep duration, it is beginning to be recognised that sleep is a multidimensional construct of partly overlapping dimensions, and that parameters such as sleep timing may also be influential with regard to adiposity level and obesity risk [6].

Aspects of sleep timing include bedtimes, time of sleep onset, and variability in sleeping patterns such as differences between weekday and weekend sleep schedules. Preliminary evidence indicates that later bedtimes are associated with higher weight status in pre-schoolers [7–10] and primary school-aged children [11], and that later sleep timing on weekends than weekdays is correlated with higher risk of overweight and obesity [12]. Hypothesised mechanisms include circadian dysrhythmia causing hormone-mediated preferences for unhealthy calorific foodstuffs [13,14], the effect of which may be compounded by later sleep onset enabling more time to eat and more opportunity for screen time [15], and lower daily physical activity and higher sedentariness due to delayed waking and fatigue [16]. More studies are needed to investigate the independent associations of myriad sleep parameters with childhood adiposity and to ascertain the underlying mechanisms of action. A research focus on young children is important, to determine when associations between sleep parameters with adiposity first emerge, and to provide information about how to intervene by identifying modifiable target behaviours [6].

Early childhood is a critical window for obesity prevention because it is a period when rapid weight gain occurs and behavioural patterns are first established [17,18]. Although an increasing number of studies have investigated sleep duration [3,4], few have investigated associations of sleep timing or variability with adiposity in early childhood [19–21], and additional investigation of ethnic minority and socioeconomically impoverished populations is needed. Children of non-White ethnicity and children from deprived backgrounds have unfavourable sleeping patterns and are high-risk for early onset obesity [22,23]. We have previously shown that UK South Asian children who live in a deprived urban setting go to bed much later than White children from the same area [24]. It is important to determine if shifted sleep schedules may in part explain why obesity rates are higher in South Asian than White children in the UK [25], and why South Asian school-aged children have more centrally-stored adiposity which is metabolically harmful [26]. This information could help to reduce ethnic disparities in disease risk by facilitating behaviour change interventions and policy guidelines that are tailored to the needs of specific ethnic groups.

For peer review only

This study examined independent associations of a broad range of sleep parameters including diarised sleep onset time, period and duration, and weekday to weekend variations in sleep parameters, with total and central adiposity in a young biethnic sample of UK children from a deprived urban setting.

Methods

This study was conducted in Bradford which is the fifth largest local authority in England and one of the most deprived and ethnically diverse cities in the UK [27]. Bradford has an overweight and obesity prevalence of 21.8% in 4 to 5 year old children and 38.4% in 10 to 11 year olds [28]. The Born in Bradford (BiB) 1000 study [29], nested within the larger BiB pregnancy cohort [30], aims to investigate modifiable risk factors for childhood obesity. Pregnant women (*n*=1916) were invited to the BiB 1000 study when they attended routine hospital appointments, 90.6% accepted the invitation (*n*=1735). Consent to medical records access was provided and periodic postnatal assessments were carried out when the women's offspring were approximately 6, 12, 18, 24, and 36m old. Parents who participated when children were aged about 18m (*n*=1228; 70.7% of all BiB 1000 participants) or 36m (*n*=1232; 71%) were asked to complete a sleep diary for their child. At each timepoint diaries were completed for approximately 15% of all BiB 1000 participants (18m: *n*=276; 36m: *n*=262). The diary data were collected between October 2010 and September 2012. This complete-case analysis included only children with sleep diary data, concurrent adiposity measurements and information about potentially important covariates. Maternal ethnicity was used as a proxy for child ethnicity. Children with a mother belonging to an ethnic group other than South Asian (Pakistani / Indian / Bangladeshi) or White (British / other) were excluded from the study due to small numbers. All children were born in the UK (most children of South Asian heritage had Pakistani origin mothers (85%) and most White children had White British mothers (94%)). For brevity we refer to children as being of South Asian or White ethnicity. The final sample included 209 children aged 18m (12% of all BiB 1000 participants) and 162 children aged 36m (9.3%). Ethical approval for all aspects of the study was granted by Bradford Research Ethics Committee (Reference 07/H1302/112) and all mothers provided informed written consent for participation.

Sleep parameters

Parents completed sleep diaries, providing free-text responses about the time their child fell asleep on an evening and woke-up the next morning. The sleep period was calculated as elapsed time between sleep onset and waking the next day. Because the sleep period does not account for periods of overnight waking which can be common in young children [31] parents also reported their child's overnight sleep duration. Parents completed diaries on two weekdays and a weekend day. Mean weekday values were calculated by averaging data for

For peer review only

the two weekdays; thereafter daily averages over the course of a week were calculated using weekday to weekend weighting (in the ratio of 5:2). Differences in sleep parameters between weekdays and weekends were calculated by subtraction (weekend – weekday values).

Adiposity markers

Child weight and height were assessed by trained researchers and BMI (kg/m^2) and *z*-scores were calculated [32]. Waist circumference was measured at the level of the navel. Skinfolds of the left triceps and subscapular were measured to the nearest 0.1mm and summed. Reliability metrics indicated good intra- and inter-observer technical error for measurements [33].

Covariates

Potential confounders and mediators of associations were selected based on previous evidence linking them with sleep and obesity [17,22]. A multidimensional marker of socioeconomic status was synthesised from information collated during interviews with parents (usually mothers) about their education, employment, housing tenure, financial situation, and ownership of goods. For the purposes of this study, from an initial five categories [34], children were classified as belonging to one of three parental socioeconomic status groups: least deprived (least socioeconomically deprived and most educated), moderately deprived (employed and not materially deprived / employed but no access to money) and most deprived (benefits but not materially deprived / most economically deprived). Maternal age was self-reported in pregnancy whereas the number of previous births (used to group mothers as primaparous (pregnant for the first time) or not), child gender, gestational age, and birth weight were extracted from medical records. Maternal height and weight were measured at the 18 and 36m timepoints and were used to calculate maternal BMI. Parents reported whether or not their child napped on sleep diary days, these data were used to create three categories of napping frequency (never: napped zero days; occasionally: napped 1 or 2 days; every day: napped all three days). On each sleep diary reporting day, parents also provided free-text responses about the time their child ate their last meal of the day. Mean weekday values were calculated by averaging data across the two weekdays, and weekday to weekend weighting was used to calculate the average daily time that the child ate their last meal; differences between weekdays and weekends were calculated by subtraction. Infant dietary data were collected using a validated food frequency questionnaire that was modified to include ethnic-specific foodstuffs. Dietary constructs indicative of unhealthy snacking (frequency of biscuit, crisps, cakes, sweets, chocolate, sugar-sweetened beverage consumption) and fruit and vegetable consumption in the previous 4-12 weeks were derived [24]. Parents reported the number of hours their child

For peer review only

watched TV on a typical weekday and weekend day; weighting was used to calculate average daily TV viewing and weekday to weekend differences were calculated [35]. When children were aged 36m a parent-reported Early Years Physical Activity Questionnaire (EY-PAQ) was used to estimate children's physical activity level [36]. Calendar dates of the 18 and 36m assessments were recorded and categorised by season (summer / autumn or spring / winter).

Patient and public involvement (PPI)

The BiB research team regularly convenes a Parent Governors PPI group, BiB participants who are now aged 10 to 12 years can become Young Ambassadors of the study, and BiB runs regular community events and science festivals (https://borninbradford.nhs.uk/news-events/events/). These committees and events help to shape our research by allowing us to learn about the opinions, concerns and ideas of the community. A study author (JEB) recently chaired a PPI event held in conjunction with The Sleep Charity [37]. Attendees were parents of children with sleep difficulties, staff who work in residential settings with children, sleep practitioners and representatives from parent and carer forums. Emerging themes from the session included: 1) there is a lack of information about assisting development of healthy sleep routines in infants, 2) more information and knowledge about the consequences of poor sleep in infants is needed, and 3) early intervention is vital and parents of young children should be offered evidence-based sleep interventions for their child.

Statistics

Correlations between individual sleep parameters and adiposity markers were calculated using Pearson or Spearman methods as appropriate [38]. Adjusted linear regression was used to investigate cross-sectional associations between sleep parameters with adiposity. Model 1 adjusted associations for sex, age, socioeconomic status, maternal pregnancy age, parity, gestational age, birthweight, maternal BMI, season of measurement, and napping frequency. Model 2 further adjusted for potential confounding or mediating factors, including unhealthy snacking, fruit and vegetable intake, and TV viewing. Model 3 mutually adjusted sleep period and duration variables for sleep onset time, and vice versa. All analyses were performed stratified by ethnic group and age group. Interaction effects between sleep period and duration with sleep onset time were examined by introducing multiplicative terms to models (e.g. sleep onset*duration). Nonlinearity of associations was also examined by adding quadratic terms for sleep parameters. There was some of evidence for nonlinear associations between average daily sleep onset and weekday to weekend differences in sleep onset with waist circumference and sum of 2-skinfolds, hence sleep onset variables were additionally trichotomised and adjusted linear regression was used to estimate group

For peer review only

marginal means (and 95% confidence intervals (CI)) for these outcomes. Data for the sum of 2-skinfolds were slightly skewed, but because results were consistent regardless of whether the data were log-transformed or not, results based on the untransformed data are presented. Sensitivity analyses further adjusted associations for the time of last meal and physical activity (not applicable to 18m models when physical activity was not assessed), potentially important covariates but missing data; models with waist circumference or sum of 2-skinfolds as dependent variables were further adjusted for height. Analyses were performed with Stata/SE 16.1 software (StataCorp, College Station, TX). Variance inflation factors were calculated to highlight potential multicollinearity and $p<0.05$ was deemed to indicate statistically significant associations, but all results are interpreted with emphasis on the range of plausible values of associations as shown by confidence intervals [39].

Results

Sample characteristics

Participant details are shown in **Table 1**. Child ages ranged from 16.4 to 22.8m and 35.5 to 39.5m at each respective timepoint. The average daily sleep onset time was consistently after 9pm in South Asian children and before 8pm in White children. Sleep onset time was inversely correlated with the sleep period (South Asian children aged 18m: -0.52; South Asian children aged 36m: -0.38; White children aged 18m: -0.52; White children aged 36m: -0.37) and to a lesser extent sleep duration (South Asian children aged 18m: -0.42; South Asian children aged 36m: -0.10; White children aged 18m: -0.38; White children aged 36m: -0.27). Correlations between the sleep period and sleep duration were weaker in South Asian than White children (South Asian children aged 18m: 0.72; South Asian children aged 36m: 0.66; White children aged 18m: 0.81; White children aged 36m: 0.83). Correlations between adiposity markers differed by ethnicity and age group, but consistently BMI z-score was more strongly correlated with waist circumference (South Asian children aged 18m: 0.69; South Asian children aged 36m: 0.71; White children aged 18m: 0.61; White children aged 36m: 0.79) than the sum of 2-skinfolds (South Asian children aged 18m: 0.54; South Asian children aged 36m: 0.28; White children aged 18m: 0.44; White children aged 36m: 0.53); correlations between waist circumference and the sum of 2-skinfolds were weakest (South Asian children aged 18m: 0.47; South Asian children aged 36m: 0.19; White children aged 18m: 0.26; White children aged 36m: 0.47).

South Asian children

Table 2 shows that in South Asian children aged 18m there were no significant associations between average daily sleep parameters with adiposity. In children aged 36m, there was some evidence in model 3 that later daily sleep onset was associated with higher BMI *z*-score; the association attenuated when further adjusted for final mealtime (β=0.2 (95% CI: -

For peer review only

0.2 to 0.6), p=0.34). There were no other significant associations for average daily sleep parameters. **Figure 1** presents the group marginal means for waist circumference and the sum of 2-skinfolds stratified by sleep onset categories; there were no group differences. With regard to weekday to weekend differences, Table 2 shows that longer sleep duration on weekends than weekdays was consistently associated with higher adiposity in South Asian children aged 18m, and in the same group of children, later sleep onset on weekends than weekdays was associated with larger waist circumference and sum of 2-skinfolds. In South Asian children aged 36m, later sleep onset on weekends than weekdays was associated with larger sum of 2-skinfolds in model 2, but the association attenuated when further adjusted for the sleep period. **Figure 2** shows the adjusted marginal means for waist circumference and sum of 2-skinfolds stratified by three categories of weekday to weekend differences in sleep onset. There was some evidence that compared to children with consistent sleep onset times (±20 minutes of each other), children aged 18m who went to sleep ≥20 minutes later on weekends than weekdays had larger waist circumferences (1.5 (-0.1 to 3.0) cm, p=0.060) and sum of 2-skinfolds (1.9 (-0.2 to 4.1) mm, p=0.078). Similarly, there was some indication that aged 36m, children who went to sleep ≥20 minutes later on weekends than weekdays had larger sum of 2-skinfolds (2.0 (-0.2 to 4.3) mm, p=0.074).

White children

Table 3 shows that for White children aged 36m there were no significant associations, although there was some indication that later sleep onset on weekends than weekdays was associated with smaller sum of 2-skinfolds. In children aged 18m, independent of all covariables including sleep period and duration, later daily sleep onset was associated with smaller sum of 2-skinfolds and lower BMI z-score (though further adjustment for final mealtime attenuated the latter association (-0.2 (-0.5 to 0.2), p=0.28). Figure 1 illustrates that at age 18m, compared to children with a sleep onset time of 7:30pm or earlier, children who began to sleep between 7:30 and 8:30pm had smaller waist circumferences (-1.5 (-2.9 to -0.1) cm, p=0.035). Figure 2 highlights that children aged 18m who went to sleep ≥20 minutes later on weekends than weekdays had smaller waist circumferences than children with consistent sleep onset times (-1.7 (-3.2 to -0.1) cm, p=0.038).

Sensitivity analyses

Further adjustment for physical activity in models that included children aged 36m, and adjustment for height when waist circumference and the sum of 2-skinfolds were modelled as dependent variables, did not influence any of the reported associations. There was no evidence for interaction effects between sleep onset with the sleep period or sleep duration. Variance inflation factors were within acceptable limits (<10; the majority were <2.5).

For peer review only

Discussion

This study investigated associations of diarised sleep onset time, period and duration, and weekday to weekend differences in sleep parameters, with total and central adiposity in young children aged 18 and 36m. We discovered age- and ethnicity-specific associations for sleep onset times, which we in part attribute to markedly different sleep schedules between ethnic groups; South Asian children went to sleep on average nearly two hours later than White children. Later average sleep onset and later sleep timing on weekends than weekdays were associated with higher adiposity in South Asian children, and conversely with lower adiposity in White children, particularly those aged 18m. Longer sleep durations on weekends than weekdays were associated with higher adiposity in South Asian children aged 18m.

Numerous systematic reviews have summarised the evidence for associations of sleep duration or the sleep period with adiposity in children and adolescents [1,2]. Of note, one review meta-analysed the results of six prospective studies totalling 14,264 children aged <36m. There was moderate heterogeneity in results, but each additional hour of sleep was associated with a negative change in BMI z-score over follow-up [3]. Limitations of the existing evidence include a preponderance of studies that have relied solely on BMI or BMI z-score as proxies for total adiposity. Studies have also considered few potential confounding or mediating factors and focussed solely on sleep duration. Sleep is a multidimensional construct and it is important to unravel the independent relations of specific dimensions with adiposity. In a previous BiB 1000 study we analysed repeated questionnaire data collected at four timepoints (12, 18, 24 and 36m) to quantify associations between parent reported sleep duration with measured adiposity in 1338 UK South Asian and White children. Longer sleep duration predicted lower total and central adiposity but only in South Asian children [24]. Here, in a sub-sample of the same cohort, we were able to scrutinise and mutually adjust for myriad sleep parameters collected over the course of three days via parent-completed sleep diaries. Independent of sleep length, the average sleep onset time and variability in sleep behaviours between weekdays and weekends emerged as the key modifiable dimensions that predicted adiposity in South Asian and White children.

A recent review concluded that sleep timing, in particular later bedtimes, are associated with higher weight status in primary school-aged children [11]. Similarly, the few studies conducted thus far in 4 to 5 year olds indicate that later bedtimes, and most markedly bedtimes after 9pm, are associated with higher BMI z-score and obesity risk [7–9]. A particular study found that an association of short sleep with higher BMI z-score was only evident in children who went to bed after 9pm [10]. Just two studies have been performed in younger children. Zhang et al. found no differences in internally derived BMI z-scores

For peer review only - http://bmjopen.bmj.com/site/about/guidelines.xhtml

between early and late bedtime groups (separated using a crude median split at about 8pm) in a sample of children with ages ranging from 12 to 26m [19]. Roy et al. found that later bedtimes in children aged 36m were associated with higher odds of obesity, but the association attenuated when adjusted for sleep duration [20]. To our knowledge, this is the first study to show that independent of the sleep period, later sleep onset is associated with higher BMI z-score in South Asian children aged 36m. The association attenuated when adjusted for final mealtime which provides some indication that later sleep onset may elevate obesity risk via later evening meals and higher caloric intake later in the day [40]. In line with this hypothesis, animal models and clinical trials have shown that early and time-restricted feeding has numerous physiological benefits, including lower adiposity [41,42].

With regard to sleep variability, a pooled analysis of five studies conducted in school-aged children and youth indicated that later sleep timing on weekends than weekdays was correlated with higher risk of overweight or obesity, albeit with a small effect size [12]. In toddlers, a recent study found that inconsistent sleep onset times over the course of a week predicted higher BMI *z*-scores, independent of device-measured physical activity and diet quality, and that inconsistent sleep indirectly explained an association between household poverty with higher BMI z-score [21]. We uniquely observed that later sleep timing on weekends than weekdays was associated with higher adiposity in South Asian children aged 18m and larger sum of 2-skinfolds in South Asian children aged 36m, although the association in older children only approached statistical significance. The associations in children aged 18m were independent of potential confounding or mediating factors including TV viewing, unhealthy snacking, fruit and vegetable intake, and the final mealtime. It may be that these are not important mediators in very young children who are less autonomous and for whom a large part of their diet and screen time is governed by parents and carers. That said, we cannot exclude residual confounding or mediating effects as all covariate data were parent-reported and are subject to random error and bias. Longer sleep duration on weekends than weekdays was associated with higher adiposity in South Asian children aged 18m. This suggests that consistent sleep durations are important.

We observed that later daily sleep onset was associated with lower BMI z-score in White children aged 18m. This observation may appear counterintuitive, but Roy et al. similarly reported that independent of overnight sleep duration, each one hour later to bed was associated with -0.08 lower BMI z-score in 878 children aged 24 months, although their confidence interval narrowly crossed the null of no association (95% CI: -0.17 to 0.01) [20]. Following adjustment for final mealtime our result for BMI z-score also attenuated to the null, but inverse associations with more direct adiposity indicators remained, including smaller sum of 2-skinfolds and waist circumference as a function of later sleep onset. These

For peer review only

unexpected results may partly be explained by early sleep onset obstructing time during early-to-mid evenings that could otherwise be spent physically active. Time-stamped accelerometer studies show that daily physical activity patterns are bimodal in 18m olds. Physical activity levels first peak in the morning, slump after midday due to feeding and napping (81% of our White children aged 18m napped every day), after which there is a second larger peak that begins around 4pm and tails-off by 8pm [43,44]. In our sample of White children aged 18m, the earliest sleep onset times were around 6pm and more than one-third (36.8%) were asleep by 7:30pm. It is conceivable that these children may have missed a substantial part of the evening peak in physical activity, thus contributing to higher adiposity [33,45]. Our related observation in the same group of children, that going to sleep ≥20 minutes later on weekends than weekdays was associated with lower waist circumference, may be explained by later sleep onset at the weekend enabling more opportunity to be physically active [46].

Although White children aged 36m exhibited a similar distribution of sleep onset times compared to their younger White peers, there were few comparable associations, apart from some indication in children aged 36m that later sleep onset on weekends than weekdays was associated with lower adiposity (an association with the sum of 2-skinfolds approached statistical significance). The apparent age-related differences may be explained by daily physical activity patterns following a different trajectory in slightly older children who tend not to nap (just 5% of White children aged 36m napped every day; 46% napped occasionally; 49% did not nap). Instead of a naptime-related slump after midday which is followed by an evening peak, physical activity levels tend to be relatively constant throughout the afternoon, before progressively declining in the evening and most notably from 7pm onwards [47]. Hence, in many White children aged 36m, there may have been no peak in evening physical activity for early sleep to obstruct. In contrast to our results, Roy et al. did find a significant association between later bedtimes with lower BMI z-score in children aged approximately 36m [20]. That study was performed in children living in America, Australia and New Zealand which may account for the discrepancy. In those countries frequent daytime naps persist in older children partly because of designated naptimes in childcare [48–50]. Hence many children aged 3 to 4 years continue to exhibit a bimodal physical activity pattern, including an early-to-mid evening activity peak, which early sleep might obstruct [51].

Sleep recommendations for children have historically focussed on duration rather than other sleep dimensions [52]. Our results highlight that contemporary sleep guidelines should further acknowledge the importance of sleep timing and regular sleep schedules. Any such recommendations should also be considerate of ethnic and cultural differences in sleeping patterns, including what may and may not be feasible and acceptable to different population

For peer review only

subgroups. Our results for White children aged 18m, some of whom were already asleep just after 6pm, indicate that going to sleep too early could be detrimental from an energy-balance and obesity risk perspective. Sleep onset times were much later in South Asian children who may benefit from an earlier evening meal, earlier sleep onset, and consistent sleep schedules across week and weekend days. It is difficult to decipher if any such changes would translate to clinically meaningful differences in adiposity levels because young children tend to be healthy. However, more than half (54%) of South Asian children aged 18m went to sleep later on weekends than weekdays, on average by 32 (± 23) minutes. According to our point estimates this could be associated with up to 1cm larger waist circumference. This is unlikely trivial considering that any beneficial influence on adiposity levels this early in childhood may be important. It is a critical period when rapid weight gain occurs and sustained obesity can develop [18].

This study uniquely investigated associations of diarised sleep parameters with myriad adiposity markers in a biethnic sample of young children from a deprived urban setting, which is a high-risk group for sleep problems and obesity [22,23]. Sleep diaries enabled information about numerous dimensions of sleep to be collated but unfortunately they incur participant burden. This likely contributed to only a minority of BiB 1000 parents choosing to complete sleep diaries for their child, the consequences of which are at least twofold. First, our study sample is subject to selection biases. Compared to the full BiB pregnancy cohort, which was broadly representative of the obstetric population in Bradford at the time of recruitment, the proportion of families who were most deprived was lower in this study (28% versus 45%) [34]. This hinders generalisability, but it is reassuring that there was still variability within the sample in parental socioeconomic status. Second, stratifying by ethnicity and age group meant that some of our analyses were based on relatively few observations and low statistical power. This latter issue was compounded by missing data for specific items such as sleep duration (which was only reported if parents considered they knew how long their child had slept overnight) and the sum of 2-skinfolds. The results should therefore be considered exploratory and require replication in larger samples. It is advantageous that we investigated more direct adiposity markers than weight-for-height proxies which tend to underestimate fatness in South Asians [53], and that in addition to skinfolds we investigated waist circumferences, as South Asian children eventually develop more centrally-stored adiposity which is metabolically deleterious [26]. Another strength is that each of our analyses were adjusted for a broad range of covariates, including mutual adjustment for sleep parameters, to tease apart the independent associations of distinct sleep dimensions with adiposity. It is a weakness that we did not have information about napping durations which would have permitted investigation of napping and 24-hour sleeping patterns with

For peer review only

outcomes. Furthermore, directions of association cannot be inferred from this cross-sectional study since it is possible that adiposity could influence sleeping habits and vice versa [24]. Longitudinal studies or trials are needed to determine directions of association. Future studies may achieve a better trade-off between participant retention and data resolution if they use sensor-based technologies rather than diaries [54] although a combination of methods may prove best to capture habitual sleep parameters [55].

Conclusions

Sleep onset times appear to be independently associated with levels of total and central adiposity in young South Asian and White children. Suitable sleep times and consistent sleep schedules across week and weekend days may be important modifiable determinants of early childhood obesity.

For peer review only

References

1 Matricciani L, Paquet C, Galland B, *et al.* Children's sleep and health: A meta-review. *Sleep Med Rev* 2019;**46**:136–50. doi:10.1016/j.smrv.2019.04.011

2 Sun J, Wang M, Yang L, *et al.* Sleep duration and cardiovascular risk factors in children and adolescents: A systematic review. *Sleep Med Rev* 2020;**53**:101338. doi:10.1016/j.smrv.2020.101338

3 Miller MA, Kruisbrink M, Wallace J, *et al.* Sleep duration and incidence of obesity in infants, children, and adolescents: a systematic review and meta-analysis of prospective studies. *Sleep* 2018;**41**:1–19. doi:10.1093/sleep/zsy018

4 Harskamp-van Ginkel MW, Chinapaw MJM, Harmsen IA, *et al.* Sleep during Infancy and Associations with Childhood Body Composition: A Systematic Review and Narrative Synthesis. *Child Obes* 2020;**16**:94–116. doi:10.1089/chi.2019.0123

5 Ophoff D, Slaats MA, Boudewyns A, *et al.* Sleep disorders during childhood: a practical review. *Eur J Pediatr* 2018;**177**:641–8. doi:10.1007/s00431-018-3116-z

6 Miller AL, Lumeng JC, LeBourgeois MK. Sleep patterns and obesity in childhood. *Curr Opin Endocrinol diabetes Obes* 2015;**22 SRC**-:41–7.

7 Scharf RJ, Deboer MD. Sleep timing and longitudinal weight gain in 4- and 5-year-old children. *Pediatr Obes* 2015;**10**:141–8. doi:10.1111/ijpo.229

8 Anderson SE, Andridge R, Whitaker RC. Bedtime in Preschool-Aged Children and Risk for Adolescent Obesity. *J Pediatr* 2016;**176**:17–22. doi:10.1016/j.jpeds.2016.06.005

9 Simon SL, Goetz AR, Meier M, *et al.* Sleep duration and bedtime in preschool-age children with obesity: Relation to BMI and diet following a weight management intervention. *Pediatr Obes* 2019;**14**:1–9. doi:10.1111/ijpo.12555

10 Miller AL, Kaciroti N, Lebourgeois MK, *et al.* Sleep timing moderates the concurrent sleep duration-body mass index association in low-income preschool-age children. *Acad Pediatr* 2014;**14**:207–13. doi:10.1016/j.acap.2013.12.003

11 Morrissey B, Taveras E, Allender S, *et al.* Sleep and obesity among children: A systematic review of multiple sleep dimensions. *Pediatr Obes* 2020;**15**:1–21. doi:10.1111/ijpo.12619

12 Sun W, Ling J, Zhu X, *et al.* Associations of weekday-to-weekend sleep differences with academic performance and health-related outcomes in school-age children and youths. *Sleep Med Rev* 2019;**46**:27–53. doi:10.1016/j.smrv.2019.04.003

13 Petrov ME, Vander Wyst KB, Whisner CM, *et al.* Relationship of Sleep Duration and Regularity with Dietary Intake Among Preschool-Aged Children with Obesity from Low-Income Families. *J Dev Behav Pediatr* 2017;**38**:120–8. doi:10.1097/DBP.0000000000000369

For peer review only

14 Jansen EC, Peterson KE, Lumeng JC, *et al.* Associations between Sleep and Dietary Patterns among Low-Income Children Attending Preschool. *J Acad Nutr Diet* 2019;**119**:1176–87. doi:10.1016/j.jand.2019.01.008

15 Moorman JD, Morgan P, Adams TL. The Implications of Screen Media Use for the Sleep Behavior of Children Ages 0–5: a Systematic Review of the Literature. *Curr Sleep Med Reports* 2019;**5**:164–72. doi:10.1007/s40675-019-00151-0

16 Olds TS, Maher CA, Matricciani L. Sleep Duration or Bedtime? Exploring the Relationship between Sleep Habits and Weight Status and Activity Patterns. *Sleep* 2011;**34**:1299–307. doi:10.5665/sleep.1266

17 Woo Baidal JA, Locks LM, Cheng ER, *et al.* Risk Factors for Childhood Obesity in the First 1,000 Days. *Am J Prev Med* 2016;**50**:761–79. doi:10.1016/j.amepre.2015.11.012

18 Geserick M, Vogel M, Gausche R, *et al.* Acceleration of BMI in Early Childhood and Risk of Sustained Obesity. *N Engl J Med* 2018;**379**:1303–12. doi:10.1056/NEJMoa1803527

19 Zhang Z, Pereira JR, Sousa-Sá E, *et al.* The cross-sectional and prospective associations between sleep characteristics and adiposity in toddlers: Results from the GET UP! Study. *Pediatr Obes* 2019;**14**. doi:10.1111/ijpo.12557

20 Roy M, Haszard JJ, Savage JS, *et al.* Bedtime, body mass index and obesity risk in preschool-aged children. *Pediatr Obes* 2020;:ijpo.12650. doi:10.1111/ijpo.12650

21 Covington L, Armstrong B, Trude ACB, *et al.* Longitudinal Associations Among Diet Quality, Physical Activity and Sleep Onset Consistency With Body Mass Index z -Score Among Toddlers in Low-income Families. *Ann Behav Med* Published Online First: 16 November 2020. doi:10.1093/abm/kaaa100

22 Blair PS, Humphreys JS, Gringras P, *et al.* Childhood Sleep Duration and Associated Demographic Characteristics in an English Cohort. *Sleep* 2012;**35**:353–60. doi:10.5665/sleep.1694

23 Lu Y, Pearce A, Li L. Distinct patterns of socio-economic disparities in child-to-adolescent BMI trajectories across UK ethnic groups: A prospective longitudinal study. *Pediatr Obes* 2020;**15**:1–9. doi:10.1111/ijpo.12598

24 Collings PJ, Ball HL, Santorelli G, *et al.* Sleep Duration and Adiposity in Early Childhood: Evidence for Bidirectional Associations from the Born in Bradford Study. *Sleep* 2017;**40**. doi:10.1093/sleep/zsw054

25 Hudda MT, Nightingale CM, Donin AS, *et al.* Patterns of childhood body mass index (BMI), overweight and obesity in South Asian and black participants in the English National child measurement programme: effect of applying BMI adjustments standardising for ethnic differences in BMI-body fatness asso. *Int J Obes* 2018;**42**:662–70. doi:10.1038/ijo.2017.272

For peer review only

26 Wolf RM, Nagpal M, Magge SN. Diabetes and cardiometabolic risk in south Asian youth: A review. *Pediatr Diabetes* 2020;**Online**:1–15. doi:10.1111/pedi.13078
27 City of Bradford Metropolitan District Council. Understanding Bradford District. 2020.https://ubd.bradford.gov.uk/ (accessed 22 Apr 2020).
28 Local Government Association (LGA) Research. National Child Measurement Programme data for Bradford. https://lginform.local.gov.uk/reports/view/lga-research/latest-data-from-the-national-child-measurement-programme-ncmp?mod-area=E08000032&mod-group=AllRegions_England&mod-type=namedComparisonGroup (accessed 23 Apr 2020).
29 Bryant M, Santorelli G, Fairley L, *et al.* Design and characteristics of a new birth cohort, to study the early origins and ethnic variation of childhood obesity: the BiB1000 study. *Longit Life Course Stud* 2013;**4**:119–35. doi:10.14301/llcs.v4i2.221
30 Wright J, Small N, Raynor P, *et al.* Cohort profile: The born in bradford multi-ethnic family cohort study. *Int J Epidemiol* 2013;**42**:978–91. doi:10.1093/ije/dys112
31 Sadeh A, Mindell JA, Luedtke K, *et al.* Sleep and sleep ecology in the first 3 years: A web-based study. *J Sleep Res* Published Online First: 2009. doi:10.1111/j.1365-2869.2008.00699.x
32 Cole TJ, Freeman JV, Preece MA. Body mass index reference curves for the UK, 1990. *Arch Dis Child* 1995;**73**:25–9. doi:10.1136/adc.73.1.25
33 Collings PJ, Brage S, Bingham DD, *et al.* Physical Activity, Sedentary Time, and Fatness in a Biethnic Sample of Young Children. *Med Sci Sport Exerc* 2017;**49**:930–8. doi:10.1249/MSS.0000000000001180
34 Fairley L, Cabieses B, Small N, *et al.* Using latent class analysis to develop a model of the relationship between socioeconomic position and ethnicity: cross-sectional analyses from a multi-ethnic birth cohort study. *BMC Public Health* 2014;**14**:835. doi:10.1186/1471-2458-14-835
35 Collings PJ, Kelly B, West J, *et al.* Associations of TV Viewing Duration, Meals and Snacks Eaten When Watching TV, and a TV in the Bedroom with Child Adiposity. *Obesity* 2018;**26**:1619–28. doi:10.1002/oby.22288
36 Bingham D, Collings P, Clemes S, *et al.* Reliability and Validity of the Early Years Physical Activity Questionnaire (EY-PAQ). *Sports* 2016;**4**:30. doi:10.3390/sports4020030
37 The Sleep Charity. https://thesleepcharity.org.uk/ (accessed 28 Aug 2020).
38 de Winter JCF, Gosling SD, Potter J. Comparing the Pearson and Spearman correlation coefficients across distributions and sample sizes: A tutorial using simulations and empirical data. *Psychol Methods* 2016;**21**:273–90. doi:10.1037/met0000079

For peer review only

39 Amrhein V, Greenland S, McShane B. Scientists rise up against statistical significance. *Nature* 2019;**567**:305–7. doi:10.1038/d41586-019-00857-9

40 Almoosawi S, Vingeliene S, Karagounis LG, *et al.* Chrono-nutrition: A review of current evidence from observational studies on global trends in time-of-day of energy intake and its association with obesity. *Proc Nutr Soc* 2016;**75**:487–500. doi:10.1017/S0029665116000306

41 Sutton EF, Beyl R, Early KS, *et al.* Early Time-Restricted Feeding Improves Insulin Sensitivity, Blood Pressure, and Oxidative Stress Even without Weight Loss in Men with Prediabetes. *Cell Metab* 2018;**27**:1212-1221.e3. doi:10.1016/j.cmet.2018.04.010

42 de Cabo R, Mattson MP. Effects of Intermittent Fasting on Health, Aging, and Disease. *N Engl J Med* 2019;**381**:2541–51. doi:10.1056/NEJMra1905136

43 Hnatiuk J, Ridgers ND, Salmon J, *et al.* Physical activity levels and patterns of 19-month-old children. *Med Sci Sports Exerc* Published Online First: 2012. doi:10.1249/MSS.0b013e31825825c4

44 Prioreschi A, Brage S, Hesketh KD, *et al.* Describing objectively measured physical activity levels, patterns, and correlates in a cross sectional sample of infants and toddlers from South Africa. *Int J Behav Nutr Phys Act* 2017;**14**:176. doi:10.1186/s12966-017-0633-5

45 Collings PJ, Brage S, Ridgway CL, *et al.* Physical activity intensity, sedentary time, and body composition in preschoolers. *Am J Clin Nutr* Published Online First: 2013. doi:10.3945/ajcn.112.045088

46 Collings PJ, Dogra SA, Costa S, *et al.* Objectively-measured sedentary time and physical activity in a bi-ethnic sample of young children: variation by socio-demographic, temporal and perinatal factors. *BMC Public Health* 2020;**20**:109. doi:10.1186/s12889-019-8132-z

47 Hesketh KR, McMinn AM, Ekelund U, *et al.* Objectively measured physical activity in four-year-old British children: a cross-sectional analysis of activity patterns segmented across the day. *Int J Behav Nutr Phys Act* 2014;**11**:1. doi:10.1186/1479-5868-11-1

48 Ward TM, Gay C, Anders TF, *et al.* Sleep and Napping Patterns in 3-to-5-year old Children Attending Full-Day Childcare Centers. *J Pediatr Psychol* 2008;**33**:666–72. doi:10.1093/jpepsy/jsm102

49 Staton SL, Smith SS, Hurst C, *et al.* Mandatory Nap Times and Group Napping Patterns in Child Care: An Observational Study. *Behav Sleep Med* 2017;**15**:129–43. doi:10.1080/15402002.2015.1120199

50 Staton S, Rankin PS, Harding M, *et al.* Many naps, one nap, none: A systematic review and meta-analysis of napping patterns in children 0–12 years. *Sleep Med Rev* 2020;**50**:101247. doi:10.1016/j.smrv.2019.101247

For peer review only

51 Dias K, White J, Jago R, *et al.* International Comparison of the Levels and Potential Correlates of Objectively Measured Sedentary Time and Physical Activity among Three-to-Four-Year-Old Children. *Int J Environ Res Public Health* 2019;**16**:1929. doi:10.3390/ijerph16111929

52 Matricciani LA, Olds TS, Blunden S, *et al.* Never Enough Sleep: A Brief History of Sleep Recommendations for Children. *Pediatrics* 2012;**129**:548–56. doi:10.1542/peds.2011-2039

53 Hudda MT, Nightingale CM, Donin AS, *et al.* Body mass index adjustments to increase the validity of body fatness assessment in UK Black African and South Asian children. *Int J Obes* 2017;**41**:1048–55. doi:10.1038/ijo.2017.75

54 Schoch SF, Kurth S, Werner H. Actigraphy in sleep research with infants and young children: Current practices and future benefits of standardized reporting. *J Sleep Res* 2020;:1–15. doi:10.1111/jsr.13134

55 Collings PJ, Wijndaele K, Corder K, *et al.* Prospective associations between sedentary time, sleep duration and adiposity in adolescents. *Sleep Med* 2015;**16**:717–22. doi:10.1016/j.sleep.2015.02.532

For peer review only

Figure 1. Associations of sleep onset categories with waist circumference and sum of 2-skinfolds, stratified by ethnicity and age group

Graphs on the left relate to South Asian children and graphs on the right to White children. Results are estimated marginal means (95% confidence intervals) adjusted for sex, age, socioeconomic status, maternal pregnancy age, parity, gestational age, birthweight, maternal BMI, season of measurement, napping frequency, unhealthy snacking, fruit and vegetable intake, TV viewing, and the sleep period. *Significantly different from the reference category of 7:30pm or earlier (β=-1.5 (-2.9 to -0.1) cm, *p*=0.035); significant difference persisted when further adjusted for final mealtime (*n*=103: β=-1.7 (-3.2 to -0.1) cm, *p*=0.036). †Some evidence for a difference compared to the reference category: (β=-1.8 (-3.8 to 0.3) cm, p=0.088). Results are presented for South Asian children aged 18m (9pm or earlier: waist circumference, *n*=25; skinfolds, *n*=19; Between 9 and 10pm: waist circumference, *n*=26; skinfolds, *n*=23; After 10pm: waist circumference, *n*=26; skinfolds, *n*=22) and 36m (9pm or earlier: waist circumference, *n*=22; skinfolds, *n*=16; Between 9 and 10pm: waist circumference, *n*=28; skinfolds, *n*=17; After 10pm: waist circumference, *n*=10; skinfolds, *n*=5), and White children aged 18m (7:30pm or earlier: waist circumference, *n*=39; skinfolds, *n*=30; Between 7:30 and 8:30pm: waist circumference, *n*=51; skinfolds, *n*=37; After 8:30pm: waist circumference, *n*=19; skinfolds, *n*=17) and 36m (7:30pm or earlier: waist circumference, *n*=36; skinfolds, *n*=25; Between 7:30 and 8:30pm: waist circumference, *n*=42; skinfolds, *n*=29; After 8:30pm: waist circumference, *n*=18; skinfolds, *n*=13).

Figure 2. Associations of weekday to weekend differences in sleep onset with waist circumference and sum of 2-skinfolds, stratified by ethnicity and age group

Graphs on the left relate to South Asian children and graphs on the right to White children. Results are estimated marginal means (95% confidence intervals) adjusted for sex, age, socioeconomic status, maternal pregnancy age, parity, gestational age, birthweight, maternal BMI, season of measurement, napping frequency, average weekday sleep onset, unhealthy snacking, fruit and vegetable intake, average weekday TV viewing, week to weekend differences in TV viewing, average weekday sleep period, and week to weekend differences in the sleep period. *Significantly different from the reference category of ±20 minutes (β=-1.7 (-3.2 to -0.1) cm, *p*=0.038). †Some evidence for a difference compared to the reference category: (β=1.5 (-0.1 to 3.0) cm, *p*=0.060). ‡Some evidence for a difference compared to the reference category: (β=1.9 (-0.2 to 4.1) mm, *p*=0.078). ¶Some evidence for a difference compared to the reference category: (β=2.0 (-0.2 to 4.3) mm, *p*=0.074). Results are presented for South Asian children aged 18m (≥20 minutes earlier: waist circumference, *n*=16; skinfolds, *n*=15; ±20 minutes: waist circumference, *n*=34; skinfolds, *n*=27; ≥20 minutes later: waist circumference, *n*=27; skinfolds, *n*=22) and 36m (≥20 minutes earlier: waist

circumference, *n*=10; skinfolds, *n*=3; ±20 minutes: waist circumference, *n*=28; skinfolds, *n*=21; ≥20 minutes later: waist circumference, *n*=22; skinfolds, *n*=14), and White children aged 18m (≥20 minutes earlier: waist circumference, *n*=20; skinfolds, *n*=16; ±20 minutes: waist circumference, *n*=60; skinfolds, *n*=46; ≥20 minutes later: waist circumference, *n*=29; skinfolds, *n*=22) and 36m (≥20 minutes earlier: waist circumference, *n*=19; skinfolds, *n*=14; ±20 minutes: waist circumference, *n*=47; skinfolds, *n*=35; ≥20 minutes later: waist circumference, *n*=30; skinfolds, *n*=18).

For peer review only

Table 1. Description of study participants

	South Asian		White	
	18 months (*n*=84)	36 months (*n*=66)	18 months (*n*=125)	36 months (*n*=96)
Sex (*n* (%) boys)	43 (51.2)	32 (48.5)	58 (46.4)	38 (39.6)
Age (months)	18.1 (0.7)	36.7 (1.1)	18.3 (1.1)	36.5 (0.5)
Parental socioeconomic status (*n* (%))				
Least deprived	22 (26.2)	15 (22.7)	32 (25.6)	30 (31.3)
Moderately deprived	29 (34.5)	20 (30.3)	70 (56.0)	53 (55.2)
Most deprived	33 (39.3)	31 (47.0)	23 (18.4)	13 (13.5)
Maternal ethnicity (*n* (%) Pakistani origin)	73 (86.9)	55 (83.3)	-	-
Maternal ethnicity (*n* (%) White British)	-	-	119 (95.2)	89 (92.7)
Maternal pregnancy age (y)	27.6 (7.3)	30.6 (7.6)	29.1 (10.3)	28.8 (8.2)
Parity (*n* (%) primaparous)	30 (35.7)	21 (31.8)	65 (52.0)	60 (62.5)
Birthweight (g)	3156 ± 465	3066 ± 552	3400 ± 543	3358 ± 563
Gestational age (weeks)	39.6 ± 1.6	39.4 ± 1.6	39.8 ± 1.5	39.6 ± 1.9
Season (*n* (%) summer / autumn)	71 (84.5)	22 (33.3)	108 (86.4)	18 (18.8)
Unhealthy snacks (*n*/week)	10 (10)	17 (23)	9 (8)	11 (12)
Fruit & vegetables (portions/day)	6 ± 2	6 ± 3	5 ± 2	5 ± 3
Physical activity (h/day)	-	2.8 ± 1.5	-	2.7 (2.0)
Maternal BMI (kg/m^2)	26.4 (6.3)	27.2 (6.6)	25.1 (8.0)	25.8 (7.0)
Napping frequency (*n* (%))				
Never	0 (0)	24 (36.4)	4 (3.2)	47 (49.0)
Occasionally	7 (8.3)	26 (39.4)	20 (16.0)	44 (45.8)
Everyday	77 (91.7)	16 (24.2)	101 (80.8)	5 (5.2)
Weighted daily average				
TV viewing (h/day)	1.0 (1.6)	1.5 (1.7)	0.6 (1.0)	1.5 (1.1)
Time of last meal (pm)	7:23 ± 66	7:20 ± 59	5:28 ± 51	5:32 ± 43
Sleep onset (pm)	9:36 ± 69	9:12 ± 63	7:50 ± 50	7:54 ± 44
Sleep offset (am)	8:21 ± 63	8:23 ± 63	7:11 ± 47	7:12 ± 50
Sleep period (h/night)	10.8 ± 1.0	11.2 ± 0.8	11.4 ± 0.8	11.3 ± 0.7
Sleep duration (h/night)	10.5 ± 1.0	11.0 ± 0.9	11.1 ± 1.1	11.3 ± 0.8
Weekday to weekend difference				
TV viewing (h/day)	-0.1 ± 0.6	0.0 ± 0.6	0.0 ± 0.6	0.0 ± 0.9
Time of last meal (h)	0.2 ± 0.7	0.2 ± 0.7	0.1 ± 0.8	0.2 ± 0.7
Sleep onset (h)	0.1 ± 0.6	0.3 ± 0.8	0.1 ± 0.9	0.1 ± 0.8
Sleep offset (h)	0.2 ± 0.9	0.3 ± 0.8	0.3 ± 0.8	0.2 ± 0.9
Sleep period (h/night)	0.1 ± 1.0	0.0 ± 0.8	0.2 ± 1.2	0.1 ± 1.0
Sleep duration (h/night)	0.0 ± 0.9	-0.1 ± 1.0	0.0 ± 1.0	0.0 ± 1.1
BMI (kg/m^2)	16.0 ± 1.3	15.8 ± 1.2	16.7 ± 1.2	16.4 ± 1.2
BMI *z*-score	-0.8 ± 1.1	-0.2 ± 1.0	-0.2 ± 0.9	0.3 ± 0.8
Waist circumference (cm)	44.9 ± 2.8	49.2 ± 3.1	46.3 ± 3.0	50.1 ± 3.1
Sum of 2-skinfolds (mm)	17.1 (3.8)	15.8 (3.4)	17.4 (4.3)	17.2 (4.8)

Normally distributed continuous variables are described as mean ± sd. Time of last meal, sleep onset and offset are described as clock times ± sd in minutes. Non-normally distributed continuous variables are described as median (IQR). Positive weekday to weekend differences indicate more TV viewing or sleep at the weekend than on weekdays, or later clock times at the weekend. There were missing data: Physical activity available for *n*=60 South Asian children at 36 months and *n*=89 White children at 36 months; Time of last meal available for *n*=71 South Asian children at 18 months, *n*=59 South Asian children at 36 months, *n*=119 White children at 18 months, *n*=91 White children at 36 months; Sleep duration available for *n*=63 South Asian children at 18 months, *n*=45 South Asian children at 36 months, *n*=108 White children at 18 months, *n*=90 White children at 36 months; Waist circumference available for *n*=77 South Asian children at 18 months, *n*=60 South Asian children at 36 months, *n*=109 White children at 18 months, *n*=96 White children at 36 months; Sum of 2-skinfolds available for *n*=64 South Asian children at 18 months, *n*=38 South Asian children at 36 months, *n*=84 White children at 18 months, *n*=67 White children at 36 months. BMI, Body mass index

For peer review only

Table 2. Associations of sleep onset, period and duration with adiposity in South Asian children, stratified by age group

		Body mass index *z*-score					Waist circumference (cm)					Sum of 2-skinfolds (mm)				
			Weighted daily average		Weekday to weekend difference			Weighted daily average		Weekday to weekend difference			Weighted daily average		Weekday to weekend difference	
		n	β (95% CI)	*p*	β (95% CI)	*p*	*n*	β (95% CI)	*p*	β (95% CI)	*p*	*n*	β (95% CI)	*p*	β (95% CI)	*p*
18 months	Model 1															
	Sleep onset	84	-0.1 (-0.3 to 0.2)	0.66	0.1 (-0.4 to 0.5)	0.76	77	-0.4 (-1.0 to 0.2)	0.24	0.8 (-0.3 to 1.8)	0.17	64	-0.2 (-1.1 to 0.7)	0.68	1.3 (-0.1 to 2.8)	0.070
	Sleep period	84	0.2 (-0.1 to 0.4)	0.16	0.0 (-0.3 to 0.3)	0.84	77	0.3 (-0.3 to 1.0)	0.30	0.0 (-0.7 to 0.8)	0.91	64	0.2 (-0.7 to 1.0)	0.70	-0.3 (-1.4 to 0.8)	0.56
	Sleep duration	63	0.2 (-0.2 to 0.5)	0.29	0.3 (-0.1 to 0.6)	0.17	58	0.3 (-0.6 to 1.2)	0.54	0.6 (-0.4 to 1.7)	0.21	49	0.5 (-0.7 to 1.6)	0.41	0.8 (-0.6 to 2.2)	0.26
	Model 2															
	Sleep onset	84	0.0 (-0.2 to 0.2)	0.93	0.0 (-0.4 to 0.4)	0.93	77	-0.3 (-0.9 to 0.4)	0.41	0.7 (-0.4 to 1.8)	0.21	64	-0.1 (-0.9 to 0.8)	0.90	**1.6 (0.1 to 3.1)**	**0.036**
	Sleep period	84	0.2 (-0.1 to 0.4)	0.22	0.1 (-0.2 to 0.4)	0.59	77	0.3 (-0.4 to 1.0)	0.39	0.3 (-0.5 to 1.1)	0.47	64	0.1 (-0.8 to 1.0)	0.89	-0.2 (-1.4 to 0.9)	0.72
	Sleep duration	63	0.2 (-0.1 to 0.5)	0.27	0.3 (-0.0 to 0.7)	0.066	58	0.3 (-0.6 to 1.2)	0.55	0.9 (-0.1 to 1.9)	0.075	49	0.3 (-0.9 to 1.5)	0.61	0.9 (-0.6 to 2.3)	0.23
	Model 3															
	Sleep onset[1]	84	0.1 (-0.2 to 0.4)	0.40	0.0 (-0.5 to 0.5)	0.99	77	-0.2 (-0.9 to 0.6)	0.65	1.2 (-0.1 to 2.4)	0.076	64	0.0 (-1.1 to 1.1)	0.96	**1.8 (0.1 to 3.5)**	**0.034**
	Sleep onset[2]	63	0.2 (-0.1 to 0.5)	0.23	0.4 (-0.1 to 0.8)	0.12	58	0.2 (-0.6 to 1.1)	0.58	**1.8 (0.6 to 2.9)**	**0.004**	49	0.0 (-1.0 to 1.1)	0.92	**1.7 (0.3 to 3.1)**	**0.022**
	Sleep period	84	0.2 (-0.1 to 0.5)	0.14	0.1 (-0.2 to 0.4)	0.63	77	0.2 (-0.6 to 1.0)	0.60	0.7 (-0.2 to 1.5)	0.14	64	0.0 (-1.1 to 1.1)	0.93	0.3 (-0.9 to 1.5)	0.61
	Sleep duration	63	0.3 (-0.1 to 0.6)	0.14	**0.4 (0.1 to 0.8)**	**0.024**	58	0.3 (-0.6 to 1.3)	0.46	**1.2 (0.3 to 2.2)**	**0.012**	49	0.3 (-0.9 to 1.5)	0.61	1.4 (-0.0 to 2.9)	0.056
36 months	Model 1															
	Sleep onset	66	0.1 (-0.1 to 0.4)	0.17	-0.3 (-0.6 to 0.0)	0.085	60	-0.1 (-1.0 to 0.7)	0.74	**-1.2 (-2.4 to -0.0)**	**0.048**	38	-0.3 (-1.3 to 0.7)	0.52	0.9 (-0.3 to 2.1)	0.14
	Sleep period	66	0.1 (-0.2 to 0.4)	0.50	0.2 (-0.1 to 0.5)	0.14	60	-0.4 (-1.7 to 0.8)	0.48	**1.1 (0.0 to 2.3)**	**0.047**	38	0.0 (-1.2 to 1.3)	0.96	-0.3 (-1.8 to 1.1)	0.64
	Sleep duration	45	0.2 (-0.1 to 0.5)	0.15	-0.0 (-0.4 to 0.3)	0.82	42	0.5 (-0.6 to 1.5)	0.35	0.3 (-1.0 to 1.6)	0.63	26	0.2 (-0.9 to 1.2)	0.71	-0.1 (-1.8 to 1.6)	0.92
	Model 2															
	Sleep onset	66	0.2 (-0.1 to 0.4)	0.13	-0.2 (-0.6 to 0.1)	0.15	60	0.1 (-0.8 to 1.0)	0.86	-1.0 (-2.2 to 0.2)	0.11	38	-0.2 (-1.4 to 1.0)	0.75	**1.3 (0.1 to 2.5)**	**0.033**
	Sleep period	66	0.1 (-0.2 to 0.5)	0.42	0.2 (-0.1 to 0.5)	0.14	60	-0.4 (-1.7 to 0.9)	0.55	**1.2 (0.1 to 2.3)**	**0.032**	38	0.1 (-1.2 to 1.4)	0.84	-1.1 (-2.7 to 0.4)	0.15
	Sleep duration	45	0.2 (-0.1 to 0.5)	0.27	0.1 (-0.3 to 0.5)	0.67	42	0.3 (-0.8 to 1.4)	0.55	0.5 (-0.8 to 1.9)	0.44	26	-0.0 (-1.1 to 1.0)	0.97	-0.8 (-3.1 to 1.5)	0.43
	Model 3															
	Sleep onset[1]	**66**	**0.3 (0.0 to 0.5)**	**0.042**	-0.2 (-0.6 to 0.2)	0.27	60	0.0 (-0.9 to 0.9)	0.97	-0.2 (-1.7 to 1.3)	0.80	38	-0.2 (-1.5 to 1.1)	0.79	1.2 (-0.3 to 2.8)	0.12
	Sleep onset[2]	45	0.1 (-0.1 to 0.4)	0.34	-0.2 (-0.8 to 0.3)	0.43	42	-0.3 (-1.2 to 0.6)	0.53	0.0 (-1.9 to 2.0)	0.95	26	-0.8 (-2.2 to 0.7)	0.25	0.4 (-2.3 to 3.1)	0.72
	Sleep period	66	0.3 (-0.1 to 0.6)	0.12	0.1 (-0.2 to 0.5)	0.42	60	-0.4 (-1.8 to 1.0)	0.57	1.1 (-0.3 to 2.5)	0.11	38	0.1 (-1.3 to 1.5)	0.89	-0.4 (-2.2 to 1.4)	0.68
	Sleep duration	45	0.2 (-0.1 to 0.5)	0.23	0.0 (-0.4 to 0.4)	0.85	42	0.3 (-0.8 to 1.4)	0.55	0.5 (-0.9 to 2.0)	0.45	26	-0.1 (-1.2 to 0.9)	0.80	-0.2 (-3.4 to 2.9)	0.85

Results are beta-coefficients (95% confidence intervals) and represent the difference in outcomes for every one hour later to sleep, one hour longer sleep period or duration, one hour later to sleep on weekends than weekdays, or one hour longer sleep period or duration on weekends than weekdays. Model 1 was adjusted for sex, age, socioeconomic status, maternal pregnancy age, parity, gestational age, birthweight, maternal body mass index, season of measurement, and napping frequency (models for weekday to weekend differences were further adjusted for the average weekday value of the exposure variable). Model 2 was further adjusted for unhealthy snacking, fruit and vegetable intake, and TV viewing (models for weekday to weekend sleep differences were adjusted for average weekday TV viewing and weekday to weekend differences in TV viewing). Model 3 further adjusted sleep period and duration variables for sleep onset, and vice versa (models for weekday to weekend differences were mutually adjusted for average weekday and weekday to weekend differences in the sleep period, duration or onset as appropriate). [1]Adjusted for the sleep period; [2]Adjusted for sleep duration. Statistically significant results (p<0.05) are highlighted bold. Sensitivity analyses confirmed that adjustment for physical activity (collected at 36 months only) did not confound Model 3 results, but the association between weighted daily average sleep onset with body mass index z-score at 36 months was attenuated when adjusted for time of last meal (body mass index z-score (n=59): 0.2 (-0.2 to 0.6), p=0.34).

For peer review only

Table 3. Associations of sleep onset, period and duration with adiposity in White children, stratified by age group

		Body mass index *z*-score					Waist circumference (cm)					Sum of 2-skinfolds (mm)				
			Weighted daily average		Weekday to weekend difference			Weighted daily average		Weekday to weekend difference			Weighted daily average		Weekday to weekend difference	
		n	β (95% CI)	*p*	β (95% CI)	*p*	*n*	β (95% CI)	*p*	β (95% CI)	*p*	*n*	β (95% CI)	*p*	β (95% CI)	*p*
18 months	Model 1															
	Sleep onset	125	-0.2 (-0.4 to 0.0)	0.086	0.0 (-0.2 to 0.2)	0.70	109	-0.5 (-1.2 to 0.2)	0.18	-0.6 (-1.4 to 0.2)	0.17	84	-1.0 (-2.1 to 0.0)	0.061	-0.1 (-1.2 to 1.0)	0.88
	Sleep period	125	0.0 (-0.2 to 0.2)	0.83	0.1 (-0.1 to 0.2)	0.44	109	0.2 (-0.6 to 0.9)	0.64	0.1 (-0.5 to 0.7)	0.70	84	0.1 (-1.0 to 1.2)	0.82	-0.1 (-0.9 to 0.8)	0.86
	Sleep duration	108	0.0 (-0.2 to 0.2)	0.91	0.1 (-0.1 to 0.3)	0.37	93	0.1 (-0.7 to 1.0)	0.74	0.3 (-0.4 to 1.1)	0.39	76	-0.1 (-1.0 to 0.8)	0.82	0.1 (-0.9 to 1.1)	0.85
	Model 2															
	Sleep onset	125	-0.2 (-0.4 to 0.0)	0.11	0.1 (-0.1 to 0.3)	0.39	109	-0.5 (-1.2 to 0.2)	0.18	-0.5 (-1.3 to 0.3)	0.23	84	-1.0 (-2.1 to 0.0)	0.060	0.1 (-1.0 to 1.2)	0.85
	Sleep period	125	0.0 (-0.2 to 0.2)	0.92	0.0 (-0.1 to 0.2)	0.81	109	0.1 (-0.6 to 0.9)	0.71	0.0 (-0.6 to 0.6)	0.99	84	0.1 (-1.0 to 1.1)	0.91	-0.3 (-1.2 to 0.5)	0.45
	Sleep duration	108	0.0 (-0.2 to 0.2)	0.99	0.0 (-0.1 to 0.2)	0.62	93	0.2 (-0.6 to 1.1)	0.57	0.2 (-0.6 to 1.1)	0.55	76	-0.1 (-1.0 to 0.8)	0.86	-0.2 (-1.3 to 0.9)	0.74
	Model 3															
	Sleep onset[1]	125	-0.2 (-0.5 to 0.0)	0.072	0.2 (-0.1 to 0.4)	0.15	109	-0.6 (-1.5 to 0.3)	0.18	-0.7 (-1.7 to 0.3)	0.15	**84**	**-1.5 (-2.8 to -0.2)**	**0.025**	-0.2 (-1.6 to 1.2)	0.72
	Sleep onset[2]	**108**	**-0.3 (-0.5 to -0.0)**	**0.044**	0.0 (-0.2 to 0.3)	0.79	93	-0.6 (-1.6 to 0.3)	0.20	-0.5 (-1.4 to 0.4)	0.28	**76**	**-1.5 (-2.9 to -0.0)**	**0.046**	-0.0 (-1.3 to 1.2)	0.97
	Sleep period	125	-0.1 (-0.3 to 0.1)	0.41	0.1 (-0.1 to 0.3)	0.31	109	-0.2 (-1.0 to 0.7)	0.69	-0.3 (-1.0 to 0.4)	0.40	84	-0.8 (-2.1 to 0.5)	0.22	-0.6 (-1.6 to 0.4)	0.25
	Sleep duration	108	-0.1 (-0.2 to 0.1)	0.49	0.1 (-0.1 to 0.3)	0.48	93	0.0 (-0.9 to 0.9)	0.99	0.1 (-0.8 to 0.9)	0.88	76	-0.6 (-1.6 to 0.4)	0.23	-0.3 (-1.5 to 0.8)	0.58
36 months	Model 1															
	Sleep onset	96	0.0 (-0.2 to 0.3)	0.71	-0.1 (-0.3 to 0.1)	0.32	96	0.2 (-0.7 to 1.1)	0.63	-0.1 (-0.9 to 0.8)	0.89	67	-0.3 (-1.6 to 0.9)	0.57	-0.6 (-1.6 to 0.5)	0.29
	Sleep period	96	0.0 (-0.3 to 0.3)	0.98	-0.0 (-0.2 to 0.2)	0.91	96	-0.5 (-1.6 to 0.5)	0.31	-0.3 (-1.1 to 0.5)	0.40	67	-0.2 (-1.6 to 1.2)	0.82	-0.1 (-1.1 to 0.9)	0.83
	Sleep duration	90	-0.1 (-0.3 to 0.1)	0.36	-0.0 (-0.2 to 0.2)	0.98	90	-0.5 (-1.4 to 0.4)	0.26	-0.1 (-0.9 to 0.7)	0.81	62	-0.1 (-1.6 to 1.3)	0.88	0.3 (-0.8 to 1.5)	0.54
	Model 2															
	Sleep onset	96	0.0 (-0.2 to 0.3)	0.87	-0.1 (-0.4 to 0.1)	0.38	96	0.1 (-0.9 to 1.0)	0.90	-0.0 (-1.0 to 0.9)	0.92	67	-0.3 (-1.6 to 1.0)	0.68	-0.9 (-2.1 to 0.2)	0.12
	Sleep period	96	0.1 (-0.2 to 0.4)	0.66	0.0 (-0.2 to 0.2)	0.81	96	-0.4 (-1.5 to 0.8)	0.51	-0.3 (-1.1 to 0.6)	0.54	67	-0.1 (-1.6 to 1.3)	0.86	0.1 (-1.0 to 1.1)	0.89
	Sleep duration	90	-0.1 (-0.3 to 0.2)	0.55	0.1 (-0.2 to 0.3)	0.55	90	-0.4 (-1.3 to 0.5)	0.37	0.1 (-0.8 to 1.0)	0.81	62	-0.2 (-1.7 to 1.3)	0.79	0.5 (-0.8 to 1.8)	0.44
	Model 3															
	Sleep onset[1]	96	0.0 (-0.2 to 0.3)	0.79	-0.1 (-0.4 to 0.1)	0.33	96	0.0 (-1.0 to 1.0)	0.98	-0.2 (-1.3 to 0.9)	0.71	67	-0.3 (-1.6 to 1.0)	0.67	-1.3 (-2.8 to 0.1)	0.074
	Sleep onset[2]	90	0.0 (-0.3 to 0.3)	0.98	-0.0 (-0.3 to 0.2)	0.72	90	0.0 (-1.0 to 1.0)	0.98	0.1 (-0.9 to 1.1)	0.83	62	-0.4 (-1.9 to 1.2)	0.65	-1.4 (-3.1 to 0.2)	0.085
	Sleep period	96	0.1 (-0.2 to 0.4)	0.63	-0.0 (-0.3 to 0.2)	0.76	96	-0.4 (-1.6 to 0.8)	0.52	-0.3 (-1.3 to 0.6)	0.48	67	-0.2 (-1.6 to 1.3)	0.83	-0.6 (-2.0 to 0.7)	0.33
	Sleep duration	90	-0.1 (-0.3 to 0.2)	0.55	0.1 (-0.2 to 0.3)	0.68	90	-0.4 (-1.4 to 0.5)	0.37	0.1 (-0.8 to 1.1)	0.76	62	-0.3 (-1.8 to 1.3)	0.74	-0.3 (-2.0 to 1.3)	0.67

Results are beta-coefficients (95% confidence intervals) and represent the difference in outcomes for every one hour later to sleep, one hour longer sleep period or duration, one hour later to sleep on weekends than weekdays, or one hour longer sleep period or duration on weekends than weekdays. Model 1 was adjusted for sex, age, socioeconomic status, maternal pregnancy age, parity, gestational age, birthweight, maternal body mass index, season of measurement, and napping frequency (models for weekday to weekend differences were further adjusted for the average weekday value of the exposure variable). Model 2 was further adjusted for unhealthy snacking, fruit and vegetable intake, and TV viewing (models for weekday to weekend sleep differences were adjusted for average weekday TV viewing and weekday to weekend differences in TV viewing). Model 3 further adjusted sleep period and duration variables for sleep onset, and vice versa (models for weekday to weekend differences were mutually adjusted for average weekday and weekday to weekend differences in the sleep period, duration or onset as appropriate). [1]Adjusted for the sleep period; [2]Adjusted for sleep duration. Statistically significant results (p<0.05) are highlighted bold. Sensitivity analyses confirmed that adjustment for physical activity (collected at 36 months only) did not confound Model 3 results, but the association between weighted daily average sleep onset with body mass index *z*-score at 18 months was attenuated when adjusted for time of last meal (BMI *z*-score (n=101): -0.2 (-0.5 to 0.2), p=0.28).

For peer review only

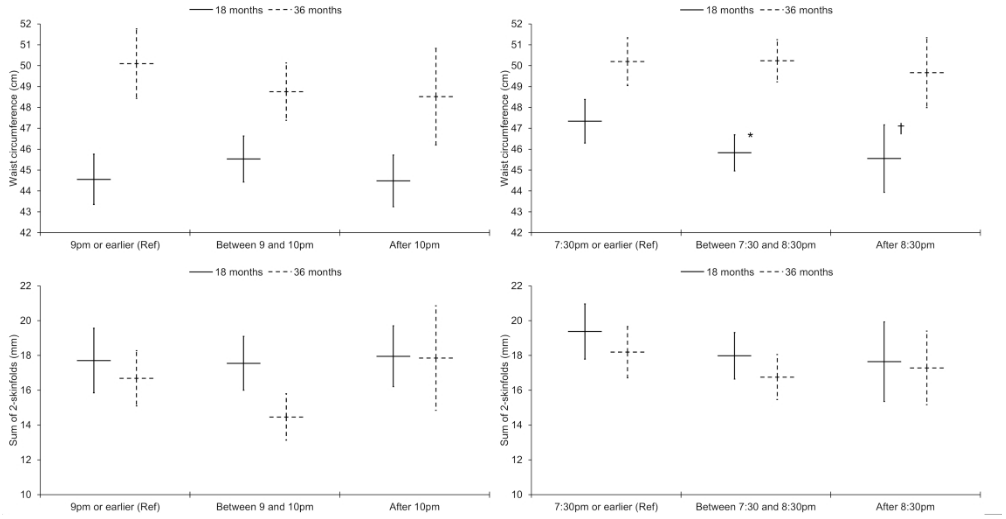

Figure 1. Associations of sleep onset categories with waist circumference and sum of 2-skinfolds, stratified by ethnicity and age group

332x171mm (300 x 300 DPI)

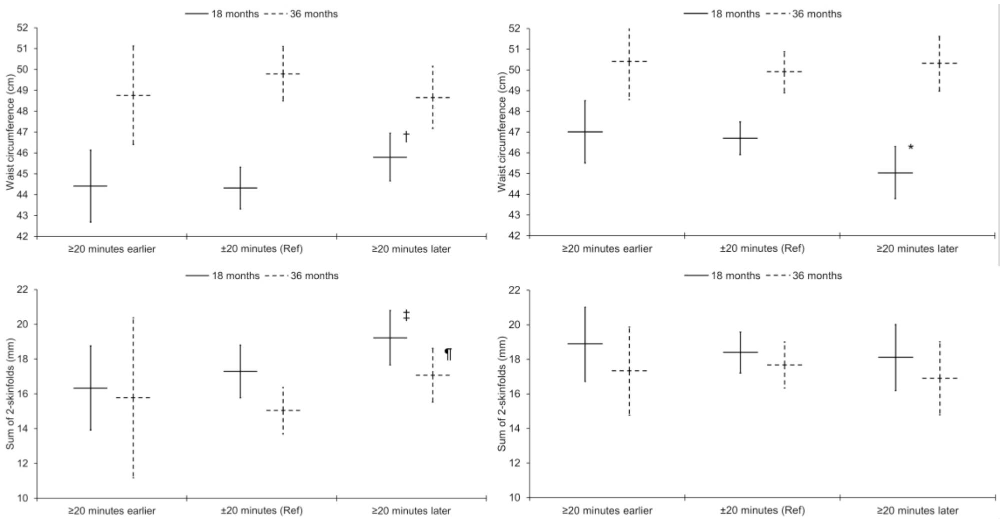

Figure 2. Associations of weekday to weekend differences in sleep onset with waist circumference and sum of 2-skinfolds, stratified by ethnicity and age group

332x171mm (300 x 300 DPI)

STROBE Statement—Checklist of items that should be included in reports of ***cohort studies***

	Item No	Recommendation	Page No
**Title and abstract**	1	(*a*) Indicate the study's design with a commonly used term in the title or the abstract	3
		(*b*) Provide in the abstract an informative and balanced summary of what was done and what was found	3
**Introduction**			
Background/rationale	2	Explain the scientific background and rationale for the investigation being reported	5
Objectives	3	State specific objectives, including any prespecified hypotheses	6
**Methods**			
Study design	4	Present key elements of study design early in the paper	6
Setting	5	Describe the setting, locations, and relevant dates, including periods of recruitment, exposure, follow-up, and data collection	6
Participants	6	(*a*) Give the eligibility criteria, and the sources and methods of selection of participants. Describe methods of follow-up	6
		(*b*) For matched studies, give matching criteria and number of exposed and unexposed	N/A
Variables	7	Clearly define all outcomes, exposures, predictors, potential confounders, and effect modifiers. Give diagnostic criteria, if applicable	6-8
Data sources/ measurement	8*	For each variable of interest, give sources of data and details of methods of assessment (measurement). Describe comparability of assessment methods if there is more than one group	6-8
Bias	9	Describe any efforts to address potential sources of bias	6-8
Study size	10	Explain how the study size was arrived at	6
Quantitative variables	11	Explain how quantitative variables were handled in the analyses. If applicable, describe which groupings were chosen and why	8
Statistical methods	12	(*a*) Describe all statistical methods, including those used to control for confounding	8
		(*b*) Describe any methods used to examine subgroups and interactions	8
		(*c*) Explain how missing data were addressed	6
		(*d*) If applicable, explain how loss to follow-up was addressed	N/A
		(*e*) Describe any sensitivity analyses	8
**Results**			
Participants	13*	(a) Report numbers of individuals at each stage of study—eg numbers potentially eligible, examined for eligibility, confirmed eligible, included in the study, completing follow-up, and analysed	6
		(b) Give reasons for non-participation at each stage	6
		(c) Consider use of a flow diagram	N/A
Descriptive data	14*	(a) Give characteristics of study participants (eg demographic, clinical, social) and information on exposures and potential confounders	9 Tables
		(b) Indicate number of participants with missing data for each variable of interest	Table 1
		(c) Summarise follow-up time (eg, average and total amount)	N/A
Outcome data	15*	Report numbers of outcome events or summary measures over time	N/A

For peer review only - http://bmjopen.bmj.com/site/about/guidelines.xhtml

Main results	16	(*a*) Give unadjusted estimates and, if applicable, confounder-adjusted estimates and their precision (eg, 95% confidence interval). Make clear which confounders were adjusted for and why they were included	9-10 Tables Figures
		(*b*) Report category boundaries when continuous variables were categorized	N/A
		(*c*) If relevant, consider translating estimates of relative risk into absolute risk for a meaningful time period	N/A
Other analyses	17	Report other analyses done—eg analyses of subgroups and interactions, and sensitivity analyses	11
**Discussion**			
Key results	18	Summarise key results with reference to study objectives	10
Limitations	19	Discuss limitations of the study, taking into account sources of potential bias or imprecision. Discuss both direction and magnitude of any potential bias	12-14
Interpretation	20	Give a cautious overall interpretation of results considering objectives, limitations, multiplicity of analyses, results from similar studies, and other relevant evidence	13-14
Generalisability	21	Discuss the generalisability (external validity) of the study results	14
**Other information**			
Funding	22	Give the source of funding and the role of the funders for the present study and, if applicable, for the original study on which the present article is based	2

*Give information separately for exposed and unexposed groups.

Note: An Explanation and Elaboration article discusses each checklist item and gives methodological background and published examples of transparent reporting. The STROBE checklist is best used in conjunction with this article (freely available on the Web sites of PLoS Medicine at http://www.plosmedicine.org/, Annals of Internal Medicine at http://www.annals.org/, and Epidemiology at http://www.epidem.com/). Information on the STROBE Initiative is available at http://www.strobe-statement.org.

For peer review only
